# Multimorbidity prevalence and health outcome prediction: assessing the impact of lookback periods, disease count, and definition criteria in health administrative data at the population-based level

**DOI:** 10.1186/s12874-024-02243-0

**Published:** 2024-05-16

**Authors:** Marc Simard, Elham Rahme, Marjolaine Dubé, Véronique Boiteau, Denis Talbot, Caroline Sirois

**Affiliations:** 1 Institut national de santé publique du Québec, 945, Wolfe, 5e étage Québec, Québec, QC G1V 5B3 Canada; 2 Department of social and preventive medicine, Faculty of Medicine, Université Laval, Québec, QC Canada; 3 Centre de recherche du CHU de Québec, Québec, QC Canada; 4 VITAM-Centre de recherche en santé durable, Québec, QC Canada; 5 The Research Institute of the McGill University Health Centre, Montréal, QC Canada; 6 Faculty of Pharmacy, Université Laval, Québec, QC Canada

**Keywords:** Multimorbidity, Prevalence, Health outcome prediction, Lookback period, Administrative Data

## Abstract

**Background:**

Health administrative databases play a crucial role in population-level multimorbidity surveillance. Determining the appropriate retrospective or lookback period (LP) for observing prevalent and newly diagnosed diseases in administrative data presents challenge in estimating multimorbidity prevalence and predicting health outcome. The aim of this population-based study was to assess the impact of LP on multimorbidity prevalence and health outcomes prediction across three multimorbidity definitions, three lists of diseases used for multimorbidity assessment, and six health outcomes.

**Methods:**

We conducted a population-based study including all individuals ages > 65 years on April 1st, 2019, in Québec, Canada. We considered three lists of diseases labeled according to the number of chronic conditions it considered: (1) L60 included 60 chronic conditions from the International Classification of Diseases (ICD); (2) L20 included a core of 20 chronic conditions; and (3) L31 included 31 chronic conditions from the Charlson and Elixhauser indices. For each list, we: (1) measured multimorbidity prevalence for three multimorbidity definitions (at least two [MM2+], three [MM3+] or four (MM4+) chronic conditions); and (2) evaluated capacity (*c-statistic*) to predict 1-year outcomes (mortality, hospitalisation, polypharmacy, and general practitioner, specialist, or emergency department visits) using LPs ranging from 1 to 20 years.

**Results:**

Increase in multimorbidity prevalence decelerated after 5–10 years (e.g., MM2+, L31: LP = 1y: 14%, LP = 10y: 58%, LP = 20y: 69%). Within the 5–10 years LP range, predictive performance was better for L20 than L60 (e.g., LP = 7y, mortality, MM3+: L20 [0.798;95%CI:0.797-0.800] vs. L60 [0.779; 95%CI:0.777–0.781]) and typically better for MM3 + and MM4 + definitions (e.g., LP = 7y, mortality, L60: MM4+ [0.788;95%CI:0.786–0.790] vs. MM2+ [0.768;95%CI:0.766–0.770]).

**Conclusions:**

In our databases, ten years of data was required for stable estimation of multimorbidity prevalence. Within that range, the L20 and multimorbidity definitions MM3 + or MM4 + reached maximal predictive performance.

**Supplementary Information:**

The online version contains supplementary material available at 10.1186/s12874-024-02243-0.

## Background

Multimorbidity is a complex condition associated with poor health outcomes, polypharmacy, and high healthcare utilisation [1]. It is particularly prevalent in older adults [≥ 65 years old], with more than half cumulating two or more chronic conditions [2]. The most common criteria used to define multimorbidity (MM) are based on the count of different chronic conditions [3], such as at least two (MM2+), three (MM3+) or four (MM4+) chronic diseases. From a public health perspective, multimorbidity measures based on the count of chronic conditions are useful to decision makers who must consider multiple health outcomes simultaneously to plan appropriate interventions [4]. Moreover, because of their simplicity and ease of interpretation, they are gaining popularity among clinicians and the lay public [4].

Health administrative databases are extensively used for surveillance and research purposes to measure multimorbidity prevalence at the population level, particularly in single-payer healthcare systems such as those in Australia, Canada, UK, Taiwan and many European countries [5]. Yet the creation of multimorbidity measures based on the count of chronic conditions entails several methodological choices that can affect their validity and predictive performance. For one, the length of the optimal retrospective period of search for relevant healthcare encounters remains an issue. Health administrative databases comprise a sequential collection of codes for prevalent and newly diagnosed diseases captured in one or many data files during medical visits or hospitalisation stays. Therefore, a minimal retrospective period of search for diagnosis codes (“lookback period” [LP]) is required to accurately capture the chronic conditions of each person registered. A LP that is too short may underestimate the prevalence of multimorbidity, while a LP that is too long may complicate data extraction and increase the probability of erroneously capturing resolved conditions from previous years.

The choice of LP may therefore impact both the prevalence of multimorbidity and its capacity to predict health outcomes; however, these elements have never been jointly assessed. Some studies have assessed the impact of the LP on the prevalence of multimorbidity. Among Danish adults aged 65 and over in 2015, the prevalence of MM2 + increased from 10 to 52% as the LP increased from 1 to 15 years, with a relative stabilization around 10 years [6]. Higher prevalence with increasing LP was also observed among Canadian patients hospitalized in the early 2000s for cardiovascular diseases [7] and or HIV [8]. Other studies have assessed outcome predictions in association with LP. In Canadian patients newly diagnosed with hypertension in the early 2000s, the performance of the MM2 + criterion in predicting 1-year mortality increased when the LP was extended from 6 to 12 months, with *c-statistic* values increasing from 0.89 to 0.91 [9]. In a Australian cohort of hospitalized patients between 1990 and 1996, increasing the LP from 1 to 5 years resulted in a small increase in the prediction of 30-day readmission (*c-statistic* values increasing from 0.67 to 0.68) but had no impact on 1-year mortality (*c-statistic* remaining unchanged at 0.90) [10]. However, the latter studies had several shortcomings when assessing outcome prediction: (1) maximal LP was limited to 5 years; (2) analyses were conducted in subgroups and not in the general population; (3) some but not all health outcomes considered of interest were evaluated. In addition, no study has previously assessed jointly the prevalence of multimorbidity and its predictive performance according to the LP.

Both the multimorbidity prevalence and the capacity of multimorbidity to predict health outcomes are influenced by two elements: (1) the number of diseases included in the multimorbidity measure, and (2) the criterion used to define multimorbidity (e.g., MM2+, MM3+, MM4+) [11, 12]. However, it remains unclear how the LP interact with these two aspects and thus affect multimorbidity prevalence and predictive capacity.

### Objectives

The primary objective of this study was to evaluate the impact of the LP on the prevalence of multimorbidity and the prediction of six health outcomes in the general population among individuals over 65 years of age. The secondary objective was to assess whether variations in the list of diseases included in the multimorbidity measure or the choice of criterion used to define multimorbidity can influence the impact of LP in this population.

## Methods

### Data source and population

Our population-based cohort study included all individuals over the age of 65 registered in the Québec Integrated Chronic Disease Surveillance System (QICDSS) on April 1st, 2019, (cohort entry date) and followed them for one year. The QICDSS links provincial health services administrative data since 1996 using a unique patient identifier [13]. The data include demographic, death registry, physician claims, and pharmaceutical claims records obtained from the Provincial health insurance board (Régie de l’assurance maladie du Québec [RAMQ]) as well has hospital discharge abstract records (MED-ECHO) owned by the Quebec Ministry of Health and housed at RAMQ. Demographic data includes place of residence, age, sex and neighbourhood-level social and material deprivation quintiles [14]. Physician claims include diagnoses coded using the International Classification of Diseases, 9th Revision, Quebec adaptation (ICD-9-QC) and the ICD 10th Revision Canadian Coding Standard (ICD-10-CA) since January 1st, 2019. Hospital discharge records include the admission diagnosis, primary diagnosis and up to 29 secondary diagnoses coded using ICD-9-QC system until March 31, 2006, and ICD-10-CA system thereafter. As the province of Quebec has a universal healthcare system, the QICDSS includes medical records for over 99% of the population. In addition, drug insurance is mandatory in Quebec. All individuals aged 65 years and older are eligible for coverage by the public drug plan. However, approximately 10% is not covered due to either their preference to retain their private insurance plan or their medication being provided by the nursing home where they reside.

### Multimorbidity measure

We considered three widely used criteria to define multimorbidity: MM2+, MM3+, MM4+ [3]. We also identified three lists of medical conditions commonly used to build the multimorbidity measures. These lists were deemed representative of the high diversity of medical conditions included in multimorbidity measures relying on health administrative data [5] (The lists of diseases and ICD codes for each list are available in Supplemental Digital Content [SDC] 01: Tables A1.1-A1.4). First, the “All-inclusive list”(L60) included all ICD codes corresponding to chronic diseases grouped into 60 diseases by a multidisciplinary team [15]. This list was considered of high quality in a previous systematic review because it met six of the eight quality criteria used to define robust multimorbidity measures methodology [5]. Second, the “Core list” (L20) included a minimal core of 20 diseases identified in a systematic review by Ho and colleagues [3]. This minimum core of diseases includes chronic conditions with the highest disability adjusted life-years (DALYs) or years of life lost (YLLs) from the Global Burden of Disease Project [16]. We added osteoporosis to that list because this chronic condition was reported among the top 20 with the highest impact on DALY in Canada [17]. Third, the “Charlson & Elixhauser list” (L31) included 31 diseases from the Combined comorbidity index, a combination of both Charlson and Elixhauser comorbidity indices [11, 12].

**Fig. 1:**
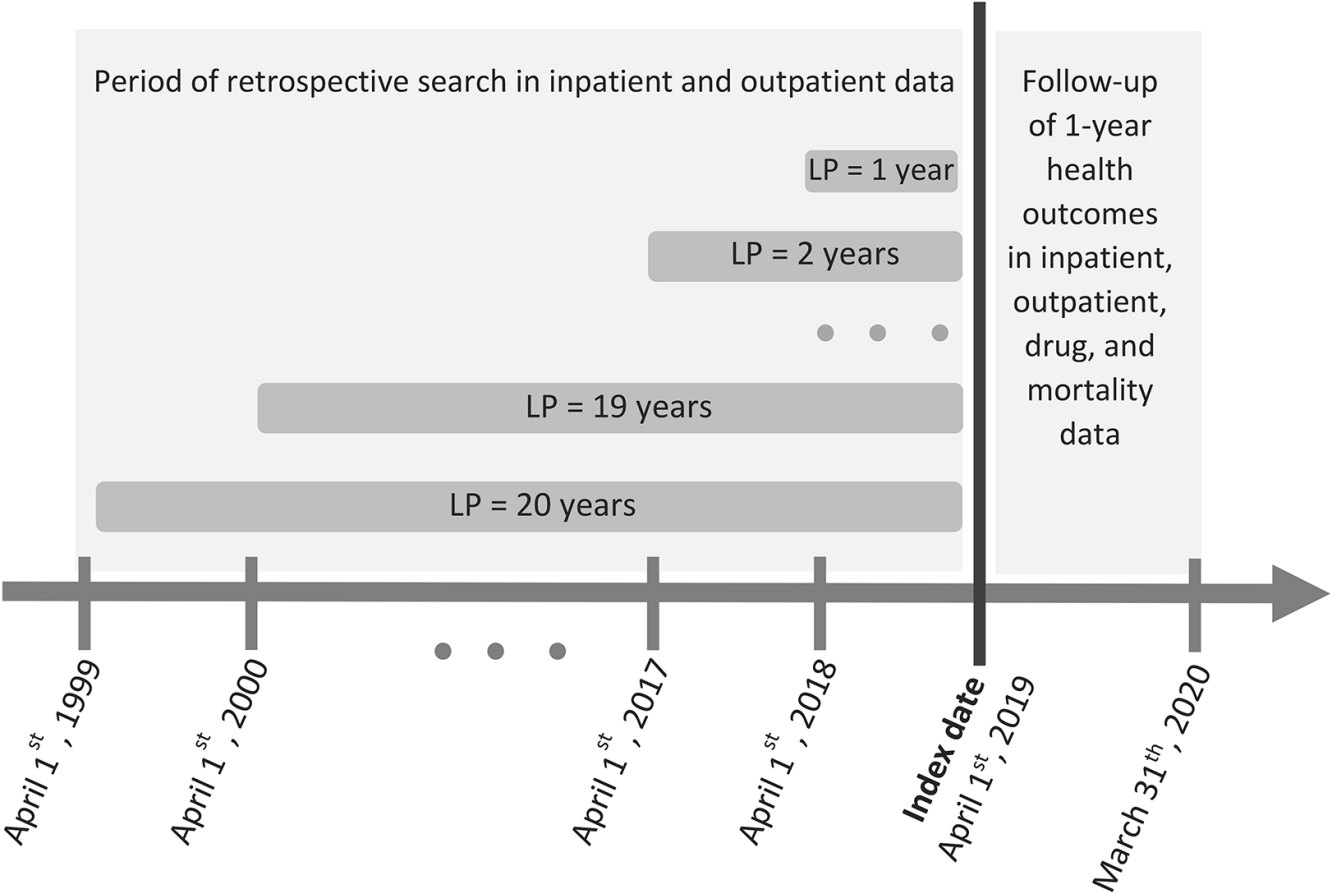
Illustration of the assessment of multimorbidity prevalence at index date with varied lookback periods (LPs) and 1-year health outcome measurements. For example, for a person aged 66 on April 1^st^, 2019, the retrospective search in both inpatient and outpatient databases using a LP of 1 year runs from April 1^st^, 2018 to March 31^th^, 2019. Using a LP of 20 years, it extends from April 1^st^ 1999 to March 31^th^, 2019

We employed varying LP ranging from 1 to 20 years to estimate multimorbidity prevalence at the cohort entry date (April 1, 2019). We retrospectively retrieved ICD diagnosis codes for each person and medical condition from hospitalization and physician records until April 1st, 1999 (Fig. 1). The choice of a 20-year LP was based on the availability of data in QICDSS, limiting our analysis to this timeframe. We used the algorithm proposed by Klabunde et al. [18] to identify each disease in the administrative databases: we searched both inpatient and outpatient records and identified an individual as having a disease if (1) at least one diagnosis code (primary or secondary) was recorded in the hospitalization records or (2) at least two diagnosis codes were recorded in inpatient or outpatient physician claims within two years and at least 30 days apart.

### Outcomes

We investigated the capacity of each multimorbidity measure, computed on April 1st, 2019, to predict six health outcomes that have been associated with multimorbidity and were measurable in the QICDSS during the 1-year follow-up (until March 31th, 2020): all-cause mortality, polypharmacy, hospitalisation and frequent visits to the emergency department (ED), to the general practitioner (GP) and to any specialist physician (SP). We defined polypharmacy as ≥ 10 different medications claimed in the follow-up year. We used the common denomination (each active ingredient or combination has a distinct common denomination code) to identify each medication claimed. Those claims included medications for acute and chronic conditions. We defined frequent ED visits using a commonly used threshold of ≥ 3 visits in the follow-up year [19]. A single visit to the ED was defined as 1 or more ED–related claims on up to 2 consecutive days [20]. Frequent visits to any GP (≥ 7 visits) or any SP (≥ 10 visits) in the follow-up year were defined using the 95th percentile in the annual number of ED visits in the Québec adult population [21, 22].

### Statistical analysis

We estimated the prevalence of multimorbidity for each criterion used to define multimorbidity, each list of diseases, and each LP (1 to 20 years) and calculated the relative change in multimorbidity prevalence for each additional year of lookback (Fig. 1).

Then, we used logistic regression models to assess the impact of each criterion used to define multimorbidity on the health outcome. We first built one baseline model for each health outcome where the health outcome was the dependent variable and the covariates (age group, sex, material and social deprivations) were predictors. To estimate the performance of the multimorbidity measures in predicting each health outcome over and beyond that of the baseline covariates and to assess the impact of the LP on the prediction performance, we built 1080 logistic regression models for each combination of criterion used to define multimorbidity (3 criteria), list of diseases (3 lists), LP (20 periods) and health outcomes (6 outcomes). Of note, the analysis of polypharmacy and health services outcomes (hospitalisation, ED, GP, SP visits) included only those alive and covered by the drug plan during the entire one-year follow-up. Performance of each model was assessed using three measures: (1) the discrimination capacity of each model, that is the ability to identify correctly patients having the outcome within 1 year, with the *c-statistic* (also known as the area under the receiver operating characteristic curve) [23] (A difference in *c-statistic* superior to [0.010] was considered significant because covariates that contribute such difference may reduce confounding bias in observational studies [24]); (2) the overall performance of the model calculated with the scaled Brier score, which values range from 0 to 1 (higher value indicates better performance); and (3) the level of agreement between observed and predicted probability of the outcome using calibration intercept and slope, for which a value near zero and one indicates a better prediction, respectively [23].

All analyses were performed using SAS 9.4 (SAS Institute, Cary, NC).

### Supplementary and sensitivity analyses

Considering the recognized variations in claims history, risk of mortality and healthcare resource utilization associated with age and sex, we conducted stratified analyses to estimate the predictive performance according to these factors. We categorized age groups as 66–79 and ≥ 80 years, and also considered sex as a stratification factor. This approach allowed us to investigate the internal validity by assessing performance heterogeneity between these groups and is preferred to approaches that assess average performances (e.g., via bootstrapping), given the large size of the samples and the low complexity of the models [25].

We also repeated all the analyses using disease specific algorithms to take into account the shorter period of chronicity of some diseases. Because such algorithms are proposed in the literature only for all diseases included in the “Core list”(L20), we used them only for this list. Those algorithms are described in the supplementary material (SDC01: Table A1.3). For the “All-inclusive list”(L60) and the “Charlson & Elixhauser list”(L31), we limited the length of LP to 5 years for all mental health disorders having a remitting or relapsing course in these two lists [26, 27]. For the “Core list” (L20), we also re-ran all analyses by adding one supplementary disease (hypertension) to the list as hypertension is included in a majority of multimorbidity measures [3].

## Results

The study population included more than 1.4 million individuals older than 65 years (Table 1). The mean age was 75 years and 55% of individuals were female. Death occurred in 4% of the cohort. Other health outcomes were observed in proportions varying between 5% and 38%.

**Table 1:** Characteristics of the study cohort older than 65 on April 1st, 2019, Québec (Canada), *n* = 1,430,979

Characteristics		*n*	(%)
Age [Mean (SD)] (y)		75.4	(7.4)
Age group			
66–69		369,587	(25.8)
70–74		398,626	(27.9)
75–79		281,060	(19.6)
80–84		185,301	(12.9)
85–89		122,857	(8.6)
≥90		73,548	(5.1)
Sex			
Female		789,737	(55.2)
Male		641,242	(44.8)
Social Deprivation^a^			
First quintile – least deprived		236,925	(18.8)
Second quintile		253,655	(20.1)
Third quintile		254,309	(20.1)
Fourth quintile		262,728	(20.8)
Fifth quintile – most deprived		255,088	(20.2)
Material Deprivation^a^			
First quintile – least deprived		243,273	(19.3)
Second quintile		235,659	(18.7)
Third quintile		254,609	(20.2)
Fourth quintile		263,184	(20.8)
Fifth quintile – most deprived		265,980	(21.1)
Health outcome			
1-year mortality		54,022	(3.8)
Polypharmacy (≥ 10/y)^b^		462,957	(37.6)
Frequent visits to ED (≥ 3/y) ^b^		57,993	(4.7)
Frequent visits to GP (≥ 7/y) ^b^		113,384	(9.2)
Frequent visits to specialist physicians (≥ 10/y) ^b^		154,991	(12.6)
Hospitalisation (≥ 1/y) ^b^		147,469	(12.0)

*Abbreviation: ED: emergency department; GP: general practitioners; na: not applicable; SD: standard deviation; y: year*

*a*: *168,274 individuals in the main cohort have a missing value for this variable*

*b*: *The frequency of this health outcome was estimated in a subcohort (n = 1,231,656) excluding people without continuous public drug plan coverage from April 1st, 2019 to March 31, 2020 and deceased people between April 1st, 2019 to March 31th, 2020*

### Lookback and prevalence

**Fig. 2:**
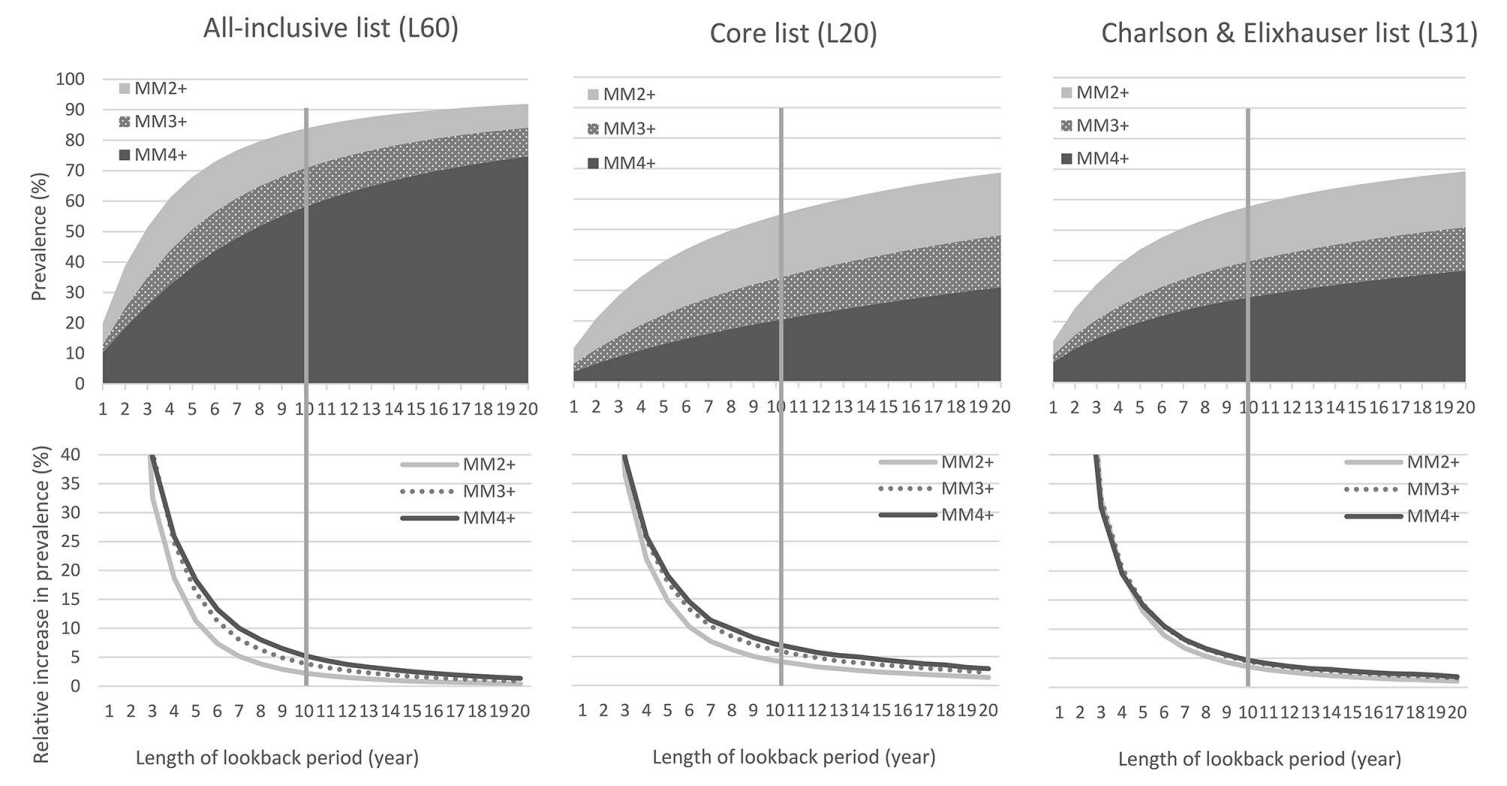
Impact of the length of lookback periods (1 to 20 years) on multimorbidity prevalence according to the type of multimorbidity definition (≥ 2 chronic conditions [MM2+], ≥ 3 chronic conditions [MM3+], ≥ 4 chronic conditions [MM4+]) and the list of diseases (the “All-inclusive” list (L60) grouped all ICD codes of chronic diseases into 60 chronic conditions; the “Core list” (L20) included 20 chronic diseases associated with a high Disable-adjusted life years (DALY) impact; the “Charlson & Elixhauser” list (L31) combined 31 medical conditions included in both indices). The vertical grey lines delineate the minimal lookback period (10 years) required to reach a more “stabilized” multimorbidity prevalence

The prevalence of multimorbidity increased with the length of the LP for each criterion used to define multimorbidity and for each list of diseases (Fig. 2). As expected, the prevalence of multimorbidity was higher for the “All-inclusive list”(L60) than for the “Core list”(L20). Using the MM2 + criterion, the multimorbidity prevalence was more than 1.5 times higher in the “All-inclusive list”(L60) (68% [5 years LP]; 84% [10 years LP]) than in the “Core list”(L20) (40% [5 years LP]; 55% [10 years LP]) (Fig. 2; SDC: Table A2.1). Prevalence estimates of the “Charlson & Elixhauser list”(L31) were quite similar to the “Core list”(L20). For each list of diseases and each criterion used to define multimorbidity, the multimorbidity prevalence increased when LP increased. More precisely, the prevalence increased more rapidly when the lookback period is less than 5 years, while the increase became less pronounced when the lookback period extends beyond 10 years. For example, for the “Core list” (L20), prevalence increased incrementally from 11% to 40%, 55%, 63%, and 69% as the LP increased from 1 to 5, 10, 15, and 20 years (Fig. 2; SDC: Table A2.1).

### Lookback and predictive performance

**Fig. 3:**
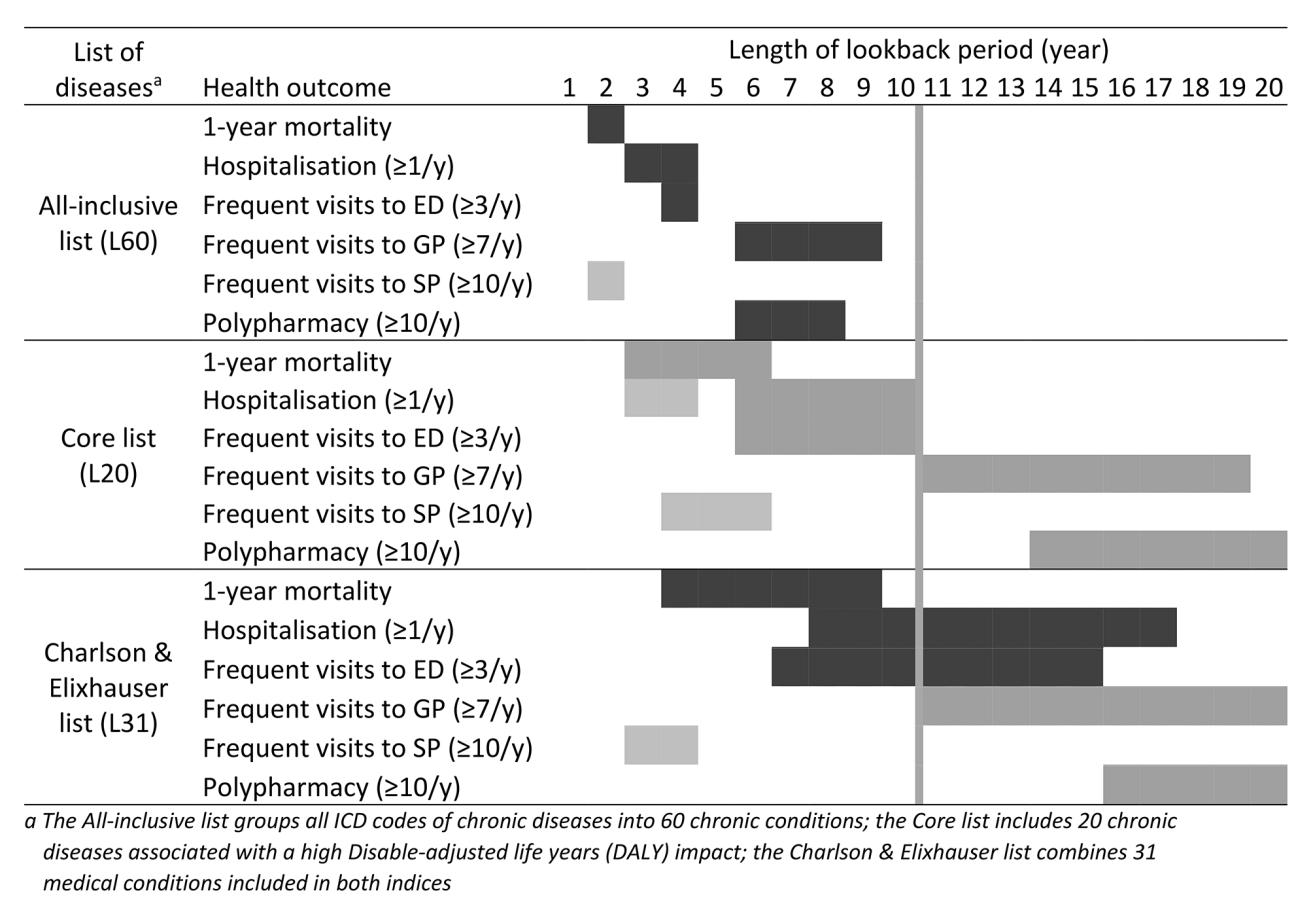
Illustration of the length of lookback periods where the predictive performance is maximal. Light-grey areas indicate maximal predictive performance for the ≥ 2 chronic conditions (MM2+) definition, grey-dot-pattern areas for the ≥ 3 chronic conditions (MM3+) definition, and dark-grey areas for the ≥ 4 chronic conditions (MM4+) definition. Shaded areas indicate the length of lookback period where the *c-statistic* ranged in the standard error interval [± 0.001] of the maximal *c-statistic*. The vertical grey line delineate the minimal lookback period (10 years) required to reach a more “stabilized” multimorbidity prevalence

The length of the LP for which a maximal performance in prediction was reached varied widely (Fig. 3). For example, the maximum performance for predicting mortality with the “All-inclusive list”(L60) was achieved with a LP of 2 years compared to 20 years for predicting polypharmacy with the “Core list” (L20). Globally, a shorter length of LP was required for the “All-inclusive list” (L60) than for the “Core list” (L20). In the “All-inclusive list” (L60), the maximal performance was reached with a LP < 5 years for all health outcomes except polypharmacy and frequent visits to the GP (LP = 7 years for both outcomes). In the “Core list” (L20), the maximal performance was reached when the LP varied between 5 and 10 years for all outcomes, except polypharmacy and frequent visits to the GP (LP > 10 years). Nonetheless, the maximum performance values were quite similar for both these two lists (Table 2). In both cases, the highest value of the *c-statistic* was observed with mortality (> 0.800). The performance of the “Charlson & Elixhauser list” (L31) was lower than the two other lists for frequent visits to GP or SP (L60 only), but higher for mortality and polypharmacy.

**Table 2:** Maximal predictive performance (*c-statistic*) value and length of the lookback period when it is reached (year) (> 65 years old as of April 1st, 2019, Québec [Canada], *n* > 1.4 million)

			*c-statistic*^b^ (length of LP when the maximal predictive performance is reached) by health outcomes					
List of diseases^a^		MM definition	1-year mortality	Hospitalisation (≥ 1/y)	Frequent visits to ED (≥ 3/y)	Frequent visits to GP (≥ 7/y)	Frequent visits to specialist physicians (≥ 10/y)	Polypharmacy (≥ 10/y)
All-inclusive list (L60)		MM2+	0.793 (1)	0.668 (1)	0.696 (1)	0.682 (3)	**0.695** (2)	0.710 (3)
		MM3+	0.799 (1)	0.676 (2)	0.708 (3)	0.689 (5)	0.692 (3)	0.723 (5)
		MM4+	**0.802** (2)	**0.679** (3)	**0.714** (4)	**0.693** (7)	0.691 (5)	**0.733** (7)
Core list (L20)		MM2+	0.800 (2)	0.676 (3)	0.710 (3)	0.696 (6)	**0.675** (5)	0.734 (7)
		MM3+	**0.801** (4)	**0.676** (7)	**0.714** (8)	**0.699** (14)	0.671 (12)	**0.737** (7)
		MM4+	0.798 (7)	0.672 (15)	0.713 (13)	0.695 (20)	0.663 (20)	0.728 (20)
Charlson & Elixhauser list (L31)		MM2+	0.809 (2)	0.677 (3)	0.711 (3)	0.678 (5)	**0.682** (4)	0.732 (8)
		MM3+	0.814 (4)	0.680 (5)	0.716 (5)	**0.681** (19)	0.677 (9)	**0.748** (8)
		MM4+	**0.815** (5)	**0.681** (11)	**0.718 (10)**	0.680 (20)	0.672 (19)	0.745 (20)

*Abbreviation: ED: emergency department; GP: general practitioners; LP: lookback period; MM2+: multimorbidity defines as ≥ 2 chronic conditions; MM3+: multimorbidity defined as ≥ 3 chronic conditions; MM4+: multimorbidity defined as ≥ 4 chronic conditions; y: year*

*a*: *The All-inclusive list groups all ICD codes of chronic diseases into 60 chronic conditions; the Core list includes 20 chronic diseases associated with a high Disable-adjusted life years (DALY) impact; the Charlson & Elixhauser list combines 31 medical conditions included in both indices.*

*b*: *The c-statistic value in bold indicate the maximal value observed and the length of the lookback period (year) when the maximal c-statistic value is met for a specific health outcome, for each list of diseases*

Prediction performance also varied according to the criterion used to define multimorbidity (Fig. 3). The maximum performance was generally observed for MM3 + or MM4+ (Table 2). Calibration intercept was close to zero and slope close to one for all models (results not shown).

When the prevalence of multimorbidity stabilized, that is between 5 and 10 years LP, the “Core list” (L20) performed better than the “All-inclusive list” (L60), except for frequent visits to SP (Table 3, SDC02: Tables A2.2.1-A2.4.6). For example, with a LP of 7 years, the *c-statistic* value for 1-year mortality prediction was 0.798 for the “Core list” (L20) and 0.788 for the “All-inclusive list” (L60) (Table 3). The “Charlson & Elixhauser list” (L31) had similar performance to the “Core list” (L20), but performed better at 1-year mortality prediction.

**Table 3:** Predictive performance (*c-statistic*) when a 7-year lookback period is selected (> 65 years old as of April 1st, 2019, Québec [Canada], *n* > 1.4 million)

				*c-statistic*^b^ by Health outcomes					
List of diseases^a^		MM definition	Prev. (%)	1-year mortality	Hospitalisation (≥ 1/y)	Frequent visit to ED (≥ 3/y)	Frequent visit to GP (≥ 7/y)	Frequent visit to specialist physicians (≥ 10/y)	Polypharmacy (≥ 10/y)
All-inclusive list (L60)		MM2+	76.7	0.768	0.642	0.667	0.667	0.636	0.687
		MM3+	61.0	0.779	0.659	0.688	0.685	0.670	0.717
		MM4+	47.9	**0.788**	**0.672**	**0.706**	**0.693**	**0.687**	**0.733**
Core list (L20)		MM2+	46.8	0.791	0.671	0.705	**0.695**	**0.672**	**0.734**
		MM3+	27.5	**0.798**	**0.676**	**0.713**	0.692	0.666	0.722
		MM4+	15.8	0.798	0.667	0.706	0.677	0.640	0.691
Charlson & Elixhauser list (L31)		MM2+	50.8	0.797	0.669	0.702	**0.678**	0.673	**0.732**
		MM3+	33.9	0.811	**0.680**	0.716	0.677	**0.676**	0.731
		MM4+	23.7	**0.815**	0.679	**0.717**	0.670	0.660	0.714

*Abbreviation: ED: emergency department; GP: general practitioners; MM2+: multimorbidity defined as ≥ 2 chronic conditions; MM3+: multimorbidity defined as ≥ 3 chronic conditions; MM4+: multimorbidity defined as ≥ 4 chronic conditions; Prev.: prevalence; y: year*

*a*: *The All-inclusive list groups all ICD codes of chronic diseases into 60 chronic conditions; the Core list includes 20 chronic diseases associated with a high Disable-adjusted life years (DALY) impact; the Charlson & Elixhauser list combines 31 medical conditions included in both indices.*

*b*: *The c-statistic value in bold indicates the maximal value observed for a specific health outcome, for each list of diseases*

### Supplementary and sensitivity analysis

The impact of the length of LP on predictive performance was homogeneous across lists of diseases among age and sex subpopulations. This indicates that variation in age or sex has low impact on the validity of the predictive models (SDC02: Figures A1.1, A1.2; Tables A3.1, A3.2).

Using a validated case definition for each disease in the “Core list”(L20) had virtually no impact on performance, but it led to a decrease in prevalence. Conversely, adding hypertension to this list had no impact on the performance but increased the prevalence (SDC02: Tables A2.1, A2.3.1-A2.3.6). Limiting the LP to 5 years for mental disorders had no impact on the main findings for the “Charlson & Elixhauser list”(L31) and the “All-inclusive list”(L60) (SDC02: Tables A2.1, A2.2.1-A2.2.6, A2.4.1-A2.4.6).

## Discussion

In this population-based study, we found that the LP impacted both multimorbidity prevalence estimates and health outcome prediction. As expected, the prevalence of multimorbidity increased with increasing LPs. The increased rate was similar among all lists of diseases and criteria used to define multimorbidity. Our results suggest that multimorbidity increases when LP increases and that underestimation in prevalence appears less pronounced after 10 years of LP. LPs required to achieve optimal performance varied across diseases lists, criteria used to define multimorbidity and health outcomes. Furthermore, the maximal performance was observed almost exclusively for MM3 + or MM4 + regardless of the list of diseases and for all outcomes.

### Implication

The threefold impact of LP, list of diseases, and criteria used to define multimorbidity on predictive performance may create potential dilemma if there is a need to both estimate multimorbidity prevalence and predict health outcomes. Indeed, if a LP < 5 years clearly underestimates the prevalence of multimorbidity, peak prediction performance for some list of diseases can be reached within the 1–5 years LP range. Fortunately, the “Core list” (L20) might resolve, at least partially, this dilemma as the maximal predictive performance for most outcomes was reached when the LP was higher than 5 years. Our study underscores that availability of data, and hence the possible LP length, might impact the choice of the list of diseases and/or the selection of criterion used to define multimorbidity. For example, if the database allows only for a short LP (e.g., 2 years), a more inclusive list of diseases or the MM2 + criterion might be worthwhile.

Better performance of MM3 + or MM4 + in predicting health outcomes suggests that in the population aged > 65 years, defining multimorbidity as the co-occurrence of at least 3 or at least 4 diseases would allow for better identification of a sub-populations at higher risk for health outcomes.

### Interpretation within the context of the literature

The rapidity with which the multimorbidity prevalence “stabilized” as LP increased was lower in our study than what was observed in a Danish Study with a population of a similar age [6]. In that study, the MM2 + prevalence increased from 51 to 52% when LP increase from 10 to 15 years compared to 55–63% with the “Core list”[L20]). Nevertheless, results of both the Danish study and ours suggested that at least 5 to 10 years of LP are needed to limit the underestimation of multimorbidity prevalence. A reduction in prevalence underestimation after 10 years of LP was also observed for the eight chronic conditions included in a cohort of HIV patients [8].

A change in predictive performance with increasing LPs was also observed in other studies [9, 10]. Among Canadian patients newly diagnosed with hypertension in the early 2000s, the *c-statistic* value for prediction of hospitalization increased from 0.756 to 0.768 when the LP increased from 6 to 12 months and then remained similar until the maximum LP of 3 years [9]. In an Australian cohort of patients hospitalized between 1990 and 1996, the *c-statistic* value for re-hospitalization prediction increased continuously from 0.640 to 0.656 when the LP increased from 1 to 5 years [10]. Interestingly, we observed a reduction in predictive performance beyond a certain LP for several outcomes in our study, and the reduction was more pronounced for the list with the largest number of chronic diseases (L60). For example, we observed a reduction of 0.023 in the *c-statistic* for mortality when LP increased from 2 years (*c-statistic* = 0.800) to 20 years (*c-statistic* = 0.777) with the L20 list, and 0.032 with the L60 list. These results could imply that, to some extent, diagnosis codes of prevalent and newly diagnosed conditions observed long in the past may have a limited impact on current health outcomes. Maximal predictive performance is reached with very short LP for some outcomes. Such performance might be attributed not only to the count of chronic conditions, but also to recent healthcare resource use. Indeed, short LPs are more likely to capture diagnoses from individuals who frequently utilize healthcare resources.

### Strengths and limitations

This is the first study to assess the impact of the LP on both multimorbidity prevalence and health outcome prediction in a general population setting. Selection bias was minimized as the data registry included almost the entire population over age 65 in the province of Québec, Canada. Another strength of the study is that we identified diseases using both outpatient and inpatient data and that we had data on health conditions retrospectively for more than 20 years. The use of either one of these datasets alone (inpatient or outpatient) would have underestimated both the prevalence and predictive performances [9]. We also included six health outcomes, allowing us to observe that the length of the LP required to maximize the predictive performance varied from 2 to 20 years. We also used a broad representativeness of disease lists used in administrative data. Nonetheless, generalization of our results to other lists used in multimorbidity measures should be made with caution. While multimorbidity measures based on the count of chronic diseases are useful when considering multiple health outcomes simultaneously, measures based on weighted indices like the Charlson Index or the Combined index (a combination of the Charlson and Elixhauser indices) might be more appropriate when focusing on a specific outcome. For instance, Kondalsamy-Chennakesavan et al. developed an adapted version of the Charlson index to predict surgical adverse events [28]. However, one drawback of such indices is that their weighting requires regular revision, as it can vary over time and depending on the population being studied [11].

## Conclusions

A LP of ten years allowed to limit the underestimation of multimorbidity prevalence in health administrative databases. The optimal predictive performance is often reached when LP is smaller than 10 years according to the outcome or the number of diseases included in the list of diseases. This dilemma of balancing reliable multimorbidity prevalence and optimal outcome prediction complicates the choice of the multimorbidity measure. The “Core list” (L20) may partially resolve this dilemma, as it demonstrated optimal prediction for many outcomes within the five to ten-year time frame. Moreover, in populations aged 65 years and older, multimorbidity defined as ≥ 3 or ≥ 4 chronic diseases should be preferred to the conventional ≥ 2 diseases, as outcome prediction is typically better for the former. Our results provide a comprehensive assessment that will allow users to select the optimal choices according to the availability of LP in their datasets. These findings will inform the elaboration of a more robust and consensus-based multimorbidity measure relying on health administrative databases.

### Electronic supplementary material

Below is the link to the electronic supplementary material.

Supplementary Material 1. **Additional file 1**. Schematic illustration of the study design; diseases and ICD codes of each list of diseases

Supplementary Material 2. **Additional file 2**. results of supplementary and sensitivity analysis

## Data Availability

The datasets generated and/or analyzed during the current study are not publicly available due to data confidentiality requirements from the QICDSS.

## Abbreviations

ICD: International Classification of Diseases
ICD-9-QC: International Classification of Diseases, 9th Revision, Quebec adaptation
ICD-10-CA: International Classification of Diseases, 10th Revision Canadian Coding Standard
L20: list of diseases including a core of 20 diseases
L31: list of diseases including 31 diseases from the Charlson and Elixhauser indices
L60: list of diseases grouping chronic conditions into 60 chronic diseases
LP: lookback period, that is the retrospective period of search for diagnosis codes in administrative data
MM2+: multimorbidity defined as the presence of at least 2 chronic diseases
MM3+: multimorbidity defined as the presence of at least 3 chronic diseases
MM4+: multimorbidity defined as the presence of at least 4 chronic diseases
RAMQ: Régie de l’assurance maladie du Québec
QICDSS: Québec Integrated Chronic Disease Surveillance System
