# Supplementary material for: Multimorbidity prevalence and health outcome prediction: assessing the impact of lookback periods, disease count, and definition criteria in health administrative data at the population-based level
Source: BMC Med Res Methodol. 2024 May 16;24:113. doi: 10.1186/s12874-024-02243-0 (PMC11097445; doi:10.1186/s12874-024-02243-0)
Supplement: Supplementary file 1 — Supplementary Material 1. Additional file 1. Schematic illustration of the study design; diseases and ICD codes of each list of diseases [file 12874_2024_2243_MOESM1_ESM.pdf]

Supplemental Digital Content 01

Table A1.1: diseases included in each list of diseases by category of diseases according to the International classification of diseases (ICD)

Table A.1.2: list of diseases and ICD-9 and ICD-10 codes of the All-inclusive list of diseases

Table A.1.3: list of diseases and ICD-9 and ICD-10 codes of the Selected minimal list of diseases. Information about case definitions only applied to the validated case definition used in the sensitivity analysis (For the main analysis, unless otherwise specified, we used the same ICD codes and case definition than the All-inclusive list [See Table A1.2])

Table A.1.4: list of diseases and ICD-9 and ICD-10 codes of the Charslon & Elixhauser list of diseases

Table A1.5: Characteristics of the entire cohort including all individuals aged over 65 years on April 1st, 2019 used for the prevalence estimation and mortality prediction and the subcohort excluding individuals not covered by the drug plan between April 1st, 2019 to March 31th,2020 used to predict polypharmacy and health services outcomes (hospitalisation, ED, GP and SP visits)

Table A1.1: diseases included in each list by category of diseases according to the International classification of diseases (ICD)

	Diseases		
Category of disease	All-inclusive list (60 diseases)	Core list (20 diseases)	Charlson & Elixhauser list (31 disease)
Cardiovascular and circulatory	Atrial fibrillation, Bradycardias and conduction diseases, Cardiac valve disease, Cerebrovascular disease, Heart failure, Hypertension, Ischemic heart diseases, Other cardiovascular diseases, Peripheral vascular diseases, Venous and lymphatic diseases	Coronary heart disease, Heart failure, Stroke	Cardiac arrhythmias, Cerebrovascular disease, Congestive heart failure, Hypertension, Myocardial infarction, Peripheral vascular disorders, Valvular disease
Congenital malformations	Chromosomal abnormalities	na	na
Endocrine	Diabetes, Dyslipidemia, Obesity, Other metabolic diseases, Thyroid diseases	Diabetes	Diabetes complicated, Diabetes uncomplicated, Fluid and electrolyte disorders, Hypothyroidism, Obesity, Weight loss
Gastrointestinal	Chronic liver disease, Chronic pancreas/biliary tract and gallbladder diseases, Colitis and related diseases, Esophagus/stomach and duodenum diseases, Inflammatory bowel diseases, Other digestive diseases	Chronic liver disease	Liver disease, Ulcer disease
Hematologic	Anemia, Blood and blood forming organ disease	na	Anemia, Coagulopathy
Immunologic	Allergy, Autoimmune diseases, Chronic infectious diseases	na	AIDS/HIV
Integumentary	na	na	na
Musculoskeletal	Dorsopathies, Inflammatory arthropathies, Osteoarthritis and other degenerative joint diseases, Osteoporosis, Other musculoskeletal and joint diseases	Chronic pain, Osteoarthritis, Osteoporosis[a], Musculoskeletal impairment due to injury	Rheumatoid arth./collagen vascular disease, Paralysis

Category of disease	Diseases<br>All-inclusive list (60 diseases)	Core list (20 diseases)	Charlson & Elixhauser list (31 disease)
Dermatosis	Chronic ulcer of the skin,<br>Other skin diseases	na	na
Nervous	Dementia,<br>Epilepsy,<br>Migraine and facial pain syndromes,<br>Multiple sclerosis,<br>Other neurological diseases,<br>Parkinson and parkinsonism,<br>Peripheral neuropathy	Dementia	Dementia,<br>Neurological disorders
Oncologic	Hematological neoplasm,<br>Solid neoplasm	Cancer	Any tumor without metastasis,<br>Metastatic cancer
Psychiatric	Depression and mood diseases,<br>Neurotic/stress-related and somatoform diseases,<br>Other Psychiatric and behavioral diseases,<br>Schizophrenia and delusional diseases,<br>Sleep disorders	Alcohol use disorder,<br>Depression and anxiety[b],<br>Drug use disorder,<br>Schizophrenia	Alcohol abuse,<br>Depression,<br>Drug abuse,<br>Psychoses
Pulmonary	Asthma,<br>COPD/emphysema/ chronic bronchitis,<br>Other respiratory diseases	Asthma, COPD	Chronic pulmonary disease,<br>Pulmonary circulation disorders
Renal	Chronic kidney diseases	Chronic renal disease	Renal disease
Reproductive (except cancer)	Prostate diseases, Other genitourinary diseases	Gynaecological disorder (Excluding urinary incontinence)	na
Senses diseases (oral, ear, eye)	Blindness/visual impairment,<br>Cataract and other lens diseases,<br>Deafness/hearing impairment,<br>Ear/nose/throat diseases,<br>Glaucoma,<br>Other eye diseases	Blindness/visual impairment,	na

Abbreviation: na: not applicable; COPD: chronic obstructive pulmonary disease

a Osteoporosis has been added to the original list of Ho et al. (2021) as osteoporosis is among the top 20 DALY for chronic conditions in Canada according to the Global Burden of Disease project (https://www.healthdata.org/gbd/2019)

b Depression and anxiety disorder have been grouped because of the precision of ICD code in our database.

Table A.1.2: list of diseases and ICD-9 and ICD-10 codes of the All-inclusive list of diseases (L60)

Category of disease	Diseases	ICD-9	ICD-10
Cardiovascular and circulatory	Atrial fibrillation	427.3	I48
	Bradycardias and conduction diseases	426.0, 426.10, 426.12, 426.13, 426.54, 426,6, V45.0<br>Only 4 digits<br>426.0*, 426.6, V45.0	I44.1, I44.2, I44.3, I45.3, I45.5, Z95.0
	Cardiac valve disease*	394.x–397.x, 424.x, 746.0[a], 746.1–746.6, V42.2, V43.3	I05.x-I08.x, I09.1, I09.8, I34.x-I38.x, I39.0-I39.4, Q22.x, Q23.x, Z95.2, Z95.3, Z95.4
	Cerebrovascular disease	362.34[a], 430.x-438.x	G45.x, G46.x, I60.x-I64.x, I67.x, I69.x
	Heart failure	402.01, 402.11, 402.91, 404.01, 404.03, 404.11, 404.13, 404.91, 404.93, 416.x, 417.0, 428.x, 429.1, 429.3, V42.1<br>Only 4 digits<br>402.1, 404.1, 416.x, 417.0, 428.x, 429.1, 429.3, 429.9, V42.1	I11.0[c], I13.0[c], I13.2[c], I27.x, I28.0, I42.x[c], I43.x[c], I50.x, I51.5, I51.7, I52.8, Z94.1, Z94.3
	Hypertension	401.x-405.x	I10.x-I15.x
	Ischemic heart diseases	410.x-414.x, V45.81[a], V45.82[a]	I20.x-I22.x, I24.x, I25.x, Z95.1, Z95.5
	Other cardiovascular diseases	393.x, 398.9, 417.1, 423.1, 423.2, 426.7, 426.81, 427.8, 440.0, 440.1, 440.3, 440.4[a], 440.8-x-442.x, 443.81[a], 458.0, 458.1, 458.8, 745.x, 746.8, 746.9, 747.x, V43.4<br>4 digits<br>393.x, 398.9, 417.1, 423.1, 423.2, 426.7, 427.8, 440.0, 440.1, 440.3, 440.4a, 440.8x-442.x, 458.0, 458.1, 458.8, 745.x, 746.8, 746.9, 747.x, V43.4	I09.0, I09.2, I09.9, I28.1, I31.0, I31.1, I45.6, I49.5, I49.8, I70.0, I70.1, I70.3-I70.9, I71.x, I72.x, I79.0, I79.1, I95.0, I95.1, I95.8, Q20.x, Q21.x, Q24.x-Q28.x, Z95.8, Z95.9
	Peripheral vascular diseases	440.2, 443.0, 443.9	I702, I73.0, I73.9, I79.2, I79.8
	Venous and lymphatic diseases	448.0, 454.x, 459.x, 457.x	I78.0, I83.x, I87.x, I89.x, I97.2, Q82.0
Congenital malformations	Chromosomal abnormalities	758.x	Q90.x-Q99.x
Endocrine	Diabetes	250.x,	E10.x-E14.x, E89.1[c]
	Dyslipidemia	272.0-272.5, 272.8,272.9	E78.x
	Obesity	278.0	E66.x
	Other metabolic diseases	040.2, 251.3, 252.x, 253.0-253.6, 253.8, 253.9, 255.x-257.x, 258.8-258.9, 259.2-259.3, 259.5-259.9, 260.x-263.x, 264.9, 267.x, 268.1, 268.2, 270.x-271.x, 272.6, 272.7, 273.4[a], 273.9, 275.x, 277.5, 277.8, 277.9, 330.0, 330.1, 579.3, 579.4, 579.8, 579.9, 588.x	E20.x-E29.x, E31.x, E34.x, E35[e], E35.1[d], E35.8[d], E40.x-E46.x, E64.x, E70.x-E72.x, E74.x-E77.x, E79.x, E80.x, E83.x-E85.x, E88.x, E89.x, K90.3, K90.4, K90.8, K90.9, K91.2, M83.x, M88.x, N25.x<br>Excluded: E23.1, E24.2, E24.4, E27.3, E34.3, E34.4, E35.0, E44.1, E79.0, E80.4, E88.3, E89.0, E89.2

Category of disease	Diseases	ICD-9	ICD-10
	Thyroid diseases	240.9, 242.x-244.x, 246.0, 246.1, 246.3-246.9	E00.x-E03.x, E05.x, E06.2, E06.3, E06.5, E07.x, E35.0[d], E89.0<br>Excluded: E03.5
Gastrointestinal	Chronic liver disease	070.22[a], 070.23[a], 070.2[b], 070.32[a], 070.33[a], 070.3[b], 070.44[a], 070.4[b], 070.54[a], 070.5[b], 571.1-571.6, 571.9, 572.3, 572.4, 572.8, 573.0, 573.3, V42.7	B18, K70.2-K70.9, K713, K714, K715, K717, K721, K73, K74, K753, K754, K758, K761, K766, K767, K778[d], Q446, Z944
	Chronic pancreas, biliary tract and gallbladder diseases	574.0-574.2, 575.11[a], 575.1[b], 577.1, 577.8, 577.9, 751.6	K80.0-K80.2, K80.8, K81.1, K86.0, K86.1, K86.8, Q44.0-Q44.5, Q45.0
	Colitis and related diseases	455.x, 557.1, 558.1[a], 558.4[a], 558.91[b], 562.1, 564.0, 564.1, 564.81[a], 569.1, 569.2, 569.43[a], 569.44[a], 569.49, 569.84, 569.85<br>Only 4 digits<br>455.x, 557.1, 558.1a, 558.4[a], 562.1, 564.0, 564.1, 569.1, 569.2	K52.0, K52.8, K55.1, K55.2, K57.2-K57.9, K58.x, K59.0, K59.2, K62.2-K62.4, K62.7-K62.9, K63.4, K64.0-K64.4, K64.8, K64.9
	Esophagus, stomach and duodenum diseases	456.0-456.2, 530.0, 530.1, 530.3, 530.5, 530.6, 530.85[a], 531.4-531.7, 533.4-533.7, 534.4-534.7, 535.1, 535.2, 535.4-535.6, 537.0-537.3, 537.7, 537.81, 750.3-750.8, V45.75[a]<br>Only 4 digits<br>456.0-456.2, 530.0, 530.1, 530.3, 530.5, 530.6, 530.85a, 531.4-531.7, 533.4-533.7, 534.4-534.7, 535.1, 535.2, 535.4-535.6, 537.0-537.3, 537.7, 750.3-750.8	I85, I86.4, I98.2, I98.3, K21.0, K22.0, K22.2, K22.4, K22.5, K22.7, K23.0[d], K23.1[d], K25.4-K25.7, K26.4-K26.7, K27.4-K27.7, K28.4-K28.7, K29.3-K29.9, K31.1-K31.5, Q39.x, Q40.x, Z90.3
	Inflammatory bowel diseases	555.x, 556.x	K50, K51
	Other digestive diseases	568.0, 579.0-579.2, 564.2, 567.7, 751.0-751.5, 787.6, v45.72[a], V45.3	B57.32e, K660, K900, K901, K902, K911, K93d, Q41-Q43, R15, Z904, Z980
Hematologic	Anemia	280.x-282.x, 283.0, 283.19a, 283.2, 283.9, 284.8, 285.0, 285.8, 285.9	D50-D53, D55, D56.1, D56.2, D56.4, D56.5, D56.8, D56.9, D57, D58, D591, D594, D595, D598, D599, D60.0, D60.8, D60.9, D61.0, D61.3-D61.9, D63, D64<br>Excluded: D642
	Blood and blood forming organ disease	135.x, 273.0, 279.0-279.3, 279.8, 279.9 286.0-286.4, 286.9, 287.0-287.3, 287.5-287.9, 288.1, 288.3, 288.4a, 289.4, 289.51[a], 289.6, 289.7, 289.89[a],	D66.x, D67.x, D68.0-D68.2, D68.8, D68.9, D69.0-D69.4, D69.6-D69.9, D71.x, D72.0, D73.0-D73.2, D74.0, D74.9, D75.0, D76.1, D76.3, D77.x, D80.0-D80.6, D80.8,D80.9, D81.x-D84.x, D86, D89.0, D89.2, D89.8, D89.9
Immunologic	Allergy	477.x, 493.0, 691.8, 708.0, V07.1	J30.1-J30.4, J45.0, K52.2, L20.x, L23.x, L50.0, Z51.6
	Autoimmune diseases	136.1, 443.1, 446.x, 694.2-694.5, 695.4, 696.0-696.2, 701.0, 710.0-710.4, 711.2, 713.6, 725.x, 729.30[a]	I73.1, L10.0-L10.4, L10.8, L10.9, L12.x, L40.x, L41.x, L93.x-L95.x, M30.x, M31.x, M32.1-M32.9, M33.x, M34.0, M34.1, M34.8, M34.9, M35.0-M35.6, M36.4-M36.8

Category of disease	Diseases	ICD-9	ICD-10
	Chronic infectious diseases	011.x-018.x, 030.x, 031.x, 042.x-044.x, 090.0, 093.x-097.x, 102.x, 103.x, 104.0, 114.4a, 120.x, 139.x, 730.1	A15.x-A19.x, A30.x, A31.x, A50.x, A52.x, A53.x, A65.x-A67.x, A69.2, A81.x, B20.x-B24.x, B38.1, B39.1, B40.1, B57.2-B57.5, B65.x, B92.x, B94.x, J65.x, M86.3-M86.6
Musculoskeletal	Dorsopathies	720.x-722.x, 723.0, 723.2, 723.3, 723.5, 723.8, 723.9, 724.6-724.9, 728.4, 732.0, 737.x, 738.4-738.6, 756.1	M40.x-M51.x, M53.x, Q67.5, Q76.1, Q76.4
	Inflammatory arthropathies	099.3, 274.x, 275.4, 711.1, 712.x, 713.0, 713.1, 713.3, 713.4, 713.7, 713.8, 714.x, 716.x, 719.2, 719.3, 720.0-720.2, 720.8, 720.9,	M02.3, M05.x-M14.x, M45.x, M46.0, M46.1, M46.8, M46.9
	Osteoarthritis and other degenerative joint diseases	715.x	M15.x-M19.x, M36.2, M36.3
	Osteoporosis	733.0, 733.1	M80.x-M82.x
	Other musculoskeletal and joint diseases	137.3, 717.x, 718.x, 722.8, 728.1, 728.5-728.7, 730.7, 731.2, 732.1, 732.2, 732,7, 732.8, 733.7-733.9, 734.x, 736.3-736.9, 737.11[a], 737.12[a], 737.22[a], 737.33[a], 739.x,754.1, 754.3-754.8, 755.2-755.9, 756.4, 756.5, 885.0, 885.1, 886.0, 886.1, 887.0-887.7, 895.0, 895.1, 896.0-896.2, 897.0-897.7, 929.9, 996.49[a], V43.6, V43.7, V42.4, V52.0, V52.1,	B90.2, M21.2-M21.9, M22.x-M24.x, M25.2, M25.3, M35.7, M61.x, M65.2, M65.3, M65.4, M70.0, M72.0, M72.2, M72.4, M75.0, M75.1, M75.3, M75.4, M79.7, M84.1, M89.x, M91.x, M93.x, M94.x, M96.x, M99.x, Q65.x, Q66.x, Q68.x, Q71.x-Q74.x, Q77.x, Q78.x, Q79.6, Q79.8, Q87.x, S38.2, S48.x, S58.x, S68.x, S78.x, S88.x, S98.x, T05.x, T09.6[d], T11.6[d], T13.6[d], T14.7[d], T90.x-T98.x[e], Z44.0, Z44.1, Z89.1-Z89.9, Z94.6, Z96.6, Z97.1
Dermatosis	Chronic ulcer of the skin	454.0, 454.2, 707.0, 707.1, 707.8	I83.0, I83.2, L89.x, L97.x, L98.4
	Other skin diseases	694.0, 694.1, 694.8, 694.9, 697.0, 698.2, 698.3, 705.81a, 706.8, 708.8	L13.x, L28.x, L30.1, L43.0, L43.1, L43.3, L43.8, L43.9, L50.8, L58.1, L85.x, Q80.x, Q81.x, Q82.1, Q82.2, Q82.9
Nervous	Dementia	290.x, 294.1, 331.0-331.2, 331.7-331.9	F00.x-F03.x, F05.1, G30.x, G31.x
	Epilepsy	345.0, 345.1, 345.4-345.6, 345.8, 345.9	G40.0-G40.4, G40.6-G40.9
	Migraine and facial pain syndromes	346.x, 350.x, 784.0	G43.x, G44.0-G44.3, G44.8, G50.x
	Multiple sclerosis	340.x	G35.x
	Other neurological diseases	137.1, 326.x, 330.2, 330.3, 331.3, 331.4, 331.7, 333.1, 333.2, 333.4-333.6, 333.8, 333.9, 334.0-334.4, 335.x-337.x, 341.1, 341.8, 341.9, 342.x, 343.x, 344.0-344.5, 344.81[a], 344.9, 348.8, 348.9, 350.0[b], 350.1, 351.1, 351.8, 351.9, 352.0-352.5, 352.9, 358.x, 359.x, 721.4, 740.x-742.x, 756.17[a], 758.0-758.2,	B90.0, D48.2, G04.1, G09.x-G12.x, G13.2, G13.8, G24, G25.0, G25.2, G25.3, G25.5, G25.8, G25.9, G26.x, G32.x, G37.x, G51.1-G53.x, G70.x, G71.x, G72.3, G72.4, G72.8, G72.9, G73.0, G73.1, G73.5-G73.7, G80.x-G82.x, G83.0-G83.5, G83.9, G90.x, G91.x, G93.8, G93.9, G95.x, G99.x, M47.1, Q00.x-Q07.x, Q76.0
	Parkinson and parkinsonism	332.x, 333.0	G20.x, G21.1-G21.9, G22.x, G23.x
	Peripheral neuropathy	138.x, 353.x-356.x, 357.1, 357.2, 357.4, 357.8, 723.4, 724.4, 729.2	B91.x, G14.x, G54.x-G60.x, G62.8, G62.9, G63.0, G63.2-G63.8, M47.2, M53.1, M54.1

Category of disease	Diseases	ICD-9	ICD-10
Oncologic	Hematological neoplasm	200.x-208.x, 236.8, 273.3,	C81.x-C86.x, C88.x, C90.x-C96.x
	Solid neoplasm	140.x-199.x, 230.x-234.x,	C00.x-C80.x, C97.x, D00.x-D09.x
sychiatric	Depression and mood diseases	296.x, 300.4, 308.x, 309.x, 311.x, 312.2b, 312.3a,	F30.x-F34.x, F38.x, F39.x, F41.2, F43.2
	Neurotic, stress-related and somatoform diseases	300.0-300.3, 300.5-300.8, 306.0-306.2	F40.x, F41.0, F41.1, F41.3-F41.9, F42.x, F43.1, F43.3-F43.9, F44.x, F45.x, F48.x
	Other Psychiatric and behavioral diseases	301.0, 301.2-301.9, 302.7, 303.x, 304.x, 307.1, 307.2, 307.5, 315.x, 317.x-319.x,	F04.x, F06.x, F07.x, F09.x, F10.2, F10.6[d], F10.7[d], F10.96[e], F11.2, F11.6[d], F11.7[d], F12.2, F126[d], F127[d], F132, F136[d], F137[d], F14.2, F14.6[d], F14.7d, F15.2, F15.6[d], F15.7[d], F16.2, F16.6[d], F16.7[d], F17.2, F17.6[d], F17.7[d], F18.2, F18.6[d], F18.7[d], F19.2, F19.6[d], F19.7[d], F50.x, F52.x, F60.x-F63.x, F68.x, F70.x-F73.x, F78.x-F84.x, F88.x, F89.x, F95.x, F99.x
	Schizophrenia and delusional diseases	295.x, 297.x, 298.3-298.8	F20.x, F22.x-F25.x, F28.x, F29.x
	Sleep disorders	347.x, 780.5	F51.0-F51.3, G47.x
Pulmonary	Asthma	493.0, 493.1, 493.3-493.9	J45.x
	COPD, emphysema, chronic bronchitis	491.x, 492.x, 493.2, 494.x	J41.x-J44.x, J47.x
	Other respiratory diseases	137.0, 278.03a, 495.x, 500.x-505.x, 506.4, 508.8, 511.0, 515.x, 516.0, 516.2-516.9, 518.1, 518.2, 518.8, 519.8, 519.9, 748.4-748.9, 799.1, v426	B90.9, E66.2, J60.x-J67.x, J68.4, J70.1, J70.3, J70.4, J84.x, J92.x, J94.1, J95.3, J95.5, J96.1, J98.0, J98.2-J98.9, Q33.x, Q34.x, Z90.2, Z94.2, Z94.3, Z96.3
Renal	Chronic kidney diseases	403.01, 403.11, 403.91, 404.x, 582.0, 582.1, 582.4, 582.8, 582.9, 583.0-583.2, 583.4, 583.8, 583.9, 585.x[b], 585.3-585.9[a], 590.0, 753.0, 753.1, V42.0<br>Only 4 digits<br>403.x, 404.x, 582.0, 582.1, 582.4, 582.8, 582.9, 583.0-583.2, 583.4, 583.8, 583.9, 585.x[b], 585.3-585.9[a], 590.0, 753.0, 753.1, V42.0	I120[c], I13.x[c], N01.x, N03.x-N05.x, N07.x, N08.x, N11.x, N18.x[b], N18.3-N18.9[c], Q60.x, Q61.1-Q61.9, Z90.5, Z94.0
Reproductive (except cancer)	Prostate diseases	600.x, 601.1, 601.8	N40.x, N41.1, N41.8
	Other genitourinary diseases	137.2, 592.0, 592.9, 594.0, 594.1, 594.8, 594.9, 595.1-595.3, 596.0, 596.3-596.5, 596.7-596.9, 598.0, 598.1, 598.8, 598.9, 614.1, 614.4. 614.6, 614.7, 615.1, 618.0-618.5, 618.7-618.9, 622.2-622.6, 622.8, 622.9, 623.2, 624.1, 627.3, 625.6, 752.6, 753.2, 753.5-753.9, 788.3,	B90.1, N20.0, N20.2, N20.9, N21.0, N21.8, N21.9, N22.x, N30.1-N30.4, N31.x, N32.0, N32.3, N32.8, N32.9, N33.x, N35.x, N39.3, N39.4, N48.0, N48.4, N48.9, N70.1, N71.1, N73.1, N73.4, N73.6, N76.1, N76.3, N81.x, N88.x, N89.5, N90.5, N95.2, Q54.x, Q62.0-Q62.4, Q62.7, Q62.8, Q63.8-Q64.1, Q64.3-Q64.9, Z90.6, Z90.7, Z96.0
Senses diseases (oral, ear, eye)	Blindness, visual impairment	369.x, V41.0, V43.0, V52.2,	H54.x, Z44.2, Z97.0
	Cataract and other lens diseases	366.x, 379.3, 743.3, V43.1	H25.x-H28.x, Q12.x, Z96.1

Category of disease	Diseases	ICD-9	ICD-10
	Deafness, hearing impairment	387.x, 389.0-389.2, 389.7, 389.9, 744.0, V53.2,	H80.x, H90.x, H91.1, H91.3, H91.9, Q16.x, Z45.3, Z46.1, Z96.2, Z97.4
	Ear, nose, throat diseases	380.21[a], 382.2, 382.3, 383.1, 383.3 384.1, 385.1, 385.3, 386.0, 386.4, 386.5, 470.x-474.x, 476.x, 478.3, 524.x, 527.0, 527.7, 821.1-821.3, 748.0-748.3, 750.0-750.2	H60.4, H66.1-H66.3, H70.1, H71.x, H73.1, H74.1, H81.0, H83.1, H83.2, H95.x, J30.0, J31.x-J33.x, J34.1-J34.3, J35.x, J37.x, J38.0, J38.6, K05.1, K05.3, K07.x[d], K11.0, K11.7, M26.x[e], Q30.x-Q38.x
	Glaucoma	365.x	H40.x
	Other eye diseases	361.x, 362.2, 362.5-362.9, 363.3-363.5, 363.8, 363.9, 364.1, 364.4-364.9, 371.0, 371.4-371.9, 372.1, 374.2, 375.0-375.2, 375.4-375.9, 376.1, 376.3-376.9, 377.1, 377.2, 377.4-377.9, 378.5, 378.8, 743.0-743.2, 743.4-743.9, V42.5	H02.2- H02.5, H04.x, H05.x, H10.4, H17.x, H18.4-H18.9, H19.3, H19.8, H20.1, H21.x, H31.0, H31.1, H31.2, H31.8, H31.9, H33.x, H35.2, H35.3, H35.4, H35.5, H35.7-H35.9, H36, H47.x-H49.x, H51.x, Q10.x, Q11.x, Q13.x-Q15.x, Z94.7<br>Excluded: H043, H050, H470, H471, H481

Abbreviations: CA: Canadian version; CM: clinical modification version; COPD: chronic obstructive pulmonary disease; ICD-9: International classification of diseases 9th edition; ICD-10: International classification of diseases 10th edition; QC: Quebec adaptation

a Included in ICD-9-CM only

b Included in ICD-9-QC only

c To exclude code to be equivalent to ICD-9-QC

d Included in ICD-10-CA only

e Included in ICD-10-CM only

NOTE: The original list of ICD-10 codes and the methodology used to select these codes is available in:
Calderon-Larranaga A, Vetrano DL, Onder G, et al. Assessing and Measuring Chronic Multimorbidity in the Older Population: A Proposal for Its Operationalization. The journals of gerontology Series A, Biological sciences and medical sciences. 2017;72(10):1417-1423
The methodology used to translate ICD-10 codes version into the ICD-9 version is described in :
Simard M, Sirois C, Candas B. Validation of the Combined Comorbidity Index of Charlson and Elixhauser to Predict 30-Day Mortality Across ICD-9 and ICD-10. Med Care. 2018;56(5):441-447

Table A.1.3: list of diseases and ICD-9 and ICD-10 codes of the Core list of diseases. Information about case definitions only applied to the validated case definition used in the sensitivity analysis (For the main analysis, unless otherwise specified, we used the same ICD codes and case definition than the All-inclusive list [See Table A1.2])

Category of disease	Diseases	ICD-9	ICD-10	Case definition	Maximum lookback period	Validation	Accuracy
Cardiovascular and circulatory	Coronary heart disease	410.x-414.x	I20.x-I25.x	1 H[h] or 2 P in 1 Y	None	Chart review	Sens: 72%<br>Spec: 95%<br>PPV: 87%<br>NPV: 88%<br>Tu, 2007<br><br>Sens: 75%<br>Spec: 94%<br>PPV: 81%<br>NPV: 92%<br>Quan, 2009
	Heart failure	428.x	I50.x	1 H or 2 P in 1 Y [≥40 years old]	None	Chart review	Sens: 85%<br>SPEC: 97%<br>PPV: 56%<br>Schultz, 2013
	Hypertension	401.x-405.x	I10.x-I15.x	1 H or 2 P in 2 Y [pregnancy exclusion[i]]	None	Chart review	Sens: 72%<br>Spec: 95%<br>PPV: 87%<br>NPV: 88%<br>Tu, 2007<br><br>Sens: 75%<br>Spec: 94%<br>PPV: 81%<br>NPV: 92%<br>Quan, 2009
	Stroke	Hospitalization: 362.3, 430.x, 431.x, 433.x1, 434.x1, 435.x,436.x<br>Physician claim: 430.x, 431.x, 434.x-436.x	G45.0-G45.3, G45.8, G45.9, H34.0, H34.1, I60.x, I61.x, I63.0-I63.5, I63.8, I63.9, I64.x	1 H or 2 P in 1 Y [≥20 years old]	None	Chart review	Sens: 68%<br>Spec: 99%<br>PPV: 66%<br>NPV: 99%<br>Tu, 2013
Endocrine	Diabetes	250.x	E10.x-E14.x	1 H or 2 P in 2 Y [pregnancy exclusion]	None	Chart review	Sens: 94%<br>Spec: 99%<br>PPV: 82%<br>NPV: 99%<br>Dart, 2011
Gastrointestinal	Chronic liver disease	070.22[a], 070.23[a], 070.2[b], 070.32[a], 070.33[a], 070.3[b], 070.44[a], 070.4[b], 070.54[a], 070.5[b], 456.0–456.2, 571.x, 572.3, 572.8, 573.3, 573.4, 573.9, V42.7	B18.x, I85.x, I86.4, I98.2, K70.0- K70.3, K70.9, K71.1, K71.3–K71.5, K71.6, K71.7, K72.1, K72.9, K73.x, K74.x, K75.4, K76.0, K76.1, K76.3, K76.4, K76.5, K76.6, K76.8, K76.9, Z94.4	1 H or 2 P in 2 Y, ≥30 days apart	None	Chart review	Sens: 38-41%<br>Spec: 99%<br>PPV: 80-85%<br>NPV: 97%<br>Quan, 2008

Category of disease	Diseases	ICD-9	ICD-10	Case definition	Maximum lookback period	Validation	Accuracy
Musculoskeletal	Chronic pain[f]	307.80, 307.89, 338.0[a], 338.2[a], 338.4[a], 719.41[a], 719.45[a]-719.47[a], 719.49[a], 719.4[b], 720.0, 720.2, 720.9, 721.0 -721.4, 721.6, 721.8, 721.9, 722, 723.0, 723.1, 723.3 -723.9, 724.0 - 724.6, 724.70[a], 724.79[a], 724.7[b], 724.8, 724.9, 729.0 - 729.2, 729.4, 729.5<br>4 digits: 719.4, 720.0, 720.2, 720.9, 721.0 - 721.4, 721.6, 721.8, 721.9, 722, 723.0, 723.1, 723.3 -723.9, 724.x, 729.0 - 729.2, 729.4, 729.5	F45.4, G89.0[e], G89.2[e], G89.4[e], M08.1, M25.50, M25.51, M25.55 - M25.57, M43.2 - M43.6, M45, M46.1, M46.3, M46.4, M46.9, M47, M48.0, M48.1, M48.8, M48.9, M50.8, M50.9, M51, M53.1 - M53.3, M53.8, M53.9, M54, M60.8, M60.9, M63.3, M79.0 - M79.2, M79.6, M79.7, M96.1	2 H or 2P in 1Y, ≥30 days apart	2 Y	Chart review	Sens: 71%Spec: 99%PPV: 95%NPV: 93% Tian, 2013
	Osteoarthritis	715.x	M15.x-M19.x	1 H or 2 P in 2 Y	None	Chart review	Sens: 21-26% Spec: 91-100% PPV: 85-100% NPV: 9-37% Rahman,2016
	Osteoporosis[g]	733.0, 733.9	M80.x, M81.x	1 H or 1 Rx[j] or 2P in 2Y	None	Chart review	Sens: 86% Spec: 93% PPV: 91% NPV: 85% Leslie, 2011
	Musculoskeletal impairment due to injury[f]	334.1, 342.x, 343.x, 344.0, 344.1, 344.2, 344.3, 344.4, 344.5, 344.6, 344.8, 344.9	G04.1, G11.4, G80.x, G81.x, G82.x, G83.x	1 H or 2 P in 2 Y, ≥30 days apart	None	Chart review	Sens: 44-53% Spec: 99% PPV: 59-63% NPV: 97-98% Quan, 2008
Nervous	Dementia	Hospitalisation: 290, 294, 331.0, 331.1, 331.5<br>Physician claim: 046.1, 290, 294, 331	G30.x, F00.x-F03.x	1 H or 3P in 2Y ≥30 days apart [≥40 years old] OR 1 Rx[k] [≥65 years old]	None	Chart review	Sens: 80% Spec: 99% PPV: 80% NPV: 99% Jaakkimainen, 2016
Oncologic	Cancer	140.x-172.x, 174.x-199.x	C00.x–C43.x, C45.x–C80.x	1 H or 2 P in 2 Y, ≥30 days apart	5 Y	Chart review	Sens: 43-87% Spec: 97-99% PPV: 57-63% NPV: 95-99% Quan, 2008
Psychiatric	Alcohol use disorder[f]	265.2, 291.1–291.3, 291.5–291.9, 303.x, 305.0, 357.5, 425.5, 535.3, 571.0–571.3, 980.x, V11.3a	F10.x, E52.x, G62.1, I42.6, K29.2, K70.0, K70.3, K70.9, T51.x, Z50.2, Z71.4, Z72.1	1 H or 2 P in 2 Y, ≥30 days apart	None	Chart review	Sens: 52-54% Spec: 99% PPV: 83-84% NPV: 96% Quan, 2008

Category of disease	Diseases	ICD-9	ICD-10	Case definition	Maximum lookback period	Validation	Accuracy
	Depression and anxiety[f]	296.x, 300.x, 311.x	F30.x-F48.x, F68.x	1 H or 2 P in 2 Y, ≥30 days apart	5 Y	Construct validity<br><br>Kisely, 2009	na
	Drug use disorder[f]	292.x, 304.x, 305.2–305.9, V65.4	F11.x–F16.x, F18.x-F19.x, Z71.5, Z72.2	1 H or 2 P in 2 Y, ≥30 days apart	None	Chart review	Sens: 47-55%Spec: 99%PPV: 74-81%NPV: 98% Quan, 2008
	Schizophrenia	295.x	F20.x, F21.x, F23.2, F25.x	1 H or 2 P in 2 Y, ≥30 days apart	None	Chart Review	Sens:92% Spec: 61% PPV: 67% NPV: 89% Kurdyak, 2015
Pulmonary	Asthma	493.x	J45.x-J46.x	1 H or 2 P in 2 Y [≥20 years old]	None	Chart review	Sens: 84% Spec: 77% PPV: 62% NPV: 91% Gershon, 2009a
	COPD	491.x, 492.x, 496.x	J41.x-J44.x	1 H or 3 P in 2 Y [≥35 ans]	None	Chart review	Sens: 58% Spec: 95% PPV: 81% NPV: 87% Gershon, 2009b
Renal	Chronic renal disease	403.01, 403.11, 403.91, 404.02, 404.03, 404.12, 404.13, 404.92, 404.93, 585.x, 586.x, 588.0, V42.0, V45.1, V56.x<br><br>Only 4 digits 403.0, 403.1, 403.9, 404.0, 404.1, 404.9, 585.x, 586.x, 588.0, V42.0, V45.1, V56.x	I12.0[c], I13.1[c], N18.x, N19.x, N25.0, Z49.x, Z94.0, Z99.2	1 H or 2 P in 2 Y, ≥30 days apart	None	Chart review	Sens: 79-81% Spec: 99% PPV: 64-71% NPV: 99% Quan, 2008

Category of disease	Diseases	ICD-9	ICD-10	Case definition	Maximum lookback period	Validation	Accuracy
Reproductive (except cancer)	Gynaecological disorder (Excluding urinary incontinence)[f]	137.2, 592.0, 592.9, 594.0, 594.1, 594.8, 594.9, 595.1-595.3, 596.0, 596.3-596.5, 596.7-596.9, 598.0, 598.1, 598.8, 598.9, 614.1, 614.4. 614.6, 614.7, 615.1, 618.0-618.5, 618.7-618.9, 622.2-622.6, 622.8, 622.9, 623.2, 624.1, 627.3, 625.6, 752.6, 753.2, 753.5-753.9	B90.1, N20.0, N20.2, N20.9, N21.0, N21.8, N21.9, N22.x, N30.1-N30.4, N31.x, N32.0, N32.3, N32.8, N32.9, N33.x, N35, N48.0, N48.4, N48.9, N70.1, N71.1, N73.1, N73.4, N73.6, N76.1, N76.3, N81.x, N88.x, N89.5, N90.5, N95.2, Q54.x, Q62.0-Q62.4, Q62.7, Q62.8, Q63.8-Q64.1, Q64.3-Q64.9, Z90.6, Z90.7, Z96.0	1 H or 2 P in 2 Y, ≥30 days apart	None	Construct validity<br><br>Calderon, 2017	na
Senses diseases (oral, ear, eye)	Blindness/vision impairment	369.x, V41.0, V43.0, V52.2	H54.x, Z44.2, Z97.0	1 H or 2 P in 2 Y, ≥30 days apart	None	Construct validity<br><br>Calderon, 2017	na

Abbreviations: CA: Canadian version; CM: clinical modification version; CCADTC: Canadienne Classification of Diagnostic, Therapeutic, and Surgical Procedures (CCP) which complements ICD-9 in the hospitalization file; CCI: Canadienne Classification of Health Interventions en santé which complements ICD-10 in the hospitalization file; COPD : Chronic obstructive pulmonary disease; H: Hospitalization; ICD-9: International classification of diseases 9th edition; ICD-10: International classification of diseases 10th edition; na: not applicable; NPV: Negative predictive value; P: Physician claim; PPV: Positive predictive value; QC: Quebec adaptation; Rx: drug prescription; Sens: Sensitivity; Spec: Specificity; Y: year

a Included in ICD-9-CM only

b Included in ICD-9-QC only

c To exclude code to be equivalent to ICD-9-QC

d Included in ICD-10-CA only

e Included in ICD-10-CM only

f Same ICD codes used in the original and sensitivity analysis

g Medical condition added to the original list of Ho et al. (2021) as osteoporosis is among the top 20 DALY for chronic conditions in Canada according to the Global Burden of Disease project (https://www.healthdata.org/gbd/2019)

h Included intervention code CCADTC (48.02, 48.03, 48.11-48.19) and CCI (1.IJ.50, 1.IJ.57.GQ, 1.IJ.54, 1.IJ.76)

i We excluded any ICD code for diabetes or hypertension in the period covering 120 days before and 180 days after an obstetrical hospitalization OR within 180 days of a physician claim procedure for prenatal monitoring, ultrasound or pregnancy management for female aged between 10 to 54 years old.

j List of drugs for osteoporosis are available at: Public health agency of Canada. OSTEOPOROSIS and RELATED FRACTURES in Canada: Report from the Canadian Chronic Disease Surveillance System 2020. Public Health Agency of Canada; 2020:85. https://publications.gc.ca/collections/collection_2021/aspc-phac/HP35-123-2020-eng.pdf

k Acetylcholinesterase inhibitors (AChEIs) and memantine were also used to identify individuals with MNCD. Anatomical Therapeutic Chemical Classification System (ATC) codes include: donepezil (DA02), rivastigmine (DA03), galantamine (DA04), and memantine (DX01).

References

Calderon-Larranaga A, Vetrano DL, Onder G, et al. Assessing and Measuring Chronic Multimorbidity in the Older Population: A Proposal for Its Operationalization. The journals of gerontology Series A, Biological sciences and medical sciences. 2017;72(10):1417-1423

Dart A. B. Martens P.J. Sellers E.A. Browneli M.D. Rigatto C.R. Dean H.J., Validation of a pediatric diabetes case definition using administrative health data in Manitoba, Canada. Diabetes Care 2011; 34(4): 898-903

Gershon A.S. Wang C. Guan J. Vasilevsk-Ritovska J. Cicutto L. To T., Identifying patients with physician-diagnosed asthma in health administrative databases. Can Respir Journal 2009a; 16(6): 183-188

Gershon AS, Wang C, Guan J, Vasilevska-Ristovska J, Cicutto L, To T. Identifying individuals with physcian diagnosed COPD in health administrative databases. COPD. 2009b;6(5):388-394

Hux J.E. Flintoft V. Ivis F. Bica A., Determination of prevalence and incidence using a validated administrative data algorithm. Diabetes care 2002; 25(3): 512-516

Jaakkimainen RL, Bronskill SE, Tierney MC, et al. Identification of Physician-Diagnosed Alzheimer's Disease and Related Dementias in Population-Based Administrative Data: A Validation Study Using Family Physicians' Electronic Medical Records. J Alzheimers Dis. 2016;54(1):337-349

Kisely S, Lin E, Gilbert C, Smith M, Campbell LA, Vasiliadis HM. Use of administrative data for the surveillance of mood and anxiety disorders. Aust N Z J Psychiatry. 2009;43(12):1118-1125

Kurdyak P, Lin E, Green D, Vigod S. Validation of a Population-Based Algorithm to Detect Chronic Psychotic Illness. Canadian journal of psychiatry Revue canadienne de psychiatrie 2015;60:362-8

Leslie, W.D., Lix, L.M., Yogendran, M.S. (2011) Validation of a case definition for osteoporosis disease surveillance. Osteoporos Int J Establ Reseult Coop Eur Found USA, 22: 37-46.

Quan H, Li B, Saunders LD, et al. Assessing validity of ICD-9-CM and ICD-10 administrative data in recording clinical conditions in a unique dually coded database. Health Serv Res 2008; 43: 1424–1441

Quan H. Khan N. Hemmelgarn B.R. Tu K. Chen G. Campbell N. Hill M.D. Ghali W.A. McAlister F.A., Validation of a case definition to define hypertension using administrative data. Hypertension 2009; 54: 1423-1428

Rahman MM, Kopec JA, Goldsmith CH, Anis AH, Cibere J. Validation of Administrative Osteoarthritis Diagnosis Using a Clinical and Radiological Population-Based Cohort. Int J Rheumatol. 2016;2016:6475318

Tian TY, Zlateva I, Anderson DR. Using electronic health records data to identify patients with chronic pain in a primary care setting. J Am Med Inform Assoc. 2013;20(e2):e275-e280

Tu K. Campbell N.RC. Chen Z. Cauch-Dudek K.J. McAlister F.A., Accuracy of administrative databases in identifying patients with hypertension, Open Medicine 2007; 1(1): e18-e26

Tu K. Wang M. Young J. Green D. Ivers N.M. Butt D. Jaakkimainen L. Kapral M.K., Validity of administrative data for identifying patients who have had a stroke or transient ischemic attach using EMRALD as a reference standard, Can J Cardio 2013; 29(11): 1388-94

Table A.1.4: list of diseases and ICD-9 and ICD-10 codes of the Charslon & Elixhauser list (L31)

Category of disease	Diseases	ICD-9	ICD-10
Cardiovascular and circulatory	Cardiac arrhythmias	426.0, 426.10, 426.12, 426.13, 426.7, 426.9, 427.0–427.4, 427.6–427.9, 785.0, 996.01, 996.04, V45.0, V53.3<br><br>Only 4 digits<br>426.0, 426.7, 426.9, 427.0–427.4, 427.6–427.9, 785.0, V45.0, V53.3	I44.1–I44.3, I45.6, I45.9, I47.x–I49.x, R00.0, R00.1, R00.8, T82.1, Z45.0, Z95.0
	Cerebrovascular disease	362.34[a], 430.x-438.x	G45.x, G46.x, I60.x-I69.x
	Congestive heart failure	398.91, 402.01, 402.11, 402.91, 404.01, 404.03, 404.11, 404.13, 404.91, 404.93, 428.x<br><br>Only 4 digits<br>402.1, 404.1, 428.x	I09.9[c], I11.0[c], I13.0c, I13.2[c], I25.5[c], I42.0[c], I42.5–I42.9c, I43.xc, I50.x, P29.0c
	Hypertension	401.x, 402.x–405.x, 437.2	I10.x, I11.x–I13.x, I15.x, I67.4
	Myocardial infarction	410.x, 412.x	I21.x, I22.x, I25.2
	Peripheral vascular disorders	093.x, 440.x, 441.x, 443.1– 443.9, 447.1, 557.1, 557.9, V43.4	A52.0, I70.x, I71.x, I72.x, I73.0, I73.1, I73.8, I73.9, I77.1, I79.0, K55.1, K55.8, K55.9, Z95.8, Z95.9
	Valvular disease	394.x–397.x, 424.x, 746.3–746.6, V42.2, V43.3	I05.x–I08.x, I09.1, I09.8, I34.x–I39.x, Q23.0–Q23.3, Q23.8, Q23.9, Z95.2, Z95.3, Z95.4
Endocrine	Diabetes, complicated	250.3[b], 250.4-250.9	E10.2-E10.8, E11.2-E11.8, E13.2-E13.8, E14.2-E14.8
	Diabetes, uncomplicated	250.0-250.2, 250.3[a]	E10.0, E10.1, E10.9, E11.0, E11.1, E11.9, E13.0, E13.1, E13.9, E14.0, E14.1, E14.9
	Fluid and electrolyte disorders	253.6, 276.x	E22.2, E86.x, E87.x
	Hypothyroidism	240.9, 243.x, 244.x, 246.1, 246.8	E00.x, E01.x, E02.x, E03.x, E89.0
	Obesity	278.0	E66.x
	Weight loss	260.x–263.x, 783.2, 799.4	E40.x–E46.x, R63.4, R64.x
Gastrointestinal	Liver disease	070.22[a], 070.23[a], 070.2[b], 070.32[a], 070.33[a], 070.3[b], 070.44[a], 070.4[b], 070.54[a], 070.5[b], 456.0–456.2, 571.x, 572.3, 572.8, 573.3, 573.4, 573.9, V42.7	B18.x, I85.x, I86.4, I98.2, K70.0- K70.3, K70.9, K71.1, K71.3–K71.5, K71.6, K71.7, K72.1, K72.9, K73.x, K74.x, K75.4, K76.0, K76.1, K76.3, K76.4, K76.5, K76.6, K76.8, K76.9, Z94.4
	Ulcer disease	531.x-534.x	K25.x-K28.x
Hematologic	Anemia<br>(Blood loss anemia, Deficiency anemia)	280.0[a], 280.90[b], 280.1–280.9[a], 280.91–280.99[b] 281.x, 285.9	D50.0, D50.1, D50.8, D50.9, D51.x–D53.x, D63.x, D64.9

Category of disease	Diseases	ICD-9	ICD-10
	Coagulopathy	286.x, 287.1, 287.3–287.5	D65.x–D68.x, D69.1, D69.3–D69.6
Immunologic	AIDS/HIV	042.x–044.x	B20.x-B24.x
Musculoskeletal	Paralysis	334.1, 342.x, 343.x,	G04.1, G11.4, G80.x-G83.x
	Rheumatoid arth./collagen vascular disease	344.0, 344.1, 344.2, 344.3, 344.4, 344.5, 344.6, 344.8, 344.9	L90.0, L94.0, L94.1, L94.3, M05.x, M06.x, M08.x, M12.0, M12.3, M30.x, M31.x, M32.x–M35.x, M45.x, M46.0, M46.1, M46.8, M46.9x
Nervous	Dementia	290.x, 294.1, 331.0, 331.2	F00.x-F03.x, F05.1, G30.x, G31.1
	Neurological disorders	331.9, 332.0, 332.1, 333.4, 333.5, 333.92[a], 334.x–335.x, 336.2, 340.x, 341.x, 345.x, 348.1, 348.3, 780.3, 784.3	G10.x–G12.x, G13.x, G20.x, G21.x–G22.x, G25.4, G25.5, G31.2, G31.8, G31.9, G32.x, G35.x, G36.x, G37.x, G40.x, G41.x, G93.1, G93.4, R47.0, R56.x
Oncologic	Any tumor without metastasis	140.x-172.x, 174.x, 175.x, 179.x-195.x, 200.x, 201.x, 202.x, 203.0, 238.6, 273.3	C00.x–C26.x, C30.x–C34.x, C37.x–C41.x, C43.x, C45.x-C58.x, C60.x–C76.x, C81.x-C85.x, C88.x, C90.0, C90.2, C96.x
	Metastatic cancer	196.x–199.x	C77.x-C79.x, C80.x
Psychiatric	Alcohol abuse	265.2, 291.1–291.3, 291.5–291.9, 303.x, 305.0, 357.5, 425.5, 535.3, 571.0–571.3, 980.x, V11.3[a]	F10.x, E52.x, G62.1, I42.6, K29.2, K70.0, K70.3, K70.9, T51.x, Z50.2, Z71.4, Z72.1
	Depression	296.1[b], 296.2[a], 296.3, 296.5[a], 300.4, 309.x, 311.x	F20.4, F31.3–F31.5, F32.x, F33.x, F34.1, F41.2, F43.2
	Drug abuse	292.x, 304.x, 305.2–305.9, V65.4	F11.x–F16.x, F18.x-F19.x, Z71.5, Z72.2
	Psychoses	293.8, 295.x, 296.04[a], 296.14[a], 296.44[a], 296.54[a], 297.x, 298.x, 299.1[b]	F20.x, F22.x–F25.x, F28.x, F29.x, F30.2, F31.2, F31.5
Pulmonary	Chronic pulmonary disease	490x–505.x, 506.4, 508.1, 508.8[a]	I27.8[c], I27.9[c], J40.x-J47.x, J60.x-J64.x, J65.x, J66.x, J67.x, J68.4, J70.1, J70.3
	Pulmonary circulation disorders	415.0, 415.1, 416.x, 417.0, 417.8, 417.9	I26.x, I27.x, I28.0, I28.8, I28.9
Renal	Renal disease	403.01, 403.11, 403.91, 404.02, 404.03, 404.12, 404.13, 404.92, 404.93, 585.x, 586.x, 588.0, V42.0, V45.1, V56.x<br><br>Only 4 digits<br>403.0, 403.1, 403.9, 404.0, 404.1, 404.9, 585.x, 586.x, 588.0, V42.0, V45.1, V56.x	I12.0[c], I13.1[c], N18.x, N19.x, N25.0, Z49.x, Z94.0, Z99.2

Abbreviations: CA: Canadian version; CM: clinical modification version; ICD-9: International classification of diseases 9th edition; ICD-10: International classification of diseases 10th edition; QC: Quebec adaptation

a Included in ICD-9-CM only

b Included in ICD-9-QC only

c To exclude code to be equivalent to ICD-9-QC

NOTE: The original list of ICD codes and the methodology used to select those codes is available in:
Simard M, Sirois C, Candas B. Validation of the Combined Comorbidity Index of Charlson and Elixhauser to Predict 30-Day Mortality Across ICD-9 and ICD-10. Med Care. 2018;56(5):441-447

Table A1.5: Characteristics of the entire cohort including all individuals ages over 65 years on April 1st, 2019 used for the prevalence estimation and mortality prediction and of the subcohort excluding individuals dead or not covered by the drug plan between April 1st, 2019 to March 31th,2020 used to predict polypharmacy and health services outcomes (hospitalisation, ED, GP and SP visits)

	Main cohort[b] (n = 1,430,979)		Subcohort [c] (n = 1,231,656)	
Characteristics	n	(%)	n	(%)
Age [Mean (SD) (y)	75.4	(7.4)	75.0	(7.0)
Age group				
66-69	369,587	(25.8)	321,465	(26.1)
70-74	398,626	(27.9)	356,421	(28.9)
75-79	281,060	(19.6)	249,418	(20.3)
80-84	185,301	(12.9)	158,611	(12.9)
85-89	122,857	(8.6)	97,595	(7.9)
≥90	73,548	(5.1)	48,146	(3.9)
Sex				
Female	789,737	(55.2)	685,108	(55.6)
Male	641,242	(44.8)	546,548	(44.4)
Social Deprivation[a]				
First quintile – least deprived	236,925	(18.8)	206,269	(18.7)
Second quintile	253,655	(20.1)	221,536	(20.1)
Third quintile	254,309	(20.1)	222,062	(20.2)
Fourth quintile	262,728	(20.8)	229,383	(20.8)
Fifth quintile – most deprived	255,088	(20.2)	222,780	(20.2)
Material Deprivation[a]				
First quintile – least deprived	243,273	(19.3)	207,631	(18.8)
Second quintile	235,659	(18.7)	203,478	(18.5)
Third quintile	254,609	(20.2)	221,696	(20.1)
Fourth quintile	263,184	(20.8)	232,093	(21.1)
Fifth quintile – most deprived	265,980	(21.1)	237,132	(21.5)
Health outcome				
1-year mortality	54,022	(3.8)	na	
Polypharmacy (≥10/y)	na		462,957	(37.6)
Frequent visits to ED (≥3/y)	na		57,993	(4.7)
Frequent visits to GP (≥7/y)	na		113,384	(9.2)
Frequent visits to specialist physicians (≥10/y)	na		154,991	(12.6)
Hospitalisation (≥1/y)	na		147,469	(12.0)

Abbreviation: ED: emergency department; GP: general practitioners; na: not applicable; SD: standard deviation; y: year

a 168,274 individuals in the main cohort and 129,626 individuals in the subcohort have a missing value for this variable

b The main cohort included all individuals aged over 65 on April 1st, 2019 and registered in the QICDSS from April 1st, 1999 to March 31th, 2020 or until death. This cohort is used to assess the impact of lookback on prevalence and mortality prediction

c The subcohort excludes from de main cohort: 1) people without continuous public drug plan coverage from April 1st, 2019 to March 31, 2020; 2) deceased people between April 1st, 2019 to March 31th, 2020. The subcohort is used to assess the impact of lookback on health outcomes (except mortality).
