# Supplementary material for: Multimorbidity prevalence and health outcome prediction: assessing the impact of lookback periods, disease count, and definition criteria in health administrative data at the population-based level
Source: BMC Med Res Methodol. 2024 May 16;24:113. doi: 10.1186/s12874-024-02243-0 (PMC11097445; doi:10.1186/s12874-024-02243-0)
Supplement: Supplementary file 2 — Supplementary Material 2. Additional file 2. results of supplementary and sensitivity analysis [file 12874_2024_2243_MOESM2_ESM.pdf]

Supplemental Digital Content 02

Prevalence for all lists of diseases

Table A2.1: Prevalence of multimorbidity by criterion used to define multimorbidity, length of lookback period, list of diseases, and type of case definition for study cohort ages >65 on April 1st, 2019, Québec (Canada), n = 1,430,979

Table A2.1.1: prevalence of each specific disease in the All-inclusive list (L60) using the unique case definition by length of lookback period (1, 5, 10, 15, 20 years) for study cohort ages >65 on April 1st, 2019, Québec (Canada), n = 1,430,979

Table A2.1.2: prevalence of each specific disease in the Core list (L20) using the unique case definition by length of lookback period (1, 5, 10, 15, 20 years) for study cohort ages >65 on April 1st, 2019, Québec (Canada), n = 1,430,979

Table A2.1.3: prevalence of each specific disease in the Charlson and Elixhauser list (L31) using the unique case definition by length of lookback period (1, 5, 10, 15, 20 years) for study cohort ages >65 on April 1st, 2019, Québec (Canada), n = 1,430,979

Model performance for the All-inclusive list of diseases

Table A2.2.1: Predictive performance (c-statistic) and model adjustment (Brier scaled score) to predict 1-year mortality for the All-inclusive list (L60) by criterion used to define multimorbidity, length of lookback period, and type of case definition (unique case definition for all diseases, case definition specific for some diseases)

Table A2.2.2: Predictive performance (c-statistic) and model adjustment (Brier scaled score) to predict hospitalisation (≥1/year) for the All-inclusive (L60) by criterion used to define multimorbidity, length of lookback period, and type of case definition (unique case definition for all diseases, case definition specific for some diseases)

Table A2.2.3: Predictive performance (c-statistic) and model adjustment (Brier scaled score) to predict frequent visits to emergency department (≥3/year) for the All-inclusive list (L60) by criterion used to define multimorbidity, length of lookback period, and type of case definition (unique case definition for all diseases, case definition specific for some diseases)

Table A2.2.4: Predictive performance (c-statistic) and model adjustment (Brier scaled score) to predict frequent visits to general practitioner (≥7/year) for the All-inclusive list (L60) by criterion used to define multimorbidity, length of lookback period, and type of case definition (unique case definition for all diseases, case definition specific for some diseases)

Table A2.2.5: Predictive performance (c-statistic) and model adjustment (Brier scaled score) to predict frequent visits to specialist physician (≥10/year) for the All-inclusive list (L60) by criterion used to define multimorbidity, length of lookback period, and type of case definition (unique case definition for all diseases, case definition specific for some diseases)

Table A2.2.6: Predictive performance (c-statistic) and model adjustment (Brier scaled score) to predict polypharmacy (≥10/year) for the All-inclusive list (L60) by criterion used to define multimorbidity, length of lookback period, and type of case definition (unique case definition for all diseases, case definition specific for some diseases)

Model performance for the Core list of diseases

Table A2.3.1: Predictive performance (c-statistic) and model adjustment (Brier scaled score) to predict 1-year mortality for the Core list (L20) by criterion used to define multimorbidity, length of lookback period, and type of case definition (unique case definition for all diseases, case definition specific for some diseases with/without hypertension)

Table A2.3.2: Predictive performance (c-statistic) and model adjustment (Brier scaled score) to predict hospitalisation (≥1/year) for the Core list (L20) by criterion used to define multimorbidity, length of lookback period, and type of case definition (unique case definition for all diseases, case definition specific for some diseases with/without hypertension)

Table A2.3.3: Predictive performance (c-statistic) and model adjustment (Brier scaled score) to predict frequent visits to emergency department (≥3/year) for the Core list (L20) by criterion used to define multimorbidity, length of lookback period, and type of case definition (unique case definition for all diseases, case definition specific for some diseases with/without hypertension)

Table A2.3.4: Predictive performance (c-statistic) and model adjustment (Brier scaled score) to predict frequent visits to general practitioner (≥7/year) for the Core list (L20) by criterion used to define multimorbidity, length of lookback period, and type of case definition (unique case definition for all diseases, case definition specific for some diseases with/without hypertension)

Table A2.3.5: Predictive performance (c-statistic) and model adjustment (Brier scaled score) to predict frequent visits to specialist physician (≥10/year) for the Core list (L20) by criterion used to define multimorbidity, length of lookback period, and type of case definition (unique case definition for all diseases, case definition specific for some diseases with/without hypertension)

Table A2.3.6: Predictive performance (c-statistic) and model adjustment (Brier scaled score) to predict polypharmacy (≥10/year) for the Core list (L20) by criterion used to define multimorbidity, length of lookback period, and type of case definition (unique case definition for all diseases, case definition specific for some diseases with/without hypertension)

Model performance for the Core list of diseases

Table A2.4.1: Predictive performance (c-statistic) and model adjustment (Brier scaled score) to predict 1-year mortality for the Charlson & Elixhauser list (L31) by criterion used to define multimorbidity, length of lookback period, and type of case definition (unique case definition for all diseases, case definition specific for some diseases)

Table A2.4.2: Predictive performance (c-statistic) and model adjustment (Brier scaled score) to predict hospitalisation (≥1/year) for the Charlson & Elixhauser list (L31) by criterion used to define multimorbidity, length of lookback period, and type of case definition (unique case definition for all diseases, case definition specific for some diseases)

Table A2.4.3: Predictive performance (c-statistic) and model adjustment (Brier scaled score) to predict frequent visits to emergency department (≥3/year) for the Charlson & Elixhauser list (L31) by criterion used to define multimorbidity, length of lookback period, and type of case definition (unique case definition for all diseases, case definition specific for some diseases)

Table A2.4.4: Predictive performance (c-statistic) and model adjustment (Brier scaled score) to predict frequent visits to general practitioner (≥7/year) for the Charlson & Elixhauser list (L31) by criterion used to define multimorbidity, length of lookback period, and type of case definition (unique case definition for all diseases, case definition specific for some diseases)

Table A2.4.5: Predictive performance (c-statistic) and model adjustment (Brier scaled score) to predict frequent visits to specialist physician (≥10/year) for the Charlson & Elixhauser list (L31) by criterion used to define multimorbidity, length of lookback period, and type of case definition (unique case definition for all diseases, case definition specific for some diseases)

Table A2.4.6: Predictive performance (c-statistic) and model adjustment (Brier scaled score) to predict polypharmacy (≥10/year) for the Charlson & Elixhauser list (L31) by criterion used to define multimorbidity, length of lookback period, and type of case definition (unique case definition for all diseases, case definition specific for some diseases)

Internal validity by sex and age groups

Figure A1.1: Illustration of the length of lookback periods where the predictive performance is maximal according to sex. Light-grey areas indicate maximal predictive performance for the ≥2 chronic conditions (MM2+) definition, grey-dot-pattern areas for the ≥3 chronic conditions (MM3+) definition, and dark-grey areas for the ≥4 chronic conditions (MM4+) definition. Shaded areas indicate the length of lookback period where the c-statistic ranged in the standard error interval [±0.001] of the maximal c-statistic. The vertical black lines delineate the minimal lookback period (10 years) required to reach a "stabilized" multimorbidity prevalence.

Figure A1.2: Illustration of the length of lookback periods where the predictive performance is maximal according to age group. Light-grey areas indicate maximal predictive performance for the ≥2 chronic conditions (MM2+) definition, grey-dot-pattern areas for the ≥3 chronic conditions (MM3+) definition, and dark-grey areas for the ≥4 chronic conditions (MM4+) definition. Shaded areas indicate the length of lookback period where the c-statistic ranged in the standard error interval [±0.001] of the maximal c-statistic. The vertical black lines delineate the minimal lookback period (10 years) required to reach a "stabilized" multimorbidity prevalence.

Table A3.1: Maximal predictive performance (c-statistic) value and length of the lookback period when it is reached (year) by sex (>65 years old as of April 1st, 2019, Québec [Canada], n > 1.2 million)

Table A3.2: Maximal predictive performance (c-statistic) value and length of the lookback period when it is reached (year) by age group (>65 years old as of April 1st, 2019, Québec [Canada], n > 1.2 million)

Table A2.1: Prevalence of multimorbidity by length of lookback period, list of diseases, criterion used to define multimorbidity, and type of case definition for study cohort ages >65 on April 1st, 2019, Québec (Canada), n = 1,430,979

		List of diseases and type of case definition						
		All-inclusive list (L60)[a]		Core list (L20)[b]			Charslon & Elixhauser list (L31)[c]	
Multimorbidity definition	Lookback period (year)	Unique case definition prev. (%)	Specific case definition[d] prev. (%)	Unique case definition prev. (%)	Validated case definition[e] prev. (%)	Validated case definition with hypertension[f] prev. (%)	Unique case definition prev. (%)	Specific case definition[g] prev. (%)
MM2+	1	20.0	20.0	11.0	13.4	16.2	13.5	13.5
	2	38.8	38.8	20.7	23.3	28.4	24.4	24.4
	3	51.4	51.4	28.3	29.8	36.7	32.2	32.2
	4	61.0	61.0	34.5	34.8	43.4	38.6	38.6
	5	67.9	67.9	39.5	38.9	48.7	43.6	43.6
	6	72.9	72.6	43.5	41.5	52.3	47.6	47.3
	7	76.7	76.2	46.8	43.7	55.1	50.8	50.3
	8	79.6	79.0	49.7	45.5	57.5	53.5	52.8
	9	81.9	81.2	52.3	47.1	59.4	55.8	55.0
	10	83.8	83.0	54.5	48.4	61.0	57.8	56.8
	11	85.3	84.4	56.5	49.6	62.4	59.5	58.3
	12	86.6	85.7	58.4	50.6	63.7	61.1	59.7
	13	87.7	86.7	60.1	51.6	64.8	62.5	61.0
	14	88.6	87.6	61.6	52.5	65.7	63.7	62.1
	15	89.3	88.3	63.0	53.3	66.6	64.9	63.1
	16	90.0	89.0	64.3	54.1	67.3	65.9	63.9
	17	90.6	89.6	65.6	54.7	68.0	66.8	64.7
	18	91.1	90.1	66.7	55.4	68.5	67.7	65.4
	19	91.5	90.5	67.7	55.9	69.0	68.5	66.1
	20	91.9	90.9	68.7	56.4	69.5	69.2	66.7
MM3+	1	12.8	12.8	5.9	6.2	8.6	9.0	9.0
	2	24.9	24.9	10.7	11.3	15.6	15.6	15.6
	3	35.0	35.0	14.9	15.2	20.9	20.6	20.6
	4	43.7	43.7	18.7	18.4	25.5	24.9	24.9
	5	50.8	50.8	22.0	21.1	29.4	28.4	28.4
	6	56.5	56.2	24.9	23.0	32.2	31.4	31.2
	7	61.0	60.5	27.5	24.6	34.5	33.9	33.5
	8	64.9	64.1	29.8	26.0	36.6	36.1	35.6
	9	68.1	67.1	31.9	27.3	38.3	38.0	37.3
	10	70.8	69.6	33.9	28.4	39.8	39.7	38.9
	11	73.0	71.8	35.7	29.3	41.2	41.3	40.2
	12	75.0	73.6	37.4	30.3	42.4	42.7	41.5
	13	76.7	75.3	39.0	31.1	43.5	44.0	42.6

		List of diseases and type of case definition						
		All-inclusive list (L60)[a]		Core list (L20)[b]			Charslon & Elixhauser list (L31)[c]	
Multimorbidity definition	Lookback period (year)	Unique case definition	Specific case definition[d]	Unique case definition	Validated case definition[e]	Validated case definition with hypertension[f]	Unique case definition	Specific case definition[g]
		prev. (%)	prev. (%)	prev. (%)	prev. (%)	prev. (%)	prev. (%)	prev. (%)
	14	78.2	76.7	40.5	31.9	44.5	45.2	43.6
	15	79.5	77.9	42.0	32.6	45.4	46.3	44.6
	16	80.7	79.0	43.4	33.3	46.2	47.4	45.5
	17	81.7	80.0	44.7	34.0	46.9	48.3	46.3
	18	82.6	80.8	45.9	34.6	47.6	49.3	47.0
	19	83.4	81.6	47.0	35.1	48.2	50.2	47.7
	20	84.1	82.3	48.1	35.6	48.7	51.0	48.4
MM4+	1	10.2	10.2	3.3	3.3	5.2	6.6	6.6
	2	18.5	18.5	5.9	6.0	9.2	11.1	11.1
	3	25.8	25.8	8.3	8.1	12.4	14.5	14.5
	4	32.5	32.5	10.4	9.9	15.1	17.4	17.4
	5	38.5	38.5	12.4	11.5	17.6	19.8	19.8
	6	43.6	43.3	14.2	12.6	19.3	21.9	21.8
	7	47.9	47.4	15.8	13.6	20.9	23.7	23.4
	8	51.8	50.9	17.4	14.6	22.3	25.3	24.9
	9	55.2	54.1	18.8	15.4	23.5	26.7	26.1
	10	58.1	56.8	20.1	16.1	24.6	27.9	27.2
	11	60.7	59.2	21.4	16.8	25.5	29.1	28.2
	12	62.9	61.3	22.7	17.4	26.4	30.1	29.2
	13	65.0	63.2	23.8	18.0	27.3	31.1	30.0
	14	66.9	65.0	25.0	18.6	28.1	32.0	30.8
	15	68.5	66.5	26.1	19.1	28.8	32.9	31.5
	16	70.0	67.9	27.2	19.6	29.5	33.8	32.2
	17	71.4	69.2	28.2	20.1	30.1	34.5	32.9
	18	72.6	70.3	29.2	20.5	30.7	35.3	33.5
	19	73.7	71.3	30.1	20.9	31.2	36.1	34.1
	20	74.7	72.2	31.0	21.3	31.7	36.7	34.6

Abbreviations: MM2+: multimorbidity defined as ≥2 chronic conditions; MM3+: multimorbidity defined as ≥3 chronic conditions; MM4+: multimorbidity defined as ≥4 chronic conditions

a Prevalence of each disease in All-inclusive list (L60) with lookback periods of 1, 5, 10, 15 and 20 years are presented in Table A2.1.1

b Prevalence of each disease in Core list (L20) with lookback periods of 1, 5, 10, 15 and 20 years are presented in Table A2.1.2

c Prevalence of each disease of the Charlson and Elixhauser list (L31) with lookback periods of 1, 5, 10, 15 and 20 years are presented in Table A2.1.3

d The maximum length of lookback period is 5 years for: depression and mood diseases; Neurotic, stress-related and somatoform diseases; Sleep disorders.

e All case definitions are validated and available in Table A1.3

f All case definitions are validated and available in Table A1.3. Hypertension have been added to the Core list (L20).

g The maximum length of lookback period is 5 years for: depression; psychoses; alcohol abuse; drug abuse.

Table A2.1.1: prevalence of each specific disease in the All-inclusive list (L60) using the unique case definition by length of lookback period (1, 5, 10, 15, 20 years) for study cohort ages > 65 on April 1st, 2019, Québec (Canada), n = 1,430,979

	Prevalence (%) by length of lookback period				
Chronic disease	1 year	5 years	10 years	15 years	20 years
Allergy	0.1	0.8	1.6	2.6	3.9
Anemia	3.9	11.6	16.1	18.5	20.3
Asthma	1.1	4.1	6.3	8.0	9.5
Atrial fibrillation	3.8	8.8	10.2	10.7	10.9
Autoimmune diseases	0.8	2.1	2.9	3.4	3.8
Blindness, visual impairment	0.1	0.4	0.6	0.7	0.8
Blood and blood forming organ disease	0.6	1.9	2.8	3.3	3.7
Bradycardias and conduction diseases	1.0	2.4	2.9	3.0	3.1
Cardiac valve disease	1.9	5.2	6.4	6.9	7.2
Cataract and other lens diseases	5.0	21.0	33.1	38.4	40.3
Cerebrovascular disease	1.3	4.7	6.8	8.2	9.1
Chromosomal abnormalities	0.0	0.0	0.0	0.0	0.0
Chronic infectious diseases	0.1	0.6	1.0	1.3	1.5
Chronic kidney diseases	3.4	7.2	8.2	8.7	8.9
Chronic liver disease	0.5	1.2	1.6	1.9	2.2
Chronic pancreas, biliary tract and gallbladder diseases	0.5	2.3	4.1	5.9	8.1
Chronic ulcer of the skin	0.4	1.0	1.4	1.6	1.8
Colitis and related diseases	2.2	8.4	13.4	17.0	19.9
COPD, emphysema, chronic bronchitis	3.0	7.1	8.7	9.3	9.8
Deafness, hearing impairment	1.1	5.3	9.1	11.6	13.2
Dementia	2.9	6.0	6.5	6.7	6.7
Depression and mood diseases	1.7	6.3	10.7	14.8	19.1
Diabetes	8.3	19.6	22.4	23.3	23.7
Dorsopathy	1.4	4.8	7.4	9.6	12.0
Dyslipidemia	6.9	22.0	32.0	38.2	42.1
Ear, nose, throat diseases	0.5	3.2	6.6	9.8	13.0
Epilepsy	0.3	0.9	1.2	1.3	1.5
Esophagus, stomach and duodenum diseases	0.8	3.6	6.8	9.6	12.6
Glaucoma	3.2	10.3	13.4	15.1	16.1
Heart failure	2.6	6.3	8.1	8.9	9.4
Hematological neoplasm	1.0	1.8	2.1	2.3	2.3
Hypertension	12.5	41.0	53.9	59.2	61.4
Inflammatory arthropathies	2.2	5.9	8.3	10.2	11.8
Inflammatory bowel diseases	0.3	0.8	1.1	1.2	1.4
Ischemic heart diseases	6.0	17.5	22.2	24.7	26.2
Migraine and facial pain syndromes	0.4	2.0	3.8	5.6	8.0
Multiple sclerosis	0.1	0.2	0.3	0.3	0.3
Neurotic, stress-related and somatoform diseases	2.0	8.5	13.9	18.8	23.7
Obesity	1.0	3.6	5.7	7.3	8.7

	Prevalence (%) by length of lookback period				
Chronic disease	1 year	5 years	10 years	15 years	20 years
Osteoarthritis and other degenerative joint diseases	3.7	13.9	20.8	25.2	28.2
Osteoporosis	2.1	7.9	12.5	15.4	17.1
Other cardiovascular diseases	2.8	8.0	10.4	11.7	12.8
Other digestive diseases	0.9	3.0	4.2	4.6	4.9
Other eye diseases	2.9	8.8	12.6	14.8	16.2
Other genitourinary diseases	2.0	7.6	11.9	14.8	17.0
Other metabolic diseases	1.5	4.9	6.9	8.2	9.1
Other musculoskeletal and joint diseases	1.7	6.3	10.0	12.5	14.3
Other neurological diseases	1.1	3.3	4.6	5.3	5.9
Other Psychiatric and behavioral diseases	1.2	3.4	4.8	5.7	6.5
Other respiratory diseases	1.3	3.5	4.6	5.3	5.8
Other skin diseases	0.1	0.3	0.5	0.6	0.7
Parkinson and parkinsonism	0.7	1.3	1.4	1.5	1.5
Peripheral neuropathy	1.1	4.5	7.9	10.7	13.1
Peripheral vascular diseases	0.5	1.7	2.5	3.0	3.3
Prostate diseases	2.2	8.2	11.3	12.9	14.0
Schizophrenia and delusional diseases	0.3	0.9	1.2	1.4	1.5
Sleep disorders	1.3	4.3	6.1	7.2	7.9
Solid neoplasm	8.2	19.8	25.0	27.9	29.4
Thyroid diseases	3.2	10.2	14.2	16.5	18.0
Venous and lymphatic diseases	0.6	2.1	3.5	4.6	6.0

Table A2.1.2: prevalence of each specific disease in the Core list (L20) using the unique case definition by length of lookback period (1, 5, 10, 15, 20 years) for study cohort ages >65 on April 1st, 2019, Québec (Canada), n = 1,430,979

	Prevalence (%) by length of lookback period				
Chronic disease	1 year	5 years	10 years	15 years	20 years
Alcohol use disorder	0.5	1.4	2.1	2.5	2.9
Asthma	1.1	4.1	6.3	8.0	9.5
Blindness/vision impairment	0.1	0.4	0.6	0.7	0.8
Cancer	8.2	19.8	25.0	27.9	29.4
Chronic liver disease	0.5	1.2	1.6	1.9	2.2
Chronic pain	4.1	17.9	28.1	36.2	43.4
Chronic renal disease	3.4	7.2	8.2	8.7	8.9
COPD	3.0	7.1	8.7	9.3	9.8
Coronary heart disease	6.0	17.5	22.2	24.7	26.2
Dementia	2.9	6.0	6.5	6.7	6.7
Depression and anxiety	3.5	12.7	19.8	25.7	31.6
Diabetes	8.3	19.6	22.4	23.3	23.7
Drug use disorder	0.1	0.4	0.6	0.8	1.0
Gynaecological disorder (Excluding urinary incontinence)	1.8	6.9	10.8	13.4	15.5
Heart failure	2.6	6.3	8.1	8.9	9.4
Hypertension	12.5	41.0	53.9	59.2	61.4
Musculoskeletal impairment due to injury	0.3	1.2	1.7	2.0	2.2
Osteoarthritis	3.7	13.9	20.8	25.2	28.2
Osteoporosis	2.1	7.9	12.5	15.4	17.1
Schizophrenia	0.3	0.6	0.8	0.9	1.0
Stroke	1.3	4.7	6.8	8.2	9.1

Table A2.1.3: prevalence of each specific disease in the Charlson and Elixhauser list (L31) using the unique case definition by length of lookback period (1, 5, 10, 15, 20 years) for study cohort ages >65 on April 1st, 2019, Québec (Canada), n = 1,430,979

	Prevalence (%) by length of lookback period				
Chronic disease	1 year	5 years	10 years	15 years	20 years
AIDS/HIV	0.0	0.1	0.1	0.1	0.1
Alcohol abuse	0.5	1.4	2.1	2.5	2.9
Anemia (Blood loss anemia, Deficiency anemia)	3.8	11.3	15.7	18.0	19.8
Any tumor without metastasis	6.6	15.0	18.7	20.9	22.2
Cardiac arrhythmias	5.5	13.6	16.6	18.1	19.4
Cerebrovascular disease	1.5	5.0	7.3	8.6	9.5
Chronic pulmonary disease	4.8	13.2	17.6	20.7	23.7
Coagulopathy	0.6	1.9	2.8	3.2	3.7
Congestive heart failure	2.2	5.2	6.4	7.0	7.4
Dementia	2.8	5.7	6.1	6.2	6.2
Depression	1.4	5.6	9.8	13.8	18.1
Diabetes, complicated	1.1	3.6	4.8	5.4	5.6
Diabetes, uncomplicated	7.3	18.9	21.9	22.8	23.2
Drug abuse	0.1	0.4	0.6	0.8	1.0
Fluid and electrolyte disorders	1.9	6.0	8.2	9.0	9.6
Hypertension	12.5	41.0	53.9	59.3	61.4
Hypothyroidism	3.1	9.7	13.4	15.6	16.9
Liver disease	0.9	2.5	3.5	4.1	4.6
Metastatic cancer	1.7	5.4	7.7	9.0	9.7
Myocardial infarction	1.7	5.3	7.4	8.8	9.7
Neurological disorders	1.5	3.8	4.7	5.1	5.4
Obesity	1.0	3.6	5.7	7.3	8.7
Paralysis	0.3	1.2	1.7	2.0	2.2
Peripheral vascular disorders	2.2	6.1	8.1	9.1	9.7
Psychoses	0.5	1.4	1.9	2.2	2.4
Pulmonary circulation disorders	0.8	2.4	3.5	4.0	4.3
Renal disease	3.6	7.5	8.6	9.0	9.2
Rheumatoid arth./collagen vascular disease	1.6	3.4	4.3	4.9	5.3
Ulcer disease	0.2	0.8	1.3	1.8	2.4
Valvular disease	1.9	5.2	6.4	6.9	7.2
Weight loss	0.5	1.6	2.4	2.7	2.9

Table A2.2.1: Predictive performance (c-statistic) and model adjustment (Brier scaled score) to predict 1-year mortality for the All-inclusive list (L60) by criterion used to define multimorbidity, length of lookback period, and type of case definition (unique case definition for all diseases, case definition specific for some diseases)

		Unique case definition		Specific case definition[a]	
Multimorbidity definition	Lookback period (year)	c-statistic (95% IC)[b]	Brier scaled score	c-statistic (95% IC)[b]	Brier scaled score
Baseline model[c]	na	0.758 (0.756 - 0.760)	0.048	0.758 (0.756 - 0.760)	0.048
MM2+	1	0.793 (0.791 - 0.795)	0.056	0.793 (0.791 - 0.795)	0.056
	2	0.785 (0.783 - 0.787)	0.053	0.785 (0.783 - 0.787)	0.053
	3	0.780 (0.778 - 0.782)	0.052	0.780 (0.778 - 0.782)	0.052
	4	0.775 (0.773 - 0.777)	0.051	0.775 (0.773 - 0.777)	0.051
	5	0.772 (0.770 - 0.774)	0.051	0.772 (0.770 - 0.774)	0.051
	6	0.770 (0.768 - 0.772)	0.050	0.770 (0.768 - 0.772)	0.050
	7	0.768 (0.766 - 0.770)	0.050	0.768 (0.766 - 0.770)	0.050
	8	0.767 (0.765 - 0.769)	0.050	0.767 (0.765 - 0.769)	0.050
	9	0.766 (0.764 - 0.768)	0.050	0.766 (0.764 - 0.768)	0.050
	10	0.765 (0.763 - 0.767)	0.050	0.765 (0.763 - 0.767)	0.050
	11	0.764 (0.762 - 0.766)	0.049	0.765 (0.763 - 0.767)	0.049
	12	0.764 (0.762 - 0.766)	0.049	0.764 (0.762 - 0.766)	0.049
	13	0.763 (0.761 - 0.765)	0.049	0.764 (0.762 - 0.766)	0.049
	14	0.763 (0.761 - 0.765)	0.049	0.763 (0.761 - 0.765)	0.049
	15	0.762 (0.760 - 0.765)	0.049	0.763 (0.761 - 0.765)	0.049
	16	0.762 (0.760 - 0.764)	0.049	0.763 (0.760 - 0.765)	0.049
	17	0.762 (0.760 - 0.764)	0.049	0.762 (0.760 - 0.764)	0.049
	18	0.762 (0.759 - 0.764)	0.049	0.762 (0.760 - 0.764)	0.049
	19	0.761 (0.759 - 0.763)	0.049	0.762 (0.760 - 0.764)	0.049
	20	0.761 (0.759 - 0.763)	0.049	0.762 (0.759 - 0.764)	0.049
MM3+	1	0.799 (0.797 - 0.800)	0.061	0.799 (0.797 - 0.800)	0.061
	2	0.797 (0.795 - 0.799)	0.058	0.797 (0.795 - 0.799)	0.058
	3	0.793 (0.791 - 0.794)	0.056	0.793 (0.791 - 0.794)	0.056
	4	0.788 (0.786 - 0.790)	0.055	0.788 (0.786 - 0.790)	0.055
	5	0.784 (0.782 - 0.786)	0.054	0.784 (0.782 - 0.786)	0.054
	6	0.781 (0.779 - 0.783)	0.053	0.781 (0.779 - 0.783)	0.053
	7	0.779 (0.777 - 0.781)	0.053	0.779 (0.777 - 0.781)	0.053
	8	0.776 (0.774 - 0.778)	0.052	0.777 (0.775 - 0.779)	0.052
	9	0.774 (0.772 - 0.776)	0.052	0.775 (0.773 - 0.777)	0.052
	10	0.773 (0.771 - 0.775)	0.051	0.773 (0.771 - 0.775)	0.052
	11	0.772 (0.770 - 0.774)	0.051	0.772 (0.770 - 0.774)	0.051
	12	0.771 (0.769 - 0.773)	0.051	0.771 (0.769 - 0.773)	0.051
	13	0.770 (0.768 - 0.772)	0.051	0.770 (0.768 - 0.772)	0.051
	14	0.769 (0.767 - 0.771)	0.050	0.769 (0.767 - 0.771)	0.050

		Unique case definition		Specific case definition[a]	
Multimorbidity definition	Lookback period (year)	c-statistic (95% IC)[b]	Brier scaled score	c-statistic (95% IC)[b]	Brier scaled score
	15	0.768 (0.766 - 0.770)	0.050	0.769 (0.767 - 0.771)	0.050
	16	0.767 (0.765 - 0.769)	0.050	0.768 (0.766 - 0.770)	0.050
	17	0.767 (0.765 - 0.769)	0.050	0.768 (0.766 - 0.770)	0.050
	18	0.767 (0.765 - 0.769)	0.050	0.767 (0.765 - 0.769)	0.050
	19	0.766 (0.764 - 0.768)	0.050	0.767 (0.765 - 0.769)	0.050
	20	0.766 (0.764 - 0.768)	0.050	0.767 (0.764 - 0.769)	0.050
MM4+	1	0.799 (0.797 - 0.801)	0.063	0.799 (0.797 - 0.801)	0.063
	2	0.802 (0.800 - 0.804)	0.062	0.802 (0.800 - 0.804)	0.062
	3	0.800 (0.798 - 0.802)	0.061	0.800 (0.798 - 0.802)	0.061
	4	0.797 (0.795 - 0.799)	0.059	0.797 (0.795 - 0.799)	0.059
	5	0.794 (0.792 - 0.795)	0.058	0.794 (0.792 - 0.795)	0.058
	6	0.790 (0.788 - 0.792)	0.057	0.791 (0.789 - 0.792)	0.057
	7	0.788 (0.786 - 0.790)	0.056	0.788 (0.786 - 0.790)	0.056
	8	0.785 (0.784 - 0.787)	0.055	0.786 (0.784 - 0.788)	0.055
	9	0.783 (0.782 - 0.785)	0.055	0.784 (0.782 - 0.786)	0.055
	10	0.781 (0.780 - 0.783)	0.054	0.782 (0.780 - 0.784)	0.054
	11	0.780 (0.778 - 0.782)	0.054	0.781 (0.779 - 0.783)	0.054
	12	0.778 (0.777 - 0.780)	0.053	0.779 (0.777 - 0.781)	0.053
	13	0.777 (0.775 - 0.779)	0.053	0.778 (0.776 - 0.780)	0.053
	14	0.776 (0.774 - 0.778)	0.052	0.777 (0.775 - 0.779)	0.053
	15	0.775 (0.773 - 0.777)	0.052	0.776 (0.774 - 0.778)	0.052
	16	0.774 (0.772 - 0.776)	0.052	0.775 (0.773 - 0.777)	0.052
	17	0.773 (0.771 - 0.775)	0.052	0.774 (0.772 - 0.776)	0.052
	18	0.772 (0.771 - 0.774)	0.051	0.774 (0.772 - 0.776)	0.052
	19	0.772 (0.770 - 0.774)	0.051	0.773 (0.771 - 0.775)	0.051
	20	0.771 (0.769 - 0.773)	0.051	0.773 (0.771 - 0.774)	0.051

Abbreviations: MM2+: multimorbidity defined as ≥2 chronic conditions; MM3+: multimorbidity defined as ≥3 chronic conditions; MM4+: multimorbidity defined as ≥4 chronic conditions; na: not applicable

a The maximum length of lookback period is 5 years for: depression and mood diseases; Neurotic, stress-related and somatoform diseases; Sleep disorders.

b Shaded areas indicate the length of lookback period where the c-statistic ranged in the standard error interval [±0.001] of the maximal c-statistic.

c The baseline model includes only the covariates (age group, sex, material and social deprivation)

Table A2.2.2: Predictive performance (c-statistic) and model adjustment (Brier scaled score) to predict hospitalisation (≥1/year) for the All-inclusive list (L60) by criterion used to define multimorbidity, length of lookback period, and type of case definition (unique case definition for all diseases, case definition specific for some diseases)

Multimorbidity definition	Lookback period (year)	Unique case definition			Specific case definition[a]		
		c-statistic (95% IC)[b]		Brier scaled score	c-statistic (95% IC)[b]		Brier scaled score
Baseline model[c]	na	0.615	(0.614 - 0.617)	0.019	0.615	(0.614 - 0.617)	0.019
MM2+	1	0.668	(0.666 - 0.669)	0.042	0.668	(0.666 - 0.669)	0.042
	2	0.668	(0.666 - 0.669)	0.038	0.668	(0.666 - 0.669)	0.038
	3	0.662	(0.660 - 0.663)	0.035	0.662	(0.660 - 0.663)	0.035
	4	0.655	(0.654 - 0.657)	0.032	0.655	(0.654 - 0.657)	0.032
	5	0.650	(0.648 - 0.651)	0.030	0.650	(0.648 - 0.651)	0.030
	6	0.646	(0.644 - 0.647)	0.028	0.646	(0.645 - 0.647)	0.028
	7	0.642	(0.641 - 0.644)	0.027	0.643	(0.641 - 0.644)	0.027
	8	0.639	(0.638 - 0.641)	0.026	0.640	(0.639 - 0.642)	0.026
	9	0.637	(0.636 - 0.638)	0.025	0.638	(0.636 - 0.639)	0.025
	10	0.635	(0.634 - 0.637)	0.025	0.636	(0.634 - 0.637)	0.025
	11	0.634	(0.632 - 0.635)	0.024	0.634	(0.633 - 0.636)	0.024
	12	0.632	(0.631 - 0.634)	0.024	0.633	(0.632 - 0.635)	0.024
	13	0.631	(0.630 - 0.633)	0.023	0.632	(0.630 - 0.633)	0.024
	14	0.630	(0.629 - 0.632)	0.023	0.631	(0.629 - 0.632)	0.023
	15	0.629	(0.628 - 0.631)	0.023	0.630	(0.629 - 0.632)	0.023
	16	0.628	(0.627 - 0.630)	0.023	0.629	(0.628 - 0.631)	0.023
	17	0.628	(0.626 - 0.629)	0.022	0.629	(0.627 - 0.630)	0.023
	18	0.627	(0.625 - 0.628)	0.022	0.628	(0.627 - 0.629)	0.022
	19	0.626	(0.625 - 0.628)	0.022	0.627	(0.626 - 0.629)	0.022
	20	0.626	(0.625 - 0.628)	0.022	0.627	(0.626 - 0.629)	0.022
MM3+	1	0.665	(0.663 - 0.666)	0.046	0.665	(0.663 - 0.666)	0.046
	2	0.675	(0.674 - 0.677)	0.046	0.675	(0.674 - 0.677)	0.046
	3	0.676	(0.674 - 0.677)	0.043	0.676	(0.674 - 0.677)	0.043
	4	0.672	(0.670 - 0.673)	0.040	0.672	(0.670 - 0.673)	0.040
	5	0.667	(0.666 - 0.669)	0.037	0.667	(0.666 - 0.669)	0.037
	6	0.663	(0.662 - 0.665)	0.035	0.664	(0.662 - 0.665)	0.035
	7	0.659	(0.658 - 0.661)	0.033	0.660	(0.658 - 0.661)	0.034
	8	0.656	(0.654 - 0.657)	0.032	0.656	(0.655 - 0.658)	0.032
	9	0.653	(0.651 - 0.654)	0.031	0.654	(0.652 - 0.655)	0.031
	10	0.650	(0.649 - 0.652)	0.030	0.651	(0.650 - 0.653)	0.030
	11	0.648	(0.647 - 0.649)	0.029	0.649	(0.648 - 0.651)	0.029
	12	0.646	(0.644 - 0.647)	0.028	0.647	(0.646 - 0.649)	0.029
	13	0.644	(0.643 - 0.646)	0.027	0.646	(0.644 - 0.647)	0.028
	14	0.643	(0.641 - 0.644)	0.027	0.644	(0.643 - 0.646)	0.028
	15	0.641	(0.640 - 0.643)	0.026	0.643	(0.641 - 0.644)	0.027
	16	0.640	(0.638 - 0.641)	0.026	0.642	(0.640 - 0.643)	0.027
	17	0.639	(0.637 - 0.640)	0.026	0.641	(0.639 - 0.642)	0.026

Multimorbidity definition	Lookback period (year)	Unique case definition c-statistic (95% IC)[b]		Unique case definition Brier scaled score	Specific case definition[a] c-statistic (95% IC)[b]		Specific case definition[a] Brier scaled score
	18	0.638	(0.636 - 0.639)	0.025	0.640	(0.638 - 0.641)	0.026
	19	0.637	(0.635 - 0.638)	0.025	0.639	(0.637 - 0.640)	0.026
	20	0.636	(0.635 - 0.637)	0.025	0.638	(0.637 - 0.639)	0.025
MM4+	1	0.660	(0.659 - 0.662)	0.047	0.660	(0.659 - 0.662)	0.047
	2	0.675	(0.673 - 0.676)	0.050	0.675	(0.673 - 0.676)	0.050
	3	0.679	(0.677 - 0.680)	0.048	0.679	(0.677 - 0.680)	0.048
	4	0.679	(0.677 - 0.680)	0.045	0.679	(0.677 - 0.680)	0.045
	5	0.677	(0.676 - 0.679)	0.043	0.677	(0.676 - 0.679)	0.043
	6	0.674	(0.673 - 0.676)	0.041	0.675	(0.673 - 0.676)	0.041
	7	0.672	(0.671 - 0.673)	0.040	0.673	(0.671 - 0.674)	0.040
	8	0.669	(0.668 - 0.670)	0.038	0.670	(0.668 - 0.671)	0.038
	9	0.667	(0.665 - 0.668)	0.037	0.667	(0.666 - 0.669)	0.037
	10	0.664	(0.663 - 0.665)	0.035	0.665	(0.664 - 0.666)	0.036
	11	0.661	(0.660 - 0.663)	0.034	0.663	(0.661 - 0.664)	0.035
	12	0.659	(0.658 - 0.661)	0.033	0.661	(0.659 - 0.662)	0.034
	13	0.657	(0.656 - 0.658)	0.032	0.659	(0.657 - 0.660)	0.033
	14	0.655	(0.653 - 0.656)	0.031	0.657	(0.655 - 0.658)	0.032
	15	0.653	(0.652 - 0.655)	0.031	0.655	(0.654 - 0.657)	0.032
	16	0.652	(0.650 - 0.653)	0.030	0.654	(0.653 - 0.655)	0.031
	17	0.650	(0.649 - 0.652)	0.030	0.653	(0.651 - 0.654)	0.030
	18	0.649	(0.648 - 0.650)	0.029	0.651	(0.650 - 0.653)	0.030
	19	0.648	(0.646 - 0.649)	0.029	0.650	(0.649 - 0.652)	0.030
	20	0.647	(0.645 - 0.648)	0.028	0.650	(0.648 - 0.651)	0.029

Abbreviations: MM2+: multimorbidity defined as ≥2 chronic conditions; MM3+: multimorbidity defined as ≥3 chronic conditions; MM4+: multimorbidity defined as ≥4 chronic conditions; na: not applicable

a The maximum length of lookback period is 5 years for: depression and mood diseases; Neurotic, stress-related and somatoform diseases; Sleep disorders.

b Shaded areas indicate the length of lookback period where the c-statistic ranged in the standard error interval [±0.001] of the maximal c-statistic.

c The baseline model includes only the covariates (age group, sex, material and social deprivation)

Table A2.2.3: Predictive performance (c-statistic) and model adjustment (Brier scaled score) to predict <u>frequent visits to emergency department (≥3/year) for the All-inclusive list (L60)</u> by criterion used to define multimorbidity, length of lookback period, and type of case definition (unique case definition for all diseases, case definition specific for some diseases)

Multimorbidity definition	Lookback period (year)	Unique case definition			Specific case definition[a]		
		c-statistic (95% IC)[b]		Brier scaled score	c-statistic (95% IC)[b]		Brier scaled score
Baseline model[c]	na	0.634	(0.632 - 0.636)	0.011	0.634	(0.632 - 0.636)	0.011
MM2+	1	0.696	(0.693 - 0.698)	0.027	0.696	(0.693 - 0.698)	0.027
	2	0.696	(0.694 - 0.698)	0.024	0.696	(0.694 - 0.698)	0.024
	3	0.691	(0.689 - 0.693)	0.021	0.691	(0.689 - 0.693)	0.021
	4	0.684	(0.682 - 0.686)	0.019	0.684	(0.682 - 0.686)	0.019
	5	0.677	(0.675 - 0.679)	0.018	0.677	(0.675 - 0.679)	0.018
	6	0.672	(0.670 - 0.674)	0.017	0.672	(0.670 - 0.674)	0.017
	7	0.667	(0.665 - 0.669)	0.016	0.668	(0.665 - 0.670)	0.016
	8	0.664	(0.662 - 0.666)	0.015	0.665	(0.662 - 0.667)	0.015
	9	0.661	(0.659 - 0.663)	0.015	0.661	(0.659 - 0.664)	0.015
	10	0.658	(0.656 - 0.660)	0.014	0.659	(0.657 - 0.661)	0.014
	11	0.656	(0.654 - 0.658)	0.014	0.657	(0.655 - 0.659)	0.014
	12	0.654	(0.652 - 0.656)	0.014	0.655	(0.653 - 0.657)	0.014
	13	0.653	(0.651 - 0.655)	0.013	0.654	(0.652 - 0.656)	0.014
	14	0.652	(0.649 - 0.654)	0.013	0.653	(0.650 - 0.655)	0.013
	15	0.650	(0.648 - 0.653)	0.013	0.651	(0.649 - 0.654)	0.013
	16	0.649	(0.647 - 0.652)	0.013	0.650	(0.648 - 0.653)	0.013
	17	0.648	(0.646 - 0.651)	0.013	0.650	(0.647 - 0.652)	0.013
	18	0.648	(0.646 - 0.650)	0.013	0.649	(0.647 - 0.651)	0.013
	19	0.647	(0.645 - 0.649)	0.013	0.648	(0.646 - 0.650)	0.013
	20	0.647	(0.644 - 0.649)	0.012	0.648	(0.646 - 0.650)	0.013
MM3+	1	0.694	(0.692 - 0.697)	0.031	0.694	(0.692 - 0.697)	0.031
	2	0.706	(0.704 - 0.708)	0.030	0.706	(0.704 - 0.708)	0.030
	3	0.708	(0.706 - 0.710)	0.028	0.708	(0.706 - 0.710)	0.028
	4	0.704	(0.702 - 0.706)	0.025	0.704	(0.702 - 0.706)	0.025
	5	0.699	(0.697 - 0.701)	0.023	0.699	(0.697 - 0.701)	0.023
	6	0.694	(0.692 - 0.696)	0.021	0.694	(0.692 - 0.696)	0.021
	7	0.688	(0.686 - 0.690)	0.020	0.689	(0.687 - 0.691)	0.020
	8	0.684	(0.682 - 0.686)	0.019	0.685	(0.683 - 0.687)	0.019
	9	0.680	(0.678 - 0.682)	0.018	0.681	(0.679 - 0.683)	0.018
	10	0.677	(0.675 - 0.679)	0.018	0.678	(0.676 - 0.680)	0.018
	11	0.674	(0.672 - 0.676)	0.017	0.675	(0.673 - 0.678)	0.017
	12	0.672	(0.669 - 0.674)	0.016	0.673	(0.671 - 0.675)	0.017
	13	0.669	(0.667 - 0.671)	0.016	0.671	(0.669 - 0.673)	0.016
	14	0.667	(0.665 - 0.669)	0.016	0.669	(0.667 - 0.671)	0.016
	15	0.665	(0.663 - 0.667)	0.015	0.667	(0.665 - 0.669)	0.016
	16	0.663	(0.661 - 0.666)	0.015	0.665	(0.663 - 0.667)	0.015

Multimorbidity definition	Lookback period (year)	Unique case definition: c-statistic (95% IC)[b]		Unique case definition: Brier scaled score	Specific case definition[a]: c-statistic (95% IC)[b]		Specific case definition[a]: Brier scaled score
	17	0.662	(0.660 - 0.664)	0.015	0.664	(0.662 - 0.666)	0.015
	18	0.661	(0.659 - 0.663)	0.015	0.663	(0.661 - 0.665)	0.015
	19	0.660	(0.658 - 0.662)	0.014	0.662	(0.660 - 0.664)	0.015
	20	0.659	(0.657 - 0.661)	0.014	0.661	(0.659 - 0.663)	0.015
MM4+	1	0.691	(0.688 - 0.693)	0.032	0.691	(0.688 - 0.693)	0.032
	2	0.707	(0.705 - 0.709)	0.033	0.707	(0.705 - 0.709)	0.033
	3	0.712	(0.710 - 0.715)	0.032	0.712	(0.710 - 0.715)	0.032
	4	0.714	(0.711 - 0.716)	0.030	0.714	(0.711 - 0.716)	0.030
	5	0.712	(0.710 - 0.714)	0.028	0.712	(0.710 - 0.714)	0.028
	6	0.709	(0.707 - 0.711)	0.026	0.709	(0.707 - 0.711)	0.026
	7	0.706	(0.704 - 0.708)	0.025	0.706	(0.704 - 0.708)	0.025
	8	0.702	(0.700 - 0.704)	0.024	0.703	(0.701 - 0.705)	0.024
	9	0.698	(0.696 - 0.701)	0.023	0.699	(0.697 - 0.701)	0.023
	10	0.695	(0.693 - 0.697)	0.022	0.696	(0.694 - 0.698)	0.022
	11	0.692	(0.690 - 0.694)	0.021	0.693	(0.691 - 0.695)	0.021
	12	0.689	(0.687 - 0.691)	0.020	0.690	(0.688 - 0.692)	0.020
	13	0.686	(0.684 - 0.688)	0.019	0.688	(0.686 - 0.690)	0.020
	14	0.683	(0.681 - 0.685)	0.019	0.686	(0.684 - 0.688)	0.019
	15	0.682	(0.680 - 0.684)	0.018	0.684	(0.682 - 0.686)	0.019
	16	0.680	(0.678 - 0.682)	0.018	0.682	(0.680 - 0.684)	0.018
	17	0.677	(0.675 - 0.679)	0.018	0.680	(0.678 - 0.682)	0.018
	18	0.676	(0.674 - 0.678)	0.017	0.678	(0.676 - 0.681)	0.018
	19	0.674	(0.672 - 0.676)	0.017	0.677	(0.675 - 0.679)	0.017
	20	0.672	(0.670 - 0.674)	0.017	0.675	(0.673 - 0.678)	0.017

Abbreviations: MM2+: multimorbidity defined as ≥2 chronic conditions; MM3+: multimorbidity defined as ≥3 chronic conditions; MM4+: multimorbidity defined as ≥4 chronic conditions; na: not applicable

a The maximum length of lookback period is 5 years for: depression and mood diseases; Neurotic, stress-related and somatoform diseases; Sleep disorders.

b Shaded areas indicate the length of lookback period where the c-statistic ranged in the standard error interval [±0.001] of the maximal c-statistic.

c The baseline model includes only the covariates (age group, sex, material and social deprivation)

Table A2.2.4: Predictive performance (c-statistic) and model adjustment (Brier scaled score) to predict frequent visits to general practitioner (≥7/year) for the All-inclusive list (L60) by criterion used to define multimorbidity, length of lookback period, and type of case definition (unique case definition for all diseases, case definition specific for some diseases)

		Unique case definition			Specific case definition[a]		
Multimorbidity definition	Lookback period (year)	c-statistic (95% IC)[b]		Brier scaled score	c-statistic (95% IC)[b]		Brier scaled score
Baseline model[c]	na	0.629	(0.627 - 0.631)	0.020	0.629	(0.627 - 0.631)	0.020
MM2+	1	0.667	(0.665 - 0.669)	0.036	0.667	(0.665 - 0.669)	0.036
	2	0.679	(0.677 - 0.680)	0.037	0.679	(0.677 - 0.680)	0.037
	3	0.682	(0.681 - 0.684)	0.037	0.682	(0.681 - 0.684)	0.037
	4	0.680	(0.678 - 0.681)	0.035	0.680	(0.678 - 0.681)	0.035
	5	0.676	(0.674 - 0.677)	0.033	0.676	(0.674 - 0.677)	0.033
	6	0.671	(0.670 - 0.673)	0.032	0.671	(0.670 - 0.673)	0.032
	7	0.667	(0.666 - 0.669)	0.030	0.668	(0.666 - 0.669)	0.030
	8	0.664	(0.662 - 0.666)	0.029	0.664	(0.663 - 0.666)	0.029
	9	0.661	(0.659 - 0.662)	0.028	0.661	(0.660 - 0.663)	0.028
	10	0.658	(0.657 - 0.660)	0.028	0.659	(0.657 - 0.660)	0.028
	11	0.656	(0.654 - 0.658)	0.027	0.657	(0.655 - 0.658)	0.027
	12	0.654	(0.653 - 0.656)	0.027	0.655	(0.653 - 0.657)	0.027
	13	0.653	(0.651 - 0.654)	0.026	0.654	(0.652 - 0.655)	0.026
	14	0.651	(0.650 - 0.653)	0.026	0.652	(0.651 - 0.654)	0.026
	15	0.650	(0.648 - 0.652)	0.025	0.651	(0.649 - 0.653)	0.026
	16	0.649	(0.647 - 0.650)	0.025	0.650	(0.648 - 0.652)	0.025
	17	0.648	(0.646 - 0.649)	0.025	0.649	(0.647 - 0.651)	0.025
	18	0.647	(0.645 - 0.648)	0.025	0.648	(0.646 - 0.650)	0.025
	19	0.646	(0.644 - 0.647)	0.024	0.647	(0.646 - 0.649)	0.025
	20	0.645	(0.644 - 0.647)	0.024	0.647	(0.645 - 0.648)	0.025
MM3+	1	0.658	(0.656 - 0.659)	0.033	0.658	(0.656 - 0.659)	0.033
	2	0.674	(0.672 - 0.676)	0.038	0.674	(0.672 - 0.676)	0.038
	3	0.684	(0.682 - 0.686)	0.040	0.684	(0.682 - 0.686)	0.040
	4	0.688	(0.687 - 0.690)	0.040	0.688	(0.687 - 0.690)	0.040
	5	0.689	(0.687 - 0.691)	0.039	0.689	(0.687 - 0.691)	0.039
	6	0.687	(0.686 - 0.689)	0.038	0.687	(0.685 - 0.689)	0.038
	7	0.685	(0.683 - 0.686)	0.037	0.685	(0.683 - 0.686)	0.037
	8	0.682	(0.680 - 0.683)	0.035	0.682	(0.680 - 0.683)	0.035
	9	0.680	(0.678 - 0.681)	0.035	0.680	(0.678 - 0.681)	0.035
	10	0.677	(0.676 - 0.679)	0.034	0.677	(0.676 - 0.679)	0.034
	11	0.675	(0.673 - 0.676)	0.033	0.675	(0.674 - 0.677)	0.033
	12	0.672	(0.671 - 0.674)	0.032	0.673	(0.671 - 0.674)	0.032
	13	0.670	(0.668 - 0.671)	0.031	0.671	(0.669 - 0.673)	0.031
	14	0.668	(0.666 - 0.669)	0.030	0.669	(0.667 - 0.671)	0.031
	15	0.666	(0.664 - 0.667)	0.030	0.667	(0.666 - 0.669)	0.030
	16	0.664	(0.663 - 0.666)	0.029	0.666	(0.665 - 0.668)	0.030

Multimorbidity definition	Lookback period (year)	Unique case definition c-statistic (95% IC)[b]		Unique case definition Brier scaled score	Specific case definition[a] c-statistic (95% IC)[b]		Specific case definition[a] Brier scaled score
	17	0.663	(0.661 - 0.665)	0.029	0.665	(0.663 - 0.666)	0.030
	18	0.662	(0.660 - 0.663)	0.029	0.664	(0.662 - 0.665)	0.029
	19	0.661	(0.659 - 0.662)	0.028	0.663	(0.661 - 0.664)	0.029
	20	0.659	(0.658 - 0.661)	0.028	0.662	(0.660 - 0.663)	0.029
MM4+	1	0.653	(0.652 - 0.655)	0.031	0.653	(0.652 - 0.655)	0.031
	2	0.667	(0.666 - 0.669)	0.036	0.667	(0.666 - 0.669)	0.036
	3	0.678	(0.677 - 0.680)	0.040	0.678	(0.677 - 0.680)	0.040
	4	0.686	(0.684 - 0.688)	0.041	0.686	(0.684 - 0.688)	0.041
	5	0.690	(0.688 - 0.692)	0.042	0.690	(0.688 - 0.692)	0.042
	6	0.692	(0.691 - 0.694)	0.042	0.692	(0.691 - 0.694)	0.042
	7	0.693	(0.692 - 0.695)	0.042	0.693	(0.691 - 0.694)	0.041
	8	0.692	(0.691 - 0.694)	0.041	0.692	(0.690 - 0.693)	0.041
	9	0.692	(0.690 - 0.693)	0.040	0.691	(0.690 - 0.693)	0.040
	10	0.690	(0.689 - 0.692)	0.039	0.690	(0.688 - 0.691)	0.039
	11	0.689	(0.687 - 0.690)	0.038	0.689	(0.687 - 0.690)	0.038
	12	0.687	(0.686 - 0.689)	0.037	0.687	(0.686 - 0.689)	0.038
	13	0.686	(0.684 - 0.687)	0.037	0.686	(0.684 - 0.688)	0.037
	14	0.684	(0.682 - 0.685)	0.036	0.684	(0.683 - 0.686)	0.036
	15	0.682	(0.680 - 0.683)	0.035	0.683	(0.681 - 0.684)	0.036
	16	0.680	(0.678 - 0.681)	0.035	0.681	(0.680 - 0.683)	0.035
	17	0.678	(0.677 - 0.680)	0.034	0.680	(0.679 - 0.682)	0.035
	18	0.677	(0.676 - 0.679)	0.034	0.679	(0.677 - 0.680)	0.034
	19	0.676	(0.674 - 0.677)	0.033	0.678	(0.676 - 0.679)	0.034
	20	0.674	(0.673 - 0.676)	0.033	0.677	(0.675 - 0.678)	0.033

Abbreviations: MM2+: multimorbidity defined as ≥2 chronic conditions; MM3+: multimorbidity defined as ≥3 chronic conditions; MM4+: multimorbidity defined as ≥4 chronic conditions; na: not applicable

a The maximum length of lookback period is 5 years for: depression and mood diseases; Neurotic, stress-related and somatoform diseases; Sleep disorders.

b Shaded areas indicate the length of lookback period where the c-statistic ranged in the standard error interval [±0.001] of the maximal c-statistic.

c The baseline model includes only the covariates (age group, sex, material and social deprivation)

Table A2.2.5: Predictive performance (c-statistic) and model adjustment (Brier scaled score) to predict frequent visits to specialist physician (≥10/year) for the All-inclusive list (L60) by criterion used to define multimorbidity, length of lookback period, and type of case definition (unique case definition for all diseases, case definition specific for some diseases)

Multimorbidity definition	Lookback period (year)	Unique case definition			Specific case definition[a]		
		c-statistic (95% IC)[b]		Brier scaled score	c-statistic (95% IC)[b]		Brier scaled score
Baseline model[c]	na	0.572	(0.570 - 0.573)	0.007	0.572	(0.570 - 0.573)	0.007
MM2+	1	0.677	(0.675 - 0.678)	0.066	0.677	(0.675 - 0.678)	0.066
	2	0.695	(0.694 - 0.696)	0.060	0.695	(0.694 - 0.696)	0.060
	3	0.686	(0.684 - 0.687)	0.050	0.686	(0.684 - 0.687)	0.050
	4	0.671	(0.670 - 0.672)	0.042	0.671	(0.670 - 0.672)	0.042
	5	0.657	(0.656 - 0.658)	0.035	0.657	(0.656 - 0.658)	0.035
	6	0.645	(0.644 - 0.647)	0.031	0.646	(0.645 - 0.647)	0.031
	7	0.636	(0.634 - 0.637)	0.027	0.637	(0.636 - 0.638)	0.028
	8	0.628	(0.627 - 0.629)	0.025	0.630	(0.628 - 0.631)	0.025
	9	0.622	(0.621 - 0.623)	0.023	0.624	(0.622 - 0.625)	0.023
	10	0.617	(0.615 - 0.618)	0.021	0.619	(0.617 - 0.620)	0.022
	11	0.613	(0.611 - 0.614)	0.020	0.615	(0.614 - 0.616)	0.020
	12	0.609	(0.608 - 0.611)	0.019	0.612	(0.610 - 0.613)	0.019
	13	0.606	(0.605 - 0.608)	0.018	0.609	(0.607 - 0.610)	0.018
	14	0.604	(0.603 - 0.605)	0.017	0.606	(0.605 - 0.608)	0.018
	15	0.602	(0.601 - 0.603)	0.016	0.604	(0.603 - 0.606)	0.017
	16	0.600	(0.599 - 0.602)	0.016	0.602	(0.601 - 0.604)	0.016
	17	0.599	(0.597 - 0.600)	0.015	0.601	(0.599 - 0.602)	0.016
	18	0.597	(0.596 - 0.598)	0.015	0.599	(0.598 - 0.601)	0.016
	19	0.596	(0.594 - 0.597)	0.014	0.598	(0.597 - 0.600)	0.015
	20	0.595	(0.593 - 0.596)	0.014	0.597	(0.596 - 0.599)	0.015
MM3+	1	0.643	(0.641 - 0.644)	0.049	0.643	(0.641 - 0.644)	0.049
	2	0.679	(0.678 - 0.681)	0.062	0.679	(0.678 - 0.681)	0.062
	3	0.692	(0.691 - 0.693)	0.062	0.692	(0.691 - 0.693)	0.062
	4	0.692	(0.691 - 0.694)	0.057	0.692	(0.691 - 0.694)	0.057
	5	0.686	(0.685 - 0.688)	0.051	0.686	(0.685 - 0.688)	0.051
	6	0.678	(0.677 - 0.679)	0.045	0.679	(0.677 - 0.680)	0.046
	7	0.670	(0.668 - 0.671)	0.041	0.671	(0.670 - 0.672)	0.042
	8	0.662	(0.661 - 0.663)	0.038	0.664	(0.662 - 0.665)	0.038
	9	0.655	(0.654 - 0.657)	0.035	0.658	(0.656 - 0.659)	0.036
	10	0.649	(0.648 - 0.651)	0.032	0.652	(0.650 - 0.653)	0.033
	11	0.644	(0.643 - 0.645)	0.030	0.647	(0.645 - 0.648)	0.031
	12	0.639	(0.638 - 0.640)	0.028	0.642	(0.641 - 0.644)	0.030
	13	0.635	(0.633 - 0.636)	0.027	0.638	(0.637 - 0.640)	0.028
	14	0.631	(0.630 - 0.633)	0.025	0.635	(0.633 - 0.636)	0.027
	15	0.628	(0.626 - 0.629)	0.024	0.632	(0.630 - 0.633)	0.026
	16	0.625	(0.623 - 0.626)	0.023	0.629	(0.627 - 0.630)	0.025

Multimorbidity definition	Lookback period (year)	Unique case definition			Specific case definition[a]		
		c-statistic (95% IC)[b]		Brier scaled score	c-statistic (95% IC)[b]		Brier scaled score
	17	0.622	(0.621 - 0.624)	0.022	0.626	(0.625 - 0.628)	0.024
	18	0.620	(0.619 - 0.621)	0.022	0.624	(0.623 - 0.626)	0.023
	19	0.618	(0.617 - 0.619)	0.021	0.622	(0.621 - 0.624)	0.023
	20	0.616	(0.615 - 0.618)	0.020	0.621	(0.619 - 0.622)	0.022
MM4+	1	0.626	(0.625 - 0.628)	0.039	0.626	(0.625 - 0.628)	0.039
	2	0.658	(0.656 - 0.659)	0.054	0.658	(0.656 - 0.659)	0.054
	3	0.677	(0.675 - 0.678)	0.061	0.677	(0.675 - 0.678)	0.061
	4	0.687	(0.686 - 0.689)	0.061	0.687	(0.686 - 0.689)	0.061
	5	0.691	(0.689 - 0.692)	0.059	0.691	(0.689 - 0.692)	0.059
	6	0.690	(0.688 - 0.691)	0.055	0.690	(0.688 - 0.691)	0.056
	7	0.687	(0.686 - 0.688)	0.052	0.688	(0.686 - 0.689)	0.053
	8	0.683	(0.681 - 0.684)	0.049	0.684	(0.682 - 0.685)	0.049
	9	0.679	(0.677 - 0.680)	0.046	0.680	(0.679 - 0.681)	0.047
	10	0.674	(0.673 - 0.676)	0.043	0.676	(0.675 - 0.677)	0.044
	11	0.670	(0.668 - 0.671)	0.041	0.672	(0.671 - 0.673)	0.042
	12	0.665	(0.664 - 0.667)	0.039	0.668	(0.667 - 0.669)	0.040
	13	0.661	(0.660 - 0.663)	0.037	0.665	(0.663 - 0.666)	0.039
	14	0.658	(0.656 - 0.659)	0.035	0.661	(0.660 - 0.663)	0.037
	15	0.654	(0.653 - 0.655)	0.034	0.658	(0.657 - 0.659)	0.036
	16	0.651	(0.649 - 0.652)	0.032	0.655	(0.654 - 0.656)	0.034
	17	0.647	(0.646 - 0.649)	0.031	0.652	(0.651 - 0.654)	0.033
	18	0.645	(0.643 - 0.646)	0.030	0.650	(0.649 - 0.651)	0.032
	19	0.642	(0.641 - 0.643)	0.029	0.647	(0.646 - 0.649)	0.031
	20	0.639	(0.638 - 0.641)	0.028	0.645	(0.644 - 0.647)	0.030

Abbreviations: MM2+: multimorbidity defined as ≥2 chronic conditions; MM3+: multimorbidity defined as ≥3 chronic conditions; MM4+: multimorbidity defined as ≥4 chronic conditions; na: not applicable

a The maximum length of lookback period is 5 years for: depression and mood diseases; Neurotic, stress-related and somatoform diseases; Sleep disorders.

b Shaded areas indicate the length of lookback period where the c-statistic ranged in the standard error interval [±0.001] of the maximal c-statistic.

c The baseline model includes only the covariates (age group, sex, material and social deprivation)

Table A2.2.6: Predictive performance (c-statistic) and model adjustment (Brier scaled score) to predict polypharmacy (≥10/year) for the All-inclusive list (L60) by criterion used to define multimorbidity, length of lookback period, and type of case definition (unique case definition for all diseases, case definition specific for some diseases)

Multimorbidity definition	Lookback period (year)	Unique case definition: c-statistic (95% IC)[b]		Unique case definition: Brier scaled score	Specific case definition[a]: c-statistic (95% IC)[b]		Specific case definition[a]: Brier scaled score
Baseline model[c]	na	0.621	(0.620 - 0.622)	0.042	0.621	(0.620 - 0.622)	0.042
MM2+	1	0.680	(0.679 - 0.681)	0.100	0.680	(0.679 - 0.681)	0.100
	2	0.704	(0.703 - 0.705)	0.120	0.704	(0.703 - 0.705)	0.120
	3	0.710	(0.709 - 0.711)	0.125	0.710	(0.709 - 0.711)	0.125
	4	0.706	(0.705 - 0.707)	0.122	0.706	(0.705 - 0.707)	0.122
	5	0.700	(0.699 - 0.701)	0.117	0.700	(0.699 - 0.701)	0.117
	6	0.693	(0.693 - 0.694)	0.111	0.694	(0.693 - 0.695)	0.112
	7	0.687	(0.686 - 0.688)	0.106	0.688	(0.687 - 0.689)	0.107
	8	0.682	(0.681 - 0.683)	0.102	0.683	(0.682 - 0.684)	0.103
	9	0.677	(0.676 - 0.678)	0.098	0.678	(0.677 - 0.679)	0.099
	10	0.673	(0.672 - 0.674)	0.094	0.674	(0.673 - 0.675)	0.095
	11	0.669	(0.668 - 0.670)	0.091	0.671	(0.670 - 0.672)	0.093
	12	0.666	(0.665 - 0.667)	0.088	0.668	(0.667 - 0.669)	0.090
	13	0.663	(0.662 - 0.664)	0.086	0.665	(0.664 - 0.666)	0.088
	14	0.661	(0.660 - 0.662)	0.083	0.663	(0.662 - 0.664)	0.086
	15	0.659	(0.658 - 0.660)	0.081	0.661	(0.660 - 0.662)	0.084
	16	0.657	(0.656 - 0.658)	0.080	0.659	(0.658 - 0.660)	0.082
	17	0.655	(0.654 - 0.656)	0.078	0.657	(0.656 - 0.658)	0.080
	18	0.653	(0.652 - 0.654)	0.076	0.656	(0.655 - 0.657)	0.079
	19	0.652	(0.651 - 0.653)	0.075	0.655	(0.654 - 0.656)	0.078
	20	0.651	(0.650 - 0.652)	0.074	0.653	(0.652 - 0.654)	0.077
MM3+	1	0.667	(0.666 - 0.668)	0.092	0.667	(0.666 - 0.668)	0.092
	2	0.697	(0.696 - 0.698)	0.121	0.697	(0.696 - 0.698)	0.121
	3	0.714	(0.713 - 0.715)	0.138	0.714	(0.713 - 0.715)	0.138
	4	0.721	(0.720 - 0.722)	0.144	0.721	(0.720 - 0.722)	0.144
	5	0.723	(0.722 - 0.724)	0.145	0.723	(0.722 - 0.724)	0.145
	6	0.721	(0.720 - 0.722)	0.143	0.721	(0.720 - 0.722)	0.143
	7	0.717	(0.716 - 0.718)	0.139	0.718	(0.717 - 0.719)	0.140
	8	0.713	(0.712 - 0.714)	0.135	0.714	(0.713 - 0.715)	0.136
	9	0.709	(0.708 - 0.710)	0.131	0.710	(0.709 - 0.711)	0.133
	10	0.705	(0.704 - 0.706)	0.128	0.707	(0.706 - 0.707)	0.129
	11	0.701	(0.700 - 0.702)	0.124	0.703	(0.702 - 0.704)	0.126
	12	0.697	(0.697 - 0.698)	0.121	0.700	(0.699 - 0.701)	0.123
	13	0.694	(0.693 - 0.695)	0.118	0.697	(0.696 - 0.698)	0.121
	14	0.691	(0.690 - 0.692)	0.115	0.694	(0.693 - 0.695)	0.118
	15	0.688	(0.687 - 0.689)	0.112	0.691	(0.690 - 0.692)	0.116
	16	0.685	(0.684 - 0.686)	0.109	0.689	(0.688 - 0.690)	0.114
	17	0.683	(0.682 - 0.684)	0.107	0.687	(0.686 - 0.688)	0.112

Multimorbidity definition	Lookback period (year)	Unique case definition c-statistic (95% IC)[b]		Unique case definition Brier scaled score	Specific case definition[a] c-statistic (95% IC)[b]		Specific case definition[a] Brier scaled score
	18	0.681	(0.680 - 0.681)	0.105	0.685	(0.684 - 0.686)	0.110
	19	0.678	(0.677 - 0.679)	0.103	0.683	(0.682 - 0.684)	0.108
	20	0.676	(0.676 - 0.677)	0.101	0.681	(0.680 - 0.682)	0.106
MM4+	1	0.661	(0.660 - 0.662)	0.090	0.661	(0.660 - 0.662)	0.090
	2	0.688	(0.687 - 0.689)	0.118	0.688	(0.687 - 0.689)	0.118
	3	0.708	(0.707 - 0.709)	0.139	0.708	(0.707 - 0.709)	0.139
	4	0.720	(0.719 - 0.721)	0.152	0.720	(0.719 - 0.721)	0.152
	5	0.728	(0.727 - 0.729)	0.159	0.728	(0.727 - 0.729)	0.159
	6	0.732	(0.731 - 0.732)	0.162	0.731	(0.731 - 0.732)	0.162
	7	0.733	(0.732 - 0.734)	0.162	0.733	(0.732 - 0.734)	0.163
	8	0.732	(0.731 - 0.733)	0.161	0.732	(0.731 - 0.733)	0.162
	9	0.731	(0.730 - 0.732)	0.159	0.731	(0.730 - 0.732)	0.160
	10	0.729	(0.728 - 0.730)	0.157	0.729	(0.729 - 0.730)	0.158
	11	0.726	(0.725 - 0.727)	0.154	0.727	(0.727 - 0.728)	0.156
	12	0.724	(0.723 - 0.725)	0.152	0.726	(0.725 - 0.727)	0.154
	13	0.721	(0.720 - 0.722)	0.149	0.723	(0.723 - 0.724)	0.152
	14	0.719	(0.718 - 0.719)	0.146	0.721	(0.720 - 0.722)	0.150
	15	0.716	(0.715 - 0.717)	0.144	0.719	(0.718 - 0.720)	0.148
	16	0.713	(0.713 - 0.714)	0.141	0.717	(0.716 - 0.718)	0.146
	17	0.711	(0.710 - 0.712)	0.139	0.715	(0.714 - 0.716)	0.144
	18	0.709	(0.708 - 0.709)	0.136	0.713	(0.712 - 0.714)	0.142
	19	0.706	(0.705 - 0.707)	0.134	0.711	(0.710 - 0.712)	0.140
	20	0.704	(0.703 - 0.705)	0.132	0.709	(0.708 - 0.710)	0.138

Abbreviations: MM2+: multimorbidity defined as ≥2 chronic conditions; MM3+: multimorbidity defined as ≥3 chronic conditions; MM4+: multimorbidity defined as ≥4 chronic conditions; na: not applicable

a The maximum length of lookback period is 5 years for: depression and mood diseases; Neurotic, stress-related and somatoform diseases; Sleep disorders.

b Shaded areas indicate the length of lookback period where the c-statistic ranged in the standard error interval [±0.001] of the maximal c-statistic.

c The baseline model includes only the covariates (age group, sex, material and social deprivation)

Table A2.3.1: Predictive performance (c-statistic) and model adjustment (Brier scaled score) to predict 1-year mortality for the Core list (L20) by criterion used to define multimorbidity, length of lookback period, and type of case definition (unique case definition for all diseases, case definition specific for some diseases with/without hypertension)

Multimorbidity definition	Lookback period (year)	Unique case definition			Validated case definition[a]			Validated case definition with hypertension[b]		
		c-statistic (95% IC)[c]		Brier scaled score	c-statistic (95% IC)[c]		Brier scaled score	c-statistic (95% IC)[c]		Brier scaled score
Baseline model[d]	na	0.758	(0.756 - 0.760)	0.048	0.758	(0.756 - 0.760)	0.048	0.758	(0.756 - 0.760)	0.048
MM2+	1	0.799	(0.797 - 0.801)	0.063	0.798	(0.796 - 0.800)	0.061	0.796	(0.794 - 0.798)	0.058
	2	0.800	(0.798 - 0.802)	0.062	0.800	(0.798 - 0.802)	0.061	0.795	(0.793 - 0.797)	0.057
	3	0.799	(0.797 - 0.800)	0.061	0.800	(0.798 - 0.802)	0.061	0.793	(0.792 - 0.795)	0.057
	4	0.797	(0.795 - 0.798)	0.061	0.799	(0.797 - 0.801)	0.061	0.791	(0.789 - 0.793)	0.056
	5	0.794	(0.792 - 0.796)	0.060	0.798	(0.796 - 0.799)	0.061	0.789	(0.787 - 0.791)	0.056
	6	0.792	(0.790 - 0.794)	0.059	0.797	(0.795 - 0.799)	0.061	0.787	(0.785 - 0.789)	0.056
	7	0.791	(0.789 - 0.793)	0.059	0.796	(0.794 - 0.798)	0.060	0.786	(0.784 - 0.788)	0.055
	8	0.789	(0.787 - 0.791)	0.058	0.795	(0.793 - 0.797)	0.060	0.785	(0.783 - 0.786)	0.055
	9	0.787	(0.785 - 0.789)	0.057	0.794	(0.792 - 0.796)	0.059	0.784	(0.782 - 0.785)	0.055
	10	0.786	(0.784 - 0.788)	0.057	0.793	(0.791 - 0.795)	0.059	0.783	(0.781 - 0.785)	0.055
	11	0.785	(0.783 - 0.786)	0.056	0.793	(0.791 - 0.794)	0.059	0.782	(0.780 - 0.784)	0.054
	12	0.783	(0.781 - 0.785)	0.056	0.792	(0.790 - 0.794)	0.059	0.781	(0.779 - 0.783)	0.054
	13	0.782	(0.780 - 0.784)	0.056	0.791	(0.789 - 0.793)	0.058	0.781	(0.779 - 0.783)	0.054
	14	0.782	(0.780 - 0.784)	0.055	0.791	(0.789 - 0.792)	0.058	0.780	(0.778 - 0.782)	0.054
	15	0.781	(0.779 - 0.783)	0.055	0.790	(0.788 - 0.792)	0.058	0.779	(0.777 - 0.781)	0.054
	16	0.780	(0.778 - 0.782)	0.055	0.789	(0.788 - 0.791)	0.057	0.779	(0.777 - 0.781)	0.053
	17	0.779	(0.777 - 0.781)	0.054	0.789	(0.787 - 0.791)	0.057	0.778	(0.776 - 0.780)	0.053
	18	0.778	(0.776 - 0.780)	0.054	0.789	(0.787 - 0.790)	0.057	0.778	(0.776 - 0.780)	0.053
	19	0.778	(0.776 - 0.780)	0.054	0.788	(0.786 - 0.790)	0.057	0.778	(0.776 - 0.780)	0.053
	20	0.777	(0.775 - 0.779)	0.054	0.788	(0.786 - 0.790)	0.057	0.778	(0.776 - 0.780)	0.053
MM3+	1	0.793	(0.791 - 0.795)	0.065	0.793	(0.791 - 0.795)	0.064	0.796	(0.794 - 0.798)	0.063
	2	0.799	(0.797 - 0.801)	0.066	0.798	(0.796 - 0.800)	0.065	0.801	(0.799 - 0.802)	0.063
	3	0.800	(0.798 - 0.802)	0.066	0.801	(0.799 - 0.803)	0.065	0.801	(0.799 - 0.803)	0.063
	4	0.801	(0.799 - 0.803)	0.066	0.802	(0.800 - 0.804)	0.066	0.801	(0.799 - 0.803)	0.063
	5	0.800	(0.798 - 0.802)	0.065	0.802	(0.800 - 0.804)	0.066	0.800	(0.798 - 0.802)	0.062
	6	0.800	(0.798 - 0.801)	0.065	0.802	(0.800 - 0.804)	0.066	0.799	(0.797 - 0.801)	0.062
	7	0.798	(0.797 - 0.800)	0.064	0.802	(0.800 - 0.804)	0.066	0.798	(0.796 - 0.800)	0.062
	8	0.798	(0.796 - 0.799)	0.063	0.802	(0.800 - 0.803)	0.065	0.797	(0.795 - 0.799)	0.061
	9	0.796	(0.794 - 0.798)	0.063	0.801	(0.799 - 0.803)	0.065	0.796	(0.794 - 0.798)	0.061
	10	0.795	(0.794 - 0.797)	0.062	0.801	(0.799 - 0.802)	0.065	0.795	(0.793 - 0.797)	0.060
	11	0.795	(0.793 - 0.796)	0.062	0.800	(0.799 - 0.802)	0.065	0.794	(0.793 - 0.796)	0.060

Multimorbidity definition	Lookback period (year)	Unique case definition			Validated case definition[a]			Validated case definition with hypertension[b]		
		c-statistic (95% IC)[c]		Brier scaled score	c-statistic (95% IC)[c]		Brier scaled score	c-statistic (95% IC)[c]		Brier scaled score
	12	0.794	(0.792 - 0.795)	0.061	0.800	(0.798 - 0.802)	0.064	0.794	(0.792 - 0.796)	0.060
	13	0.793	(0.791 - 0.795)	0.061	0.800	(0.798 - 0.802)	0.064	0.793	(0.791 - 0.795)	0.059
	14	0.792	(0.790 - 0.794)	0.060	0.799	(0.798 - 0.801)	0.064	0.793	(0.791 - 0.795)	0.059
	15	0.791	(0.789 - 0.793)	0.060	0.799	(0.797 - 0.801)	0.063	0.792	(0.790 - 0.794)	0.059
	16	0.790	(0.788 - 0.792)	0.059	0.799	(0.797 - 0.801)	0.063	0.792	(0.790 - 0.794)	0.059
	17	0.789	(0.787 - 0.791)	0.059	0.798	(0.797 - 0.800)	0.063	0.791	(0.789 - 0.793)	0.058
	18	0.789	(0.787 - 0.790)	0.059	0.798	(0.796 - 0.800)	0.063	0.791	(0.789 - 0.793)	0.058
	19	0.788	(0.786 - 0.790)	0.058	0.798	(0.796 - 0.800)	0.063	0.791	(0.789 - 0.793)	0.058
	20	0.787	(0.785 - 0.789)	0.058	0.798	(0.796 - 0.800)	0.062	0.790	(0.788 - 0.792)	0.058
MM4+	1	0.783	(0.781 - 0.785)	0.063	0.783	(0.781 - 0.785)	0.063	0.789	(0.787 - 0.791)	0.064
	2	0.791	(0.788 - 0.793)	0.065	0.790	(0.788 - 0.792)	0.065	0.796	(0.794 - 0.798)	0.065
	3	0.794	(0.792 - 0.796)	0.066	0.793	(0.791 - 0.795)	0.066	0.798	(0.796 - 0.800)	0.066
	4	0.796	(0.794 - 0.798)	0.066	0.795	(0.793 - 0.797)	0.066	0.800	(0.798 - 0.802)	0.066
	5	0.797	(0.795 - 0.799)	0.066	0.797	(0.795 - 0.799)	0.067	0.800	(0.798 - 0.802)	0.066
	6	0.798	(0.796 - 0.800)	0.067	0.798	(0.796 - 0.800)	0.067	0.800	(0.798 - 0.802)	0.066
	7	0.798	(0.796 - 0.800)	0.067	0.798	(0.796 - 0.800)	0.067	0.800	(0.799 - 0.802)	0.066
	8	0.798	(0.796 - 0.800)	0.066	0.799	(0.797 - 0.801)	0.067	0.800	(0.798 - 0.802)	0.065
	9	0.797	(0.795 - 0.799)	0.066	0.799	(0.797 - 0.801)	0.067	0.799	(0.797 - 0.801)	0.065
	10	0.797	(0.795 - 0.799)	0.066	0.799	(0.797 - 0.801)	0.067	0.799	(0.797 - 0.801)	0.065
	11	0.797	(0.795 - 0.799)	0.065	0.799	(0.797 - 0.801)	0.067	0.799	(0.797 - 0.801)	0.065
	12	0.797	(0.795 - 0.799)	0.065	0.799	(0.797 - 0.801)	0.067	0.799	(0.797 - 0.801)	0.064
	13	0.796	(0.794 - 0.798)	0.064	0.799	(0.797 - 0.801)	0.067	0.799	(0.797 - 0.800)	0.064
	14	0.796	(0.794 - 0.798)	0.064	0.799	(0.797 - 0.801)	0.067	0.798	(0.796 - 0.800)	0.064
	15	0.795	(0.793 - 0.797)	0.064	0.799	(0.797 - 0.801)	0.066	0.798	(0.796 - 0.800)	0.064
	16	0.795	(0.793 - 0.797)	0.063	0.799	(0.797 - 0.801)	0.066	0.798	(0.796 - 0.800)	0.063
	17	0.794	(0.792 - 0.796)	0.063	0.799	(0.797 - 0.801)	0.066	0.798	(0.796 - 0.799)	0.063
	18	0.794	(0.792 - 0.796)	0.063	0.799	(0.797 - 0.801)	0.066	0.797	(0.795 - 0.799)	0.063
	19	0.794	(0.792 - 0.795)	0.062	0.799	(0.797 - 0.801)	0.066	0.797	(0.795 - 0.799)	0.063
	20	0.793	(0.791 - 0.795)	0.062	0.798	(0.797 - 0.800)	0.066	0.797	(0.795 - 0.799)	0.063

Abbreviations: MM2+: multimorbidity defined as ≥2 chronic conditions; MM3+: multimorbidity defined as ≥3 chronic conditions; MM4+: multimorbidity defined as ≥4 chronic conditions; na: not applicable

a All case definitions are validated and available in Table A1.3

b All case definitions are validated and available in Table A1.3. Hypertension has been added to the Core list.

c Shaded areas indicate the length of lookback period where the c-statistic ranged in the standard error interval [±0.001] of the maximal c-statistic.

d The baseline model includes only the covariates (age group, sex, material and social deprivation)

Table A2.3.2: Predictive performance (c-statistic) and model adjustment (Brier scaled score) to predict hospitalisation (≥1/year) for the Core list (L20) by criterion used to define multimorbidity, length of lookback period, and type of case definition (unique case definition for all diseases, case definition specific for some diseases with/without hypertension)

Multimorbidity definition	Lookback period (year)	Unique case definition			Validated case definition[a]			Validated case definition with hypertension[b]		
		c-statistic (95% IC)[c]		Brier scaled score	c-statistic (95% IC)[c]		Brier scaled score	c-statistic (95% IC)[c]		Brier scaled score
Baseline model[d]	na	0.615	(0.614 - 0.617)	0.019	0.615	(0.614 - 0.617)	0.019	0.615	(0.614 - 0.617)	0.019
MM2+	1	0.662	(0.660 - 0.663)	0.046	0.663	(0.661 - 0.664)	0.044	0.666	(0.664 - 0.667)	0.043
	2	0.673	(0.672 - 0.675)	0.047	0.673	(0.671 - 0.674)	0.045	0.673	(0.671 - 0.674)	0.043
	3	0.676	(0.675 - 0.678)	0.045	0.675	(0.674 - 0.677)	0.044	0.673	(0.671 - 0.674)	0.041
	4	0.675	(0.674 - 0.677)	0.043	0.675	(0.674 - 0.676)	0.043	0.671	(0.669 - 0.672)	0.039
	5	0.674	(0.673 - 0.675)	0.042	0.674	(0.673 - 0.675)	0.042	0.668	(0.666 - 0.669)	0.038
	6	0.672	(0.671 - 0.674)	0.040	0.673	(0.672 - 0.675)	0.041	0.666	(0.664 - 0.667)	0.036
	7	0.671	(0.669 - 0.672)	0.039	0.673	(0.671 - 0.674)	0.040	0.664	(0.663 - 0.666)	0.036
	8	0.669	(0.668 - 0.670)	0.038	0.672	(0.671 - 0.673)	0.040	0.662	(0.661 - 0.664)	0.035
	9	0.667	(0.666 - 0.669)	0.037	0.671	(0.670 - 0.673)	0.039	0.661	(0.660 - 0.663)	0.034
	10	0.666	(0.664 - 0.667)	0.036	0.671	(0.669 - 0.672)	0.039	0.660	(0.658 - 0.661)	0.034
	11	0.664	(0.663 - 0.666)	0.036	0.670	(0.669 - 0.672)	0.039	0.659	(0.658 - 0.660)	0.033
	12	0.662	(0.661 - 0.664)	0.035	0.669	(0.668 - 0.671)	0.038	0.658	(0.656 - 0.659)	0.033
	13	0.661	(0.659 - 0.662)	0.034	0.668	(0.667 - 0.670)	0.038	0.657	(0.655 - 0.658)	0.032
	14	0.660	(0.658 - 0.661)	0.034	0.668	(0.666 - 0.669)	0.037	0.656	(0.655 - 0.657)	0.032
	15	0.658	(0.657 - 0.660)	0.033	0.667	(0.666 - 0.669)	0.037	0.655	(0.654 - 0.657)	0.032
	16	0.657	(0.655 - 0.658)	0.032	0.667	(0.665 - 0.668)	0.037	0.654	(0.653 - 0.656)	0.031
	17	0.656	(0.654 - 0.657)	0.032	0.666	(0.665 - 0.668)	0.036	0.654	(0.653 - 0.655)	0.031
	18	0.655	(0.653 - 0.656)	0.031	0.666	(0.664 - 0.667)	0.036	0.654	(0.652 - 0.655)	0.031
	19	0.653	(0.652 - 0.655)	0.031	0.665	(0.664 - 0.667)	0.036	0.653	(0.652 - 0.655)	0.031
	20	0.653	(0.651 - 0.654)	0.031	0.665	(0.663 - 0.666)	0.036	0.653	(0.651 - 0.654)	0.031
MM3+	1	0.647	(0.646 - 0.649)	0.043	0.649	(0.647 - 0.650)	0.043	0.656	(0.654 - 0.657)	0.045
	2	0.662	(0.660 - 0.663)	0.048	0.663	(0.661 - 0.664)	0.047	0.670	(0.668 - 0.671)	0.048
	3	0.669	(0.667 - 0.670)	0.049	0.669	(0.667 - 0.670)	0.048	0.675	(0.673 - 0.676)	0.048
	4	0.672	(0.671 - 0.674)	0.048	0.672	(0.671 - 0.674)	0.048	0.677	(0.675 - 0.678)	0.047
	5	0.674	(0.673 - 0.676)	0.047	0.674	(0.672 - 0.675)	0.047	0.677	(0.675 - 0.678)	0.046
	6	0.675	(0.674 - 0.677)	0.047	0.675	(0.673 - 0.676)	0.047	0.677	(0.676 - 0.679)	0.045
	7	0.676	(0.674 - 0.677)	0.046	0.676	(0.674 - 0.677)	0.047	0.677	(0.675 - 0.678)	0.044
	8	0.676	(0.674 - 0.677)	0.045	0.676	(0.675 - 0.678)	0.046	0.676	(0.675 - 0.678)	0.043
	9	0.675	(0.674 - 0.677)	0.044	0.676	(0.675 - 0.678)	0.046	0.676	(0.674 - 0.677)	0.043
	10	0.675	(0.673 - 0.676)	0.043	0.677	(0.675 - 0.678)	0.046	0.675	(0.674 - 0.677)	0.042
	11	0.674	(0.673 - 0.675)	0.042	0.677	(0.675 - 0.678)	0.046	0.675	(0.673 - 0.676)	0.042
	12	0.673	(0.672 - 0.675)	0.042	0.677	(0.675 - 0.678)	0.045	0.674	(0.673 - 0.676)	0.041
	13	0.672	(0.671 - 0.674)	0.041	0.676	(0.675 - 0.678)	0.045	0.673	(0.672 - 0.675)	0.041

Multimorbidity definition	Lookback period (year)	Unique case definition			Validated case definition[a]			Validated case definition with hypertension[b]		
		c-statistic (95% IC)[c]		Brier scaled score	c-statistic (95% IC)[c]		Brier scaled score	c-statistic (95% IC)[c]		Brier scaled score
	14	0.672	(0.670 - 0.673)	0.041	0.676	(0.675 - 0.678)	0.045	0.673	(0.671 - 0.674)	0.040
	15	0.671	(0.669 - 0.672)	0.040	0.676	(0.675 - 0.678)	0.044	0.672	(0.671 - 0.674)	0.040
	16	0.670	(0.668 - 0.671)	0.039	0.676	(0.675 - 0.678)	0.044	0.672	(0.670 - 0.673)	0.040
	17	0.669	(0.668 - 0.671)	0.039	0.676	(0.675 - 0.677)	0.044	0.671	(0.670 - 0.673)	0.039
	18	0.668	(0.667 - 0.670)	0.038	0.676	(0.674 - 0.677)	0.044	0.671	(0.669 - 0.672)	0.039
	19	0.668	(0.666 - 0.669)	0.038	0.676	(0.674 - 0.677)	0.043	0.670	(0.669 - 0.672)	0.039
	20	0.667	(0.666 - 0.668)	0.037	0.676	(0.674 - 0.677)	0.043	0.670	(0.669 - 0.672)	0.039
MM4+	1	0.635	(0.634 - 0.637)	0.037	0.636	(0.634 - 0.637)	0.037	0.644	(0.643 - 0.646)	0.042
	2	0.646	(0.645 - 0.648)	0.043	0.647	(0.645 - 0.648)	0.043	0.657	(0.656 - 0.659)	0.047
	3	0.654	(0.652 - 0.655)	0.045	0.653	(0.652 - 0.655)	0.045	0.664	(0.663 - 0.666)	0.048
	4	0.659	(0.657 - 0.660)	0.046	0.658	(0.656 - 0.659)	0.046	0.669	(0.667 - 0.670)	0.048
	5	0.662	(0.661 - 0.664)	0.047	0.661	(0.660 - 0.663)	0.046	0.671	(0.670 - 0.673)	0.048
	6	0.665	(0.664 - 0.667)	0.047	0.663	(0.662 - 0.665)	0.047	0.673	(0.671 - 0.674)	0.048
	7	0.667	(0.665 - 0.669)	0.047	0.665	(0.663 - 0.666)	0.047	0.674	(0.672 - 0.675)	0.048
	8	0.668	(0.667 - 0.670)	0.047	0.666	(0.664 - 0.667)	0.047	0.675	(0.673 - 0.676)	0.048
	9	0.669	(0.668 - 0.671)	0.046	0.667	(0.665 - 0.669)	0.047	0.675	(0.674 - 0.677)	0.047
	10	0.670	(0.669 - 0.672)	0.046	0.668	(0.666 - 0.669)	0.047	0.676	(0.674 - 0.677)	0.047
	11	0.671	(0.670 - 0.673)	0.046	0.669	(0.667 - 0.670)	0.047	0.676	(0.675 - 0.678)	0.047
	12	0.671	(0.670 - 0.673)	0.045	0.669	(0.668 - 0.671)	0.047	0.676	(0.675 - 0.678)	0.047
	13	0.672	(0.670 - 0.673)	0.045	0.670	(0.668 - 0.671)	0.047	0.676	(0.675 - 0.678)	0.046
	14	0.672	(0.670 - 0.673)	0.044	0.670	(0.669 - 0.672)	0.047	0.676	(0.675 - 0.678)	0.046
	15	0.672	(0.670 - 0.673)	0.044	0.671	(0.669 - 0.672)	0.047	0.676	(0.675 - 0.678)	0.046
	16	0.672	(0.670 - 0.673)	0.044	0.671	(0.669 - 0.672)	0.047	0.676	(0.675 - 0.678)	0.046
	17	0.671	(0.670 - 0.673)	0.043	0.671	(0.670 - 0.673)	0.047	0.676	(0.675 - 0.678)	0.045
	18	0.671	(0.670 - 0.673)	0.043	0.671	(0.670 - 0.673)	0.046	0.676	(0.675 - 0.678)	0.045
	19	0.671	(0.670 - 0.673)	0.042	0.672	(0.670 - 0.673)	0.046	0.676	(0.675 - 0.678)	0.045
	20	0.671	(0.670 - 0.673)	0.042	0.672	(0.671 - 0.674)	0.046	0.676	(0.675 - 0.678)	0.045

Abbreviations: MM2+: multimorbidity defined as ≥2 chronic conditions; MM3+: multimorbidity defined as ≥3 chronic conditions; MM4+: multimorbidity defined as ≥4 chronic conditions; na: not applicable

a All case definitions are validated and available in Table A1.3

b All case definitions are validated and available in Table A1.3. Hypertension has been added to the Core list.

c Shaded areas indicate the length of lookback period where the c-statistic ranged in the standard error interval [±0.001] of the maximal c-statistic.

d The baseline model includes only the covariates (age group, sex, material and social deprivation)

Table A2.3.3: Predictive performance (c-statistic) and model adjustment (Brier scaled score) to predict <u>frequent visits to emergency department (≥3/year) for the Core list (L20)</u> by criterion used to define multimorbidity, length of lookback periodand type of case definition (unique case definition for all diseases, case definition specific for some diseases with/without hypertension)

Multimorbidity definition	Lookback period (year)	Unique case definition		Validated case definition[a]		Validated case definition with hypertension[b]	
		c-statistic (95% IC)[c]	Brier scaled score	c-statistic (95% IC)[c]	Brier scaled score	c-statistic (95% IC)[c]	Brier scaled score
Baseline model[d]	na	0.634 (0.632 - 0.636)	0.011	0.634 (0.632 - 0.636)	0.011	0.634 (0.632 - 0.636)	0.011
MM2+	1	0.692 (0.690 - 0.695)	0.031	0.693 (0.691 - 0.696)	0.029	0.695 (0.693 - 0.697)	0.028
	2	0.705 (0.703 - 0.707)	0.031	0.705 (0.702 - 0.707)	0.029	0.703 (0.701 - 0.705)	0.027
	3	0.710 (0.707 - 0.712)	0.030	0.708 (0.706 - 0.710)	0.028	0.703 (0.701 - 0.706)	0.026
	4	0.710 (0.708 - 0.712)	0.028	0.708 (0.706 - 0.710)	0.027	0.701 (0.699 - 0.703)	0.024
	5	0.708 (0.706 - 0.710)	0.027	0.706 (0.704 - 0.708)	0.026	0.697 (0.695 - 0.699)	0.023
	6	0.707 (0.705 - 0.709)	0.026	0.706 (0.704 - 0.708)	0.026	0.694 (0.692 - 0.696)	0.022
	7	0.705 (0.703 - 0.707)	0.025	0.705 (0.702 - 0.707)	0.025	0.692 (0.690 - 0.694)	0.021
	8	0.703 (0.701 - 0.705)	0.024	0.703 (0.701 - 0.706)	0.025	0.690 (0.688 - 0.692)	0.021
	9	0.700 (0.698 - 0.702)	0.023	0.702 (0.700 - 0.704)	0.024	0.688 (0.686 - 0.690)	0.020
	10	0.698 (0.696 - 0.700)	0.023	0.701 (0.699 - 0.703)	0.024	0.686 (0.684 - 0.688)	0.020
	11	0.696 (0.694 - 0.698)	0.022	0.700 (0.698 - 0.702)	0.023	0.685 (0.682 - 0.687)	0.019
	12	0.694 (0.692 - 0.696)	0.021	0.699 (0.697 - 0.701)	0.023	0.683 (0.681 - 0.685)	0.019
	13	0.692 (0.690 - 0.694)	0.021	0.698 (0.696 - 0.700)	0.023	0.682 (0.680 - 0.684)	0.019
	14	0.691 (0.689 - 0.693)	0.021	0.697 (0.695 - 0.699)	0.022	0.681 (0.679 - 0.683)	0.018
	15	0.689 (0.687 - 0.691)	0.020	0.696 (0.694 - 0.699)	0.022	0.680 (0.678 - 0.682)	0.018
	16	0.687 (0.685 - 0.689)	0.020	0.696 (0.694 - 0.698)	0.022	0.679 (0.677 - 0.681)	0.018
	17	0.686 (0.683 - 0.688)	0.019	0.695 (0.693 - 0.697)	0.022	0.678 (0.676 - 0.680)	0.018
	18	0.684 (0.682 - 0.686)	0.019	0.695 (0.693 - 0.697)	0.022	0.677 (0.675 - 0.680)	0.018
	19	0.683 (0.680 - 0.685)	0.019	0.694 (0.692 - 0.696)	0.021	0.677 (0.675 - 0.679)	0.018
	20	0.681 (0.679 - 0.683)	0.019	0.693 (0.691 - 0.696)	0.021	0.676 (0.674 - 0.678)	0.017
MM3+	1	0.678 (0.675 - 0.680)	0.031	0.680 (0.677 - 0.682)	0.031	0.687 (0.684 - 0.689)	0.032
	2	0.696 (0.693 - 0.698)	0.034	0.696 (0.694 - 0.699)	0.033	0.702 (0.700 - 0.705)	0.033
	3	0.705 (0.702 - 0.707)	0.035	0.704 (0.702 - 0.707)	0.034	0.709 (0.706 - 0.711)	0.032
	4	0.710 (0.707 - 0.712)	0.034	0.708 (0.706 - 0.711)	0.033	0.711 (0.709 - 0.713)	0.031
	5	0.712 (0.710 - 0.714)	0.033	0.710 (0.708 - 0.712)	0.032	0.711 (0.709 - 0.713)	0.030
	6	0.713 (0.711 - 0.715)	0.032	0.711 (0.709 - 0.714)	0.032	0.711 (0.709 - 0.713)	0.029
	7	0.713 (0.711 - 0.716)	0.031	0.712 (0.710 - 0.714)	0.031	0.710 (0.708 - 0.712)	0.028
	8	0.714 (0.711 - 0.716)	0.031	0.713 (0.710 - 0.715)	0.031	0.709 (0.707 - 0.711)	0.028
	9	0.713 (0.711 - 0.716)	0.030	0.713 (0.711 - 0.715)	0.031	0.708 (0.706 - 0.710)	0.027
	10	0.713 (0.711 - 0.715)	0.029	0.713 (0.711 - 0.715)	0.031	0.708 (0.705 - 0.710)	0.026
	11	0.712 (0.710 - 0.714)	0.029	0.713 (0.711 - 0.715)	0.030	0.706 (0.704 - 0.708)	0.026
	12	0.711 (0.709 - 0.713)	0.028	0.713 (0.711 - 0.715)	0.030	0.706 (0.703 - 0.708)	0.025
	13	0.710 (0.708 - 0.712)	0.027	0.713 (0.711 - 0.715)	0.030	0.705 (0.703 - 0.707)	0.025

Multimorbidity definition	Lookback period (year)	Unique case definition			Validated case definition[a]			Validated case definition with hypertension[b]		
		c-statistic (95% IC)[c]		Brier scaled score	c-statistic (95% IC)[c]		Brier scaled score	c-statistic (95% IC)[c]		Brier scaled score
	14	0.709	(0.707 - 0.712)	0.027	0.713	(0.711 - 0.715)	0.030	0.704	(0.702 - 0.706)	0.025
	15	0.708	(0.706 - 0.710)	0.026	0.713	(0.711 - 0.715)	0.029	0.703	(0.701 - 0.705)	0.024
	16	0.707	(0.705 - 0.709)	0.026	0.713	(0.711 - 0.715)	0.029	0.703	(0.701 - 0.705)	0.024
	17	0.706	(0.704 - 0.708)	0.025	0.713	(0.710 - 0.715)	0.029	0.702	(0.700 - 0.704)	0.024
	18	0.705	(0.703 - 0.707)	0.025	0.712	(0.710 - 0.714)	0.029	0.702	(0.700 - 0.704)	0.024
	19	0.704	(0.702 - 0.706)	0.025	0.712	(0.710 - 0.714)	0.029	0.701	(0.699 - 0.703)	0.024
	20	0.703	(0.701 - 0.705)	0.024	0.712	(0.710 - 0.714)	0.028	0.701	(0.699 - 0.703)	0.024
MM4+	1	0.663	(0.660 - 0.665)	0.027	0.664	(0.661 - 0.666)	0.027	0.674	(0.672 - 0.676)	0.030
	2	0.679	(0.676 - 0.681)	0.032	0.679	(0.677 - 0.681)	0.032	0.691	(0.688 - 0.693)	0.034
	3	0.688	(0.686 - 0.691)	0.034	0.688	(0.685 - 0.690)	0.034	0.699	(0.696 - 0.701)	0.035
	4	0.695	(0.693 - 0.697)	0.034	0.693	(0.691 - 0.695)	0.034	0.704	(0.702 - 0.706)	0.034
	5	0.700	(0.698 - 0.703)	0.035	0.698	(0.695 - 0.700)	0.034	0.707	(0.705 - 0.709)	0.034
	6	0.704	(0.701 - 0.706)	0.034	0.700	(0.698 - 0.702)	0.034	0.709	(0.707 - 0.711)	0.033
	7	0.706	(0.704 - 0.709)	0.034	0.702	(0.700 - 0.705)	0.034	0.710	(0.708 - 0.712)	0.033
	8	0.708	(0.706 - 0.711)	0.034	0.704	(0.702 - 0.706)	0.034	0.711	(0.709 - 0.713)	0.033
	9	0.710	(0.707 - 0.712)	0.034	0.705	(0.703 - 0.708)	0.034	0.712	(0.710 - 0.714)	0.032
	10	0.711	(0.709 - 0.713)	0.034	0.706	(0.704 - 0.708)	0.034	0.712	(0.710 - 0.714)	0.032
	11	0.712	(0.709 - 0.714)	0.033	0.707	(0.705 - 0.709)	0.034	0.712	(0.710 - 0.715)	0.032
	12	0.712	(0.710 - 0.714)	0.033	0.708	(0.706 - 0.710)	0.034	0.713	(0.710 - 0.715)	0.031
	13	0.713	(0.710 - 0.715)	0.032	0.709	(0.706 - 0.711)	0.034	0.713	(0.711 - 0.715)	0.031
	14	0.713	(0.711 - 0.715)	0.032	0.709	(0.707 - 0.712)	0.034	0.713	(0.711 - 0.715)	0.031
	15	0.713	(0.711 - 0.715)	0.032	0.710	(0.708 - 0.712)	0.033	0.713	(0.711 - 0.715)	0.031
	16	0.713	(0.711 - 0.715)	0.031	0.710	(0.708 - 0.713)	0.033	0.713	(0.710 - 0.715)	0.030
	17	0.713	(0.711 - 0.715)	0.031	0.711	(0.709 - 0.713)	0.033	0.713	(0.710 - 0.715)	0.030
	18	0.713	(0.711 - 0.715)	0.030	0.711	(0.709 - 0.713)	0.033	0.712	(0.710 - 0.715)	0.030
	19	0.712	(0.710 - 0.714)	0.030	0.711	(0.709 - 0.714)	0.033	0.712	(0.710 - 0.715)	0.030
	20	0.712	(0.710 - 0.714)	0.030	0.712	(0.709 - 0.714)	0.033	0.712	(0.710 - 0.714)	0.030

Abbreviations: MM2+: multimorbidity defined as ≥2 chronic conditions; MM3+: multimorbidity defined as ≥3 chronic conditions; MM4+: multimorbidity defined as ≥4 chronic conditions; na: not applicable

a All case definitions are validated and available in Table A1.3

b All case definitions are validated and available in Table A1.3. Hypertension has been added to the Core list.

c Shaded areas indicate the length of lookback period where the c-statistic ranged in the standard error interval [±0.001] of the maximal c-statistic.

d The baseline model includes only the covariates (age group, sex, material and social deprivation)

Table A2.3.4: Predictive performance (c-statistic) and model adjustment (Brier scaled score) to predict frequent visits to general practitioner (≥7/year) for the Core list (L20) by criterion used to define multimorbidity, length of lookback period, and type of case definition (unique case definition for all diseases, case definition specific for some diseases with/without hypertension)

Multimorbidity definition	Lookback period (year)	Unique case definition			Validated case definition[a]			Validated case definition with hypertension[b]		
		c-statistic (95% IC)[c]		Brier scaled score	c-statistic (95% IC)[c]		Brier scaled score	c-statistic (95% IC)[c]		Brier scaled score
Baseline model[d]	na	0.629	(0.627 - 0.631)	0.020	0.629	(0.627 - 0.631)	0.020	0.629	(0.627 - 0.631)	0.020
MM2+	1	0.661	(0.659 - 0.663)	0.036	0.664	(0.663 - 0.666)	0.037	0.668	(0.666 - 0.669)	0.038
	2	0.677	(0.675 - 0.679)	0.041	0.679	(0.677 - 0.680)	0.041	0.681	(0.679 - 0.683)	0.040
	3	0.687	(0.685 - 0.688)	0.043	0.685	(0.683 - 0.686)	0.042	0.686	(0.684 - 0.687)	0.041
	4	0.692	(0.691 - 0.694)	0.044	0.688	(0.686 - 0.690)	0.042	0.687	(0.686 - 0.689)	0.040
	5	0.695	(0.693 - 0.696)	0.044	0.689	(0.687 - 0.691)	0.042	0.687	(0.685 - 0.688)	0.039
	6	0.696	(0.694 - 0.697)	0.044	0.689	(0.688 - 0.691)	0.041	0.686	(0.684 - 0.687)	0.038
	7	0.695	(0.694 - 0.697)	0.043	0.689	(0.687 - 0.691)	0.041	0.684	(0.683 - 0.686)	0.037
	8	0.695	(0.693 - 0.697)	0.042	0.689	(0.687 - 0.690)	0.040	0.683	(0.681 - 0.684)	0.036
	9	0.694	(0.693 - 0.696)	0.042	0.688	(0.687 - 0.690)	0.040	0.681	(0.680 - 0.683)	0.036
	10	0.693	(0.692 - 0.695)	0.041	0.688	(0.687 - 0.690)	0.040	0.680	(0.679 - 0.682)	0.035
	11	0.692	(0.691 - 0.694)	0.040	0.688	(0.687 - 0.690)	0.039	0.680	(0.678 - 0.681)	0.035
	12	0.691	(0.690 - 0.693)	0.040	0.688	(0.686 - 0.689)	0.039	0.679	(0.677 - 0.680)	0.034
	13	0.690	(0.689 - 0.692)	0.039	0.687	(0.686 - 0.689)	0.039	0.678	(0.676 - 0.679)	0.034
	14	0.689	(0.688 - 0.691)	0.039	0.687	(0.685 - 0.689)	0.038	0.677	(0.675 - 0.679)	0.034
	15	0.688	(0.686 - 0.689)	0.038	0.687	(0.685 - 0.688)	0.038	0.676	(0.675 - 0.678)	0.033
	16	0.686	(0.685 - 0.688)	0.037	0.687	(0.685 - 0.688)	0.038	0.676	(0.674 - 0.677)	0.033
	17	0.685	(0.684 - 0.687)	0.037	0.686	(0.685 - 0.688)	0.038	0.675	(0.674 - 0.677)	0.033
	18	0.684	(0.683 - 0.686)	0.037	0.686	(0.684 - 0.688)	0.038	0.675	(0.673 - 0.676)	0.033
	19	0.683	(0.682 - 0.685)	0.036	0.686	(0.684 - 0.687)	0.038	0.674	(0.673 - 0.676)	0.033
	20	0.682	(0.681 - 0.684)	0.036	0.686	(0.684 - 0.687)	0.037	0.674	(0.672 - 0.675)	0.032
MM3+	1	0.649	(0.647 - 0.650)	0.031	0.650	(0.649 - 0.652)	0.032	0.654	(0.653 - 0.656)	0.033
	2	0.661	(0.660 - 0.663)	0.036	0.663	(0.662 - 0.665)	0.037	0.669	(0.667 - 0.671)	0.039
	3	0.672	(0.670 - 0.674)	0.041	0.672	(0.670 - 0.673)	0.041	0.678	(0.676 - 0.680)	0.042
	4	0.680	(0.678 - 0.682)	0.043	0.677	(0.676 - 0.679)	0.042	0.684	(0.682 - 0.685)	0.043
	5	0.685	(0.684 - 0.687)	0.045	0.681	(0.680 - 0.683)	0.043	0.687	(0.685 - 0.688)	0.043
	6	0.689	(0.687 - 0.691)	0.046	0.684	(0.682 - 0.685)	0.044	0.688	(0.687 - 0.690)	0.043
	7	0.692	(0.690 - 0.693)	0.046	0.685	(0.683 - 0.686)	0.044	0.689	(0.688 - 0.691)	0.043
	8	0.694	(0.692 - 0.695)	0.046	0.686	(0.684 - 0.687)	0.044	0.689	(0.688 - 0.691)	0.042
	9	0.695	(0.694 - 0.697)	0.046	0.687	(0.685 - 0.688)	0.044	0.690	(0.688 - 0.692)	0.042
	10	0.697	(0.695 - 0.698)	0.046	0.688	(0.686 - 0.689)	0.044	0.690	(0.689 - 0.692)	0.042
	11	0.698	(0.696 - 0.699)	0.046	0.688	(0.687 - 0.690)	0.044	0.691	(0.689 - 0.692)	0.042
	12	0.698	(0.697 - 0.700)	0.046	0.689	(0.687 - 0.690)	0.044	0.690	(0.689 - 0.692)	0.042
	13	0.699	(0.697 - 0.700)	0.046	0.689	(0.688 - 0.691)	0.044	0.690	(0.689 - 0.692)	0.041

Multimorbidity definition	Lookback period (year)	Unique case definition			Validated case definition[a]			Validated case definition with hypertension[b]		
		c-statistic (95% IC)[c]		Brier scaled score	c-statistic (95% IC)[c]		Brier scaled score	c-statistic (95% IC)[c]		Brier scaled score
	14	0.699	(0.697 - 0.700)	0.045	0.690	(0.688 - 0.691)	0.044	0.690	(0.689 - 0.692)	0.041
	15	0.699	(0.697 - 0.700)	0.045	0.690	(0.689 - 0.692)	0.044	0.690	(0.689 - 0.692)	0.041
	16	0.699	(0.697 - 0.700)	0.045	0.691	(0.689 - 0.692)	0.044	0.690	(0.689 - 0.692)	0.041
	17	0.698	(0.697 - 0.700)	0.044	0.691	(0.689 - 0.693)	0.044	0.690	(0.688 - 0.692)	0.040
	18	0.698	(0.697 - 0.700)	0.044	0.691	(0.690 - 0.693)	0.044	0.690	(0.688 - 0.692)	0.040
	19	0.698	(0.696 - 0.699)	0.044	0.692	(0.690 - 0.693)	0.044	0.690	(0.688 - 0.691)	0.040
	20	0.697	(0.696 - 0.699)	0.043	0.692	(0.690 - 0.693)	0.044	0.690	(0.688 - 0.691)	0.040
MM4+	1	0.641	(0.640 - 0.643)	0.028	0.642	(0.640 - 0.644)	0.028	0.646	(0.645 - 0.648)	0.030
	2	0.649	(0.648 - 0.651)	0.032	0.650	(0.649 - 0.652)	0.033	0.657	(0.655 - 0.658)	0.035
	3	0.657	(0.655 - 0.659)	0.036	0.657	(0.655 - 0.659)	0.036	0.665	(0.663 - 0.666)	0.038
	4	0.663	(0.662 - 0.665)	0.039	0.662	(0.660 - 0.664)	0.038	0.671	(0.669 - 0.672)	0.041
	5	0.669	(0.667 - 0.670)	0.041	0.666	(0.664 - 0.667)	0.040	0.675	(0.674 - 0.677)	0.042
	6	0.673	(0.671 - 0.675)	0.042	0.668	(0.666 - 0.670)	0.041	0.678	(0.676 - 0.680)	0.043
	7	0.677	(0.675 - 0.678)	0.044	0.670	(0.668 - 0.672)	0.041	0.680	(0.678 - 0.681)	0.043
	8	0.679	(0.678 - 0.681)	0.044	0.672	(0.670 - 0.673)	0.042	0.681	(0.679 - 0.683)	0.043
	9	0.682	(0.680 - 0.683)	0.045	0.673	(0.671 - 0.675)	0.042	0.682	(0.681 - 0.684)	0.043
	10	0.684	(0.682 - 0.686)	0.046	0.674	(0.673 - 0.676)	0.042	0.684	(0.682 - 0.686)	0.044
	11	0.686	(0.685 - 0.688)	0.046	0.676	(0.674 - 0.677)	0.043	0.685	(0.683 - 0.687)	0.044
	12	0.688	(0.686 - 0.689)	0.046	0.677	(0.675 - 0.678)	0.043	0.686	(0.684 - 0.687)	0.044
	13	0.689	(0.688 - 0.691)	0.046	0.677	(0.676 - 0.679)	0.043	0.686	(0.685 - 0.688)	0.044
	14	0.690	(0.689 - 0.692)	0.046	0.678	(0.677 - 0.680)	0.043	0.687	(0.685 - 0.689)	0.044
	15	0.692	(0.690 - 0.693)	0.047	0.679	(0.678 - 0.681)	0.044	0.688	(0.686 - 0.689)	0.044
	16	0.693	(0.691 - 0.694)	0.047	0.680	(0.678 - 0.682)	0.044	0.688	(0.687 - 0.690)	0.044
	17	0.693	(0.692 - 0.695)	0.047	0.681	(0.679 - 0.682)	0.044	0.689	(0.687 - 0.690)	0.044
	18	0.694	(0.692 - 0.696)	0.047	0.681	(0.680 - 0.683)	0.044	0.689	(0.688 - 0.691)	0.044
	19	0.694	(0.693 - 0.696)	0.047	0.682	(0.680 - 0.684)	0.044	0.690	(0.688 - 0.691)	0.044
	20	0.695	(0.693 - 0.696)	0.046	0.682	(0.681 - 0.684)	0.044	0.690	(0.688 - 0.691)	0.044

Abbreviations: MM2+: multimorbidity defined as ≥2 chronic conditions; MM3+: multimorbidity defined as ≥3 chronic conditions; MM4+: multimorbidity defined as ≥4 chronic conditions; na: not applicable

a All case definitions are validated and available in Table A1.3

b All case definitions are validated and available in Table A1.3. Hypertension has been added to the Core list.

c Shaded areas indicate the length of lookback period where the c-statistic ranged in the standard error interval [±0.001] of the maximal c-statistic.

d The baseline model includes only the covariates (age group, sex, material and social deprivation)

Table A2.3.5: Predictive performance (c-statistic) and model adjustment (Brier scaled score) to predict <u>frequent visits to specialist physician (≥10/year) for the Core list (L20)</u> by criterion used to define multimorbidity, length of lookback period, and type of case definition (unique case definition for all diseases, case definition specific for some diseases with/without hypertension)

Multimorbidity definition	Lookback period (year)	Unique case definition			Validated case definition[a]			Validated case definition with hypertension[b]		
		c-statistic (95% IC)[c]		Brier scaled score	c-statistic (95% IC)[c]		Brier scaled score	c-statistic (95% IC)[c]		Brier scaled score
Baseline model[d]	na	0.572	(0.570 - 0.573)	0.007	0.572	(0.570 - 0.573)	0.007	0.572	(0.570 - 0.573)	0.007
MM2+	1	0.634	(0.633 - 0.636)	0.045	0.640	(0.639 - 0.642)	0.044	0.647	(0.645 - 0.649)	0.046
	2	0.660	(0.659 - 0.662)	0.051	0.662	(0.660 - 0.663)	0.049	0.667	(0.665 - 0.668)	0.048
	3	0.671	(0.670 - 0.673)	0.051	0.670	(0.669 - 0.671)	0.049	0.672	(0.670 - 0.673)	0.046
	4	0.675	(0.673 - 0.676)	0.049	0.672	(0.671 - 0.674)	0.048	0.671	(0.670 - 0.672)	0.043
	5	0.675	(0.674 - 0.677)	0.047	0.673	(0.671 - 0.674)	0.046	0.668	(0.667 - 0.669)	0.040
	6	0.674	(0.673 - 0.675)	0.045	0.672	(0.671 - 0.674)	0.045	0.665	(0.663 - 0.666)	0.038
	7	0.672	(0.671 - 0.673)	0.043	0.672	(0.670 - 0.673)	0.043	0.662	(0.660 - 0.663)	0.036
	8	0.670	(0.668 - 0.671)	0.041	0.671	(0.669 - 0.672)	0.042	0.659	(0.657 - 0.660)	0.035
	9	0.667	(0.666 - 0.669)	0.039	0.670	(0.669 - 0.672)	0.042	0.656	(0.655 - 0.658)	0.034
	10	0.665	(0.664 - 0.667)	0.038	0.669	(0.668 - 0.671)	0.041	0.654	(0.653 - 0.655)	0.033
	11	0.663	(0.661 - 0.664)	0.037	0.668	(0.667 - 0.670)	0.040	0.652	(0.651 - 0.653)	0.032
	12	0.660	(0.659 - 0.662)	0.035	0.668	(0.666 - 0.669)	0.040	0.650	(0.649 - 0.651)	0.031
	13	0.658	(0.656 - 0.659)	0.034	0.667	(0.665 - 0.668)	0.039	0.648	(0.647 - 0.649)	0.030
	14	0.656	(0.654 - 0.657)	0.033	0.666	(0.664 - 0.667)	0.039	0.646	(0.645 - 0.648)	0.030
	15	0.653	(0.652 - 0.655)	0.032	0.665	(0.663 - 0.666)	0.038	0.645	(0.644 - 0.646)	0.029
	16	0.651	(0.650 - 0.653)	0.031	0.664	(0.663 - 0.666)	0.038	0.644	(0.643 - 0.645)	0.029
	17	0.650	(0.648 - 0.651)	0.031	0.664	(0.662 - 0.665)	0.037	0.643	(0.642 - 0.644)	0.028
	18	0.648	(0.646 - 0.649)	0.030	0.663	(0.662 - 0.664)	0.037	0.642	(0.641 - 0.643)	0.028
	19	0.646	(0.645 - 0.647)	0.029	0.662	(0.661 - 0.664)	0.036	0.641	(0.640 - 0.643)	0.028
	20	0.644	(0.643 - 0.646)	0.029	0.661	(0.660 - 0.663)	0.036	0.640	(0.639 - 0.642)	0.027
MM3+	1	0.606	(0.605 - 0.608)	0.030	0.610	(0.608 - 0.611)	0.032	0.620	(0.618 - 0.621)	0.036
	2	0.627	(0.626 - 0.629)	0.040	0.631	(0.629 - 0.632)	0.042	0.643	(0.641 - 0.644)	0.045
	3	0.642	(0.640 - 0.643)	0.046	0.642	(0.641 - 0.644)	0.045	0.655	(0.654 - 0.657)	0.048
	4	0.652	(0.651 - 0.654)	0.049	0.650	(0.648 - 0.651)	0.047	0.662	(0.661 - 0.664)	0.049
	5	0.659	(0.657 - 0.660)	0.050	0.655	(0.653 - 0.656)	0.047	0.666	(0.665 - 0.668)	0.048
	6	0.663	(0.662 - 0.665)	0.050	0.658	(0.656 - 0.659)	0.048	0.668	(0.667 - 0.670)	0.047
	7	0.666	(0.665 - 0.668)	0.049	0.660	(0.659 - 0.662)	0.048	0.669	(0.668 - 0.671)	0.047
	8	0.668	(0.667 - 0.670)	0.048	0.662	(0.661 - 0.664)	0.048	0.670	(0.669 - 0.671)	0.046
	9	0.669	(0.668 - 0.671)	0.047	0.664	(0.662 - 0.665)	0.048	0.670	(0.669 - 0.672)	0.045
	10	0.670	(0.668 - 0.671)	0.047	0.665	(0.663 - 0.666)	0.048	0.670	(0.669 - 0.672)	0.045
	11	0.670	(0.669 - 0.672)	0.046	0.666	(0.664 - 0.667)	0.048	0.670	(0.669 - 0.671)	0.044
	12	0.671	(0.669 - 0.672)	0.045	0.666	(0.665 - 0.668)	0.047	0.670	(0.668 - 0.671)	0.043
	13	0.670	(0.669 - 0.672)	0.044	0.667	(0.665 - 0.668)	0.047	0.669	(0.668 - 0.671)	0.043

Multimorbidity definition	Lookback period (year)	Unique case definition			Validated case definition[a]			Validated case definition with hypertension[b]		
		c-statistic (95% IC)[c]		Brier scaled score	c-statistic (95% IC)[c]		Brier scaled score	c-statistic (95% IC)[c]		Brier scaled score
	14	0.670	(0.668 - 0.671)	0.043	0.667	(0.666 - 0.669)	0.047	0.669	(0.668 - 0.670)	0.042
	15	0.669	(0.668 - 0.671)	0.042	0.668	(0.666 - 0.669)	0.047	0.668	(0.667 - 0.670)	0.042
	16	0.669	(0.667 - 0.670)	0.042	0.668	(0.667 - 0.670)	0.046	0.668	(0.667 - 0.669)	0.041
	17	0.668	(0.667 - 0.669)	0.041	0.668	(0.667 - 0.670)	0.046	0.668	(0.666 - 0.669)	0.041
	18	0.667	(0.666 - 0.669)	0.040	0.669	(0.667 - 0.670)	0.046	0.667	(0.666 - 0.669)	0.041
	19	0.667	(0.665 - 0.668)	0.040	0.669	(0.667 - 0.670)	0.046	0.667	(0.666 - 0.669)	0.040
	20	0.666	(0.664 - 0.667)	0.039	0.669	(0.667 - 0.670)	0.046	0.666	(0.665 - 0.668)	0.040
MM4+	1	0.591	(0.590 - 0.593)	0.021	0.592	(0.591 - 0.594)	0.022	0.602	(0.601 - 0.604)	0.028
	2	0.604	(0.603 - 0.606)	0.029	0.606	(0.605 - 0.608)	0.030	0.620	(0.618 - 0.621)	0.037
	3	0.615	(0.614 - 0.617)	0.034	0.615	(0.613 - 0.617)	0.034	0.631	(0.629 - 0.633)	0.041
	4	0.624	(0.622 - 0.625)	0.038	0.622	(0.620 - 0.623)	0.037	0.639	(0.638 - 0.641)	0.044
	5	0.631	(0.629 - 0.632)	0.041	0.627	(0.626 - 0.629)	0.039	0.645	(0.643 - 0.647)	0.045
	6	0.636	(0.634 - 0.638)	0.042	0.631	(0.629 - 0.633)	0.040	0.649	(0.647 - 0.650)	0.046
	7	0.640	(0.639 - 0.642)	0.043	0.634	(0.632 - 0.635)	0.041	0.652	(0.650 - 0.653)	0.046
	8	0.644	(0.643 - 0.646)	0.044	0.636	(0.635 - 0.638)	0.042	0.654	(0.653 - 0.656)	0.047
	9	0.648	(0.646 - 0.649)	0.045	0.639	(0.637 - 0.640)	0.042	0.656	(0.655 - 0.658)	0.047
	10	0.651	(0.649 - 0.652)	0.045	0.640	(0.639 - 0.642)	0.043	0.658	(0.656 - 0.659)	0.047
	11	0.653	(0.651 - 0.654)	0.046	0.642	(0.640 - 0.643)	0.044	0.659	(0.658 - 0.661)	0.047
	12	0.655	(0.653 - 0.656)	0.046	0.643	(0.642 - 0.645)	0.044	0.660	(0.659 - 0.662)	0.047
	13	0.656	(0.655 - 0.658)	0.046	0.645	(0.643 - 0.646)	0.044	0.661	(0.660 - 0.663)	0.047
	14	0.658	(0.656 - 0.659)	0.045	0.646	(0.644 - 0.647)	0.044	0.662	(0.660 - 0.664)	0.047
	15	0.659	(0.658 - 0.661)	0.045	0.647	(0.645 - 0.648)	0.045	0.663	(0.661 - 0.664)	0.047
	16	0.660	(0.659 - 0.662)	0.045	0.648	(0.646 - 0.649)	0.045	0.663	(0.662 - 0.665)	0.047
	17	0.661	(0.660 - 0.663)	0.045	0.649	(0.647 - 0.650)	0.045	0.664	(0.662 - 0.665)	0.047
	18	0.662	(0.660 - 0.663)	0.045	0.649	(0.648 - 0.651)	0.045	0.664	(0.662 - 0.665)	0.046
	19	0.663	(0.661 - 0.664)	0.044	0.650	(0.649 - 0.652)	0.045	0.664	(0.663 - 0.666)	0.046
	20	0.663	(0.661 - 0.664)	0.044	0.651	(0.649 - 0.652)	0.045	0.665	(0.663 - 0.666)	0.046

Abbreviations: MM2+: multimorbidity defined as ≥2 chronic conditions; MM3+: multimorbidity defined as ≥3 chronic conditions; MM4+: multimorbidity defined as ≥4 chronic conditions; na: not applicable

a All case definitions are validated and available in Table A1.3

b All case definitions are validated and available in Table A1.3. Hypertension has been added to the Core list.

c Shaded areas indicate the length of lookback period where the c-statistic ranged in the standard error interval [±0.001] of the maximal c-statistic.

d The baseline model includes only the covariates (age group, sex, material and social deprivation)

Table A2.3.6: Predictive performance (c-statistic) and model adjustment (Brier scaled score) to predict polypharmacy (≥10/year) for the Core list (L20) by criterion used to define multimorbidity, length of lookback period, and type of case definition (unique case definition for all diseases, case definition specific for some diseases with/without hypertension)

Multimorbidity definition	Lookback period (year)	Unique case definition			Validated case definition[a]			Validated case definition with hypertension[b]		
		c-statistic (95% IC)[c]		Brier scaled score	c-statistic (95% IC)[c]		Brier scaled score	c-statistic (95% IC)[c]		Brier scaled score
Baseline model[d]	na	0.621	(0.620 - 0.622)	0.042	0.621	(0.620 - 0.622)	0.042	0.621	(0.620 - 0.622)	0.042
MM2+	1	0.668	(0.667 - 0.669)	0.098	0.676	(0.675 - 0.677)	0.108	0.680	(0.679 - 0.681)	0.108
	2	0.698	(0.697 - 0.699)	0.129	0.705	(0.704 - 0.706)	0.138	0.708	(0.707 - 0.709)	0.135
	3	0.716	(0.715 - 0.717)	0.147	0.720	(0.719 - 0.721)	0.154	0.721	(0.720 - 0.722)	0.148
	4	0.726	(0.725 - 0.726)	0.156	0.729	(0.728 - 0.730)	0.163	0.727	(0.726 - 0.728)	0.154
	5	0.731	(0.730 - 0.732)	0.161	0.733	(0.733 - 0.734)	0.167	0.729	(0.728 - 0.730)	0.156
	6	0.733	(0.732 - 0.734)	0.162	0.736	(0.735 - 0.737)	0.170	0.730	(0.729 - 0.731)	0.158
	7	0.734	(0.733 - 0.735)	0.162	0.737	(0.736 - 0.738)	0.171	0.729	(0.729 - 0.730)	0.158
	8	0.734	(0.733 - 0.735)	0.161	0.738	(0.737 - 0.739)	0.172	0.729	(0.728 - 0.730)	0.158
	9	0.733	(0.732 - 0.734)	0.160	0.738	(0.737 - 0.739)	0.173	0.728	(0.727 - 0.729)	0.157
	10	0.732	(0.731 - 0.733)	0.158	0.738	(0.738 - 0.739)	0.173	0.727	(0.726 - 0.728)	0.157
	11	0.730	(0.730 - 0.731)	0.156	0.738	(0.738 - 0.739)	0.173	0.726	(0.725 - 0.727)	0.157
	12	0.729	(0.728 - 0.730)	0.154	0.738	(0.737 - 0.739)	0.172	0.725	(0.724 - 0.726)	0.156
	13	0.727	(0.727 - 0.728)	0.152	0.738	(0.737 - 0.739)	0.172	0.724	(0.723 - 0.725)	0.155
	14	0.726	(0.725 - 0.727)	0.150	0.738	(0.737 - 0.739)	0.171	0.723	(0.722 - 0.724)	0.155
	15	0.724	(0.723 - 0.725)	0.148	0.737	(0.736 - 0.738)	0.171	0.722	(0.721 - 0.723)	0.154
	16	0.722	(0.721 - 0.723)	0.146	0.737	(0.736 - 0.738)	0.170	0.721	(0.720 - 0.722)	0.153
	17	0.720	(0.719 - 0.721)	0.144	0.736	(0.736 - 0.737)	0.170	0.720	(0.719 - 0.721)	0.153
	18	0.719	(0.718 - 0.719)	0.141	0.736	(0.735 - 0.737)	0.169	0.719	(0.718 - 0.720)	0.152
	19	0.717	(0.716 - 0.718)	0.139	0.735	(0.735 - 0.736)	0.169	0.718	(0.718 - 0.719)	0.151
	20	0.715	(0.714 - 0.716)	0.138	0.735	(0.734 - 0.736)	0.168	0.718	(0.717 - 0.719)	0.151
MM3+	1	0.648	(0.647 - 0.649)	0.081	0.651	(0.650 - 0.652)	0.084	0.660	(0.659 - 0.661)	0.093
	2	0.670	(0.669 - 0.671)	0.108	0.674	(0.673 - 0.675)	0.114	0.687	(0.686 - 0.688)	0.126
	3	0.687	(0.686 - 0.688)	0.128	0.689	(0.688 - 0.690)	0.133	0.705	(0.704 - 0.706)	0.147
	4	0.700	(0.699 - 0.701)	0.142	0.701	(0.700 - 0.701)	0.146	0.718	(0.717 - 0.719)	0.161
	5	0.709	(0.708 - 0.710)	0.152	0.709	(0.708 - 0.710)	0.156	0.727	(0.726 - 0.727)	0.171
	6	0.716	(0.716 - 0.717)	0.159	0.714	(0.713 - 0.715)	0.162	0.732	(0.731 - 0.733)	0.177
	7	0.722	(0.721 - 0.723)	0.164	0.718	(0.718 - 0.719)	0.167	0.736	(0.735 - 0.737)	0.181
	8	0.726	(0.725 - 0.727)	0.167	0.722	(0.721 - 0.723)	0.170	0.739	(0.738 - 0.740)	0.185
	9	0.729	(0.728 - 0.730)	0.169	0.724	(0.723 - 0.725)	0.173	0.741	(0.740 - 0.742)	0.188
	10	0.731	(0.730 - 0.732)	0.170	0.726	(0.726 - 0.727)	0.175	0.743	(0.742 - 0.744)	0.190
	11	0.733	(0.732 - 0.734)	0.171	0.728	(0.727 - 0.729)	0.177	0.744	(0.743 - 0.745)	0.191
	12	0.734	(0.733 - 0.735)	0.172	0.730	(0.729 - 0.731)	0.178	0.745	(0.745 - 0.746)	0.192
	13	0.735	(0.735 - 0.736)	0.172	0.731	(0.730 - 0.732)	0.179	0.746	(0.745 - 0.747)	0.193
	14	0.736	(0.735 - 0.737)	0.172	0.733	(0.732 - 0.734)	0.181	0.747	(0.746 - 0.748)	0.194

Multimorbidity definition	Lookback period (year)	Unique case definition			Validated case definition[a]			Validated case definition with hypertension[b]		
		c-statistic (95% IC)[c]		Brier scaled score	c-statistic (95% IC)[c]		Brier scaled score	c-statistic (95% IC)[c]		Brier scaled score
	15	0.737	(0.736 - 0.738)	0.172	0.734	(0.733 - 0.735)	0.182	0.747	(0.746 - 0.748)	0.195
	16	0.737	(0.736 - 0.738)	0.171	0.735	(0.734 - 0.736)	0.183	0.748	(0.747 - 0.748)	0.195
	17	0.737	(0.737 - 0.738)	0.171	0.736	(0.735 - 0.737)	0.184	0.748	(0.747 - 0.749)	0.195
	18	0.737	(0.736 - 0.738)	0.170	0.736	(0.736 - 0.737)	0.184	0.748	(0.747 - 0.749)	0.195
	19	0.737	(0.736 - 0.738)	0.169	0.737	(0.736 - 0.738)	0.185	0.748	(0.747 - 0.749)	0.195
	20	0.737	(0.736 - 0.738)	0.169	0.737	(0.737 - 0.738)	0.185	0.748	(0.747 - 0.749)	0.195
MM4+	1	0.636	(0.635 - 0.637)	0.066	0.637	(0.636 - 0.638)	0.067	0.646	(0.645 - 0.647)	0.079
	2	0.649	(0.648 - 0.650)	0.084	0.650	(0.649 - 0.651)	0.086	0.665	(0.664 - 0.666)	0.105
	3	0.660	(0.659 - 0.661)	0.100	0.660	(0.659 - 0.661)	0.101	0.679	(0.678 - 0.680)	0.124
	4	0.670	(0.669 - 0.671)	0.112	0.669	(0.668 - 0.670)	0.112	0.691	(0.690 - 0.692)	0.139
	5	0.678	(0.677 - 0.679)	0.123	0.676	(0.675 - 0.677)	0.122	0.700	(0.699 - 0.701)	0.151
	6	0.685	(0.684 - 0.686)	0.131	0.680	(0.679 - 0.681)	0.128	0.706	(0.705 - 0.707)	0.159
	7	0.691	(0.690 - 0.692)	0.138	0.685	(0.684 - 0.686)	0.134	0.711	(0.710 - 0.712)	0.165
	8	0.697	(0.696 - 0.697)	0.144	0.688	(0.687 - 0.689)	0.138	0.715	(0.714 - 0.716)	0.170
	9	0.701	(0.700 - 0.702)	0.148	0.691	(0.690 - 0.692)	0.142	0.719	(0.718 - 0.720)	0.174
	10	0.705	(0.704 - 0.706)	0.152	0.694	(0.693 - 0.695)	0.145	0.722	(0.721 - 0.723)	0.177
	11	0.709	(0.708 - 0.710)	0.156	0.696	(0.695 - 0.697)	0.147	0.724	(0.723 - 0.725)	0.180
	12	0.712	(0.711 - 0.713)	0.159	0.698	(0.697 - 0.699)	0.150	0.726	(0.726 - 0.727)	0.183
	13	0.715	(0.714 - 0.716)	0.161	0.700	(0.699 - 0.701)	0.152	0.728	(0.727 - 0.729)	0.185
	14	0.717	(0.716 - 0.718)	0.163	0.702	(0.701 - 0.703)	0.155	0.730	(0.729 - 0.731)	0.187
	15	0.720	(0.719 - 0.721)	0.165	0.704	(0.703 - 0.705)	0.157	0.732	(0.731 - 0.733)	0.189
	16	0.722	(0.721 - 0.723)	0.167	0.705	(0.704 - 0.706)	0.158	0.733	(0.733 - 0.734)	0.191
	17	0.724	(0.723 - 0.725)	0.168	0.707	(0.706 - 0.708)	0.160	0.735	(0.734 - 0.736)	0.192
	18	0.725	(0.724 - 0.726)	0.169	0.708	(0.707 - 0.709)	0.162	0.736	(0.735 - 0.737)	0.194
	19	0.727	(0.726 - 0.728)	0.170	0.709	(0.708 - 0.710)	0.163	0.737	(0.736 - 0.738)	0.195
	20	0.728	(0.727 - 0.729)	0.171	0.710	(0.709 - 0.711)	0.164	0.738	(0.737 - 0.738)	0.195

Abbreviations: MM2+: multimorbidity defined as ≥2 chronic conditions; MM3+: multimorbidity defined as ≥3 chronic conditions; MM4+: multimorbidity defined as ≥4 chronic conditions; na: not applicable

a All case definitions are validated and available in Table A1.3

b All case definitions are validated and available in Table A1.3. Hypertension has been added to the Core list.

c Shaded areas indicate the length of lookback period where the c-statistic ranged in the standard error interval [±0.001] of the maximal c-statistic.

d The baseline model includes only the covariates (age group, sex, material and social deprivation)

Table A2.4.1: Predictive performance (c-statistic) and model adjustment (Brier scaled score) to predict 1-year mortality for the Charlson & Elixhauser list (L31) by criterion used to define multimorbidity, length of lookback period, and type of case definition (unique case definition for all diseases, case definition specific for some diseases)

		Unique case definition			Specific case definition[a]		
Multimorbidity definition	Lookback period (year)	c-statistic (95% IC)[b]		Brier scaled score	c-statistic (95% IC)[b]		Brier scaled score
Baseline model[c]	na	0.758	(0.756 - 0.760)	0.048	0.758	(0.756 - 0.760)	0.048
MM2+	1	0.808	(0.806 - 0.810)	0.062	0.808	(0.806 - 0.810)	0.062
	2	0.809	(0.807 - 0.810)	0.061	0.809	(0.807 - 0.810)	0.061
	3	0.807	(0.805 - 0.808)	0.061	0.807	(0.805 - 0.808)	0.061
	4	0.804	(0.802 - 0.806)	0.060	0.804	(0.802 - 0.806)	0.060
	5	0.802	(0.800 - 0.803)	0.060	0.802	(0.800 - 0.803)	0.060
	6	0.799	(0.798 - 0.801)	0.059	0.800	(0.798 - 0.801)	0.059
	7	0.797	(0.796 - 0.799)	0.059	0.798	(0.796 - 0.799)	0.059
	8	0.796	(0.794 - 0.798)	0.058	0.796	(0.794 - 0.798)	0.058
	9	0.794	(0.792 - 0.796)	0.058	0.795	(0.793 - 0.797)	0.058
	10	0.793	(0.791 - 0.795)	0.057	0.793	(0.792 - 0.795)	0.058
	11	0.792	(0.790 - 0.793)	0.057	0.792	(0.791 - 0.794)	0.057
	12	0.790	(0.789 - 0.792)	0.057	0.791	(0.789 - 0.793)	0.057
	13	0.789	(0.788 - 0.791)	0.056	0.790	(0.789 - 0.792)	0.056
	14	0.789	(0.787 - 0.790)	0.056	0.790	(0.788 - 0.791)	0.056
	15	0.788	(0.786 - 0.790)	0.056	0.789	(0.787 - 0.791)	0.056
	16	0.787	(0.785 - 0.789)	0.055	0.788	(0.786 - 0.790)	0.056
	17	0.786	(0.784 - 0.788)	0.055	0.787	(0.785 - 0.789)	0.055
	18	0.785	(0.783 - 0.787)	0.055	0.787	(0.785 - 0.789)	0.055
	19	0.785	(0.783 - 0.786)	0.055	0.786	(0.784 - 0.788)	0.055
	20	0.784	(0.782 - 0.786)	0.055	0.786	(0.784 - 0.788)	0.055
MM3+	1	0.805	(0.803 - 0.807)	0.065	0.805	(0.803 - 0.807)	0.065
	2	0.812	(0.810 - 0.814)	0.066	0.812	(0.810 - 0.814)	0.066
	3	0.814	(0.812 - 0.816)	0.067	0.814	(0.812 - 0.816)	0.067
	4	0.814	(0.812 - 0.816)	0.067	0.814	(0.812 - 0.816)	0.067
	5	0.813	(0.811 - 0.815)	0.067	0.813	(0.811 - 0.815)	0.067
	6	0.812	(0.810 - 0.814)	0.066	0.812	(0.810 - 0.814)	0.066
	7	0.811	(0.809 - 0.813)	0.066	0.811	(0.809 - 0.813)	0.066
	8	0.810	(0.808 - 0.812)	0.065	0.810	(0.808 - 0.812)	0.065
	9	0.809	(0.807 - 0.810)	0.065	0.809	(0.807 - 0.811)	0.065
	10	0.807	(0.806 - 0.809)	0.064	0.808	(0.806 - 0.809)	0.064
	11	0.806	(0.805 - 0.808)	0.064	0.807	(0.805 - 0.809)	0.064
	12	0.805	(0.804 - 0.807)	0.063	0.806	(0.804 - 0.808)	0.063
	13	0.804	(0.803 - 0.806)	0.063	0.805	(0.803 - 0.807)	0.063
	14	0.804	(0.802 - 0.805)	0.062	0.804	(0.803 - 0.806)	0.062
	15	0.803	(0.801 - 0.804)	0.062	0.804	(0.802 - 0.805)	0.062
	16	0.802	(0.800 - 0.804)	0.061	0.803	(0.801 - 0.805)	0.062
	17	0.801	(0.799 - 0.803)	0.061	0.802	(0.801 - 0.804)	0.061
	18	0.800	(0.799 - 0.802)	0.061	0.802	(0.800 - 0.804)	0.061

Multimorbidity definition	Lookback period (year)	Unique case definition: c-statistic (95% IC)[b]	Unique case definition: Brier scaled score	Specific case definition[a]: c-statistic (95% IC)[b]	Specific case definition[a]: Brier scaled score
	19	0.799 (0.798 - 0.801)	0.060	0.801 (0.799 - 0.803)	0.061
	20	0.799 (0.797 - 0.800)	0.060	0.801 (0.799 - 0.802)	0.061
MM4+	1	0.801 (0.799 - 0.803)	0.067	0.801 (0.799 - 0.803)	0.067
	2	0.809 (0.807 - 0.811)	0.069	0.809 (0.807 - 0.811)	0.069
	3	0.812 (0.811 - 0.814)	0.070	0.812 (0.811 - 0.814)	0.070
	4	0.814 (0.812 - 0.816)	0.071	0.814 (0.812 - 0.816)	0.071
	5	0.815 (0.813 - 0.817)	0.071	0.815 (0.813 - 0.817)	0.071
	6	0.815 (0.813 - 0.816)	0.070	0.815 (0.813 - 0.816)	0.070
	7	0.815 (0.813 - 0.817)	0.070	0.815 (0.813 - 0.816)	0.070
	8	0.814 (0.813 - 0.816)	0.070	0.814 (0.812 - 0.816)	0.070
	9	0.814 (0.812 - 0.815)	0.070	0.813 (0.812 - 0.815)	0.070
	10	0.813 (0.811 - 0.815)	0.069	0.813 (0.811 - 0.815)	0.069
	11	0.813 (0.811 - 0.814)	0.069	0.813 (0.811 - 0.814)	0.069
	12	0.812 (0.811 - 0.814)	0.069	0.812 (0.811 - 0.814)	0.069
	13	0.812 (0.810 - 0.814)	0.068	0.812 (0.810 - 0.814)	0.068
	14	0.811 (0.810 - 0.813)	0.068	0.811 (0.810 - 0.813)	0.068
	15	0.811 (0.809 - 0.812)	0.067	0.811 (0.809 - 0.813)	0.067
	16	0.810 (0.809 - 0.812)	0.067	0.811 (0.809 - 0.812)	0.067
	17	0.810 (0.808 - 0.812)	0.066	0.810 (0.808 - 0.812)	0.067
	18	0.809 (0.808 - 0.811)	0.066	0.810 (0.808 - 0.812)	0.066
	19	0.809 (0.807 - 0.811)	0.066	0.809 (0.808 - 0.811)	0.066
	20	0.808 (0.807 - 0.810)	0.066	0.809 (0.807 - 0.811)	0.066

Abbreviations: MM2+: multimorbidity defined as ≥2 chronic conditions; MM3+: multimorbidity defined as ≥3 chronic conditions; MM4+: multimorbidity defined as ≥4 chronic conditions; na: not applicable

a The maximum length of lookback period is 5 years for: depression; psychoses; alcohol abuse; drug abuse.

b Shaded areas indicate the length of lookback period where the c-statistic ranged in the standard error interval [±0.001] of the maximal c-statistic.

c The baseline model includes only the covariates (age group, sex, material and social deprivation)

Table A2.4.2: Predictive performance (c-statistic) and model adjustment (Brier scaled score) to predict hospitalisation (≥1/year) for the Charlson & Elixhauser list (L31) by criterion used to define multimorbidity, length of lookback period, and type of case definition (unique case definition for all diseases, case definition specific for some diseases)

		Unique case definition			Specific case definition[a]		
Multimorbidity definition	Lookback period (year)	c-statistic (95% IC)[b]		Brier scaled score	c-statistic (95% IC)[b]		Brier scaled score
Baseline model[c]	na	0.615	(0.614 - 0.617)	0.019	0.615	(0.614 - 0.617)	0.019
MM2+	1	0.667	(0.665 - 0.668)	0.047	0.667	(0.665 - 0.668)	0.047
	2	0.676	(0.675 - 0.678)	0.047	0.676	(0.675 - 0.678)	0.047
	3	0.677	(0.676 - 0.678)	0.045	0.677	(0.676 - 0.678)	0.045
	4	0.676	(0.674 - 0.677)	0.043	0.676	(0.674 - 0.677)	0.043
	5	0.674	(0.672 - 0.675)	0.041	0.674	(0.672 - 0.675)	0.041
	6	0.671	(0.670 - 0.673)	0.039	0.671	(0.670 - 0.673)	0.039
	7	0.669	(0.668 - 0.671)	0.038	0.669	(0.668 - 0.671)	0.038
	8	0.667	(0.666 - 0.669)	0.037	0.668	(0.666 - 0.669)	0.037
	9	0.666	(0.664 - 0.667)	0.036	0.666	(0.665 - 0.668)	0.037
	10	0.664	(0.663 - 0.665)	0.035	0.665	(0.663 - 0.666)	0.036
	11	0.663	(0.661 - 0.664)	0.035	0.663	(0.662 - 0.665)	0.035
	12	0.661	(0.660 - 0.663)	0.034	0.662	(0.661 - 0.663)	0.035
	13	0.660	(0.659 - 0.661)	0.034	0.661	(0.660 - 0.662)	0.034
	14	0.659	(0.658 - 0.660)	0.033	0.660	(0.659 - 0.662)	0.034
	15	0.658	(0.656 - 0.659)	0.033	0.659	(0.658 - 0.661)	0.033
	16	0.657	(0.655 - 0.658)	0.032	0.658	(0.657 - 0.660)	0.033
	17	0.656	(0.655 - 0.657)	0.032	0.658	(0.656 - 0.659)	0.033
	18	0.655	(0.654 - 0.657)	0.032	0.657	(0.655 - 0.658)	0.032
	19	0.654	(0.653 - 0.656)	0.031	0.656	(0.655 - 0.658)	0.032
	20	0.654	(0.652 - 0.655)	0.031	0.656	(0.654 - 0.657)	0.032
MM3+	1	0.658	(0.656 - 0.660)	0.047	0.658	(0.656 - 0.660)	0.047
	2	0.672	(0.670 - 0.673)	0.051	0.672	(0.670 - 0.673)	0.051
	3	0.677	(0.676 - 0.679)	0.051	0.677	(0.676 - 0.679)	0.051
	4	0.679	(0.678 - 0.681)	0.049	0.679	(0.678 - 0.681)	0.049
	5	0.680	(0.679 - 0.682)	0.048	0.680	(0.679 - 0.682)	0.048
	6	0.680	(0.679 - 0.682)	0.048	0.680	(0.679 - 0.682)	0.048
	7	0.680	(0.679 - 0.682)	0.047	0.680	(0.679 - 0.682)	0.047
	8	0.680	(0.678 - 0.681)	0.046	0.680	(0.678 - 0.681)	0.046
	9	0.679	(0.677 - 0.680)	0.045	0.679	(0.677 - 0.680)	0.045
	10	0.678	(0.676 - 0.679)	0.044	0.678	(0.677 - 0.680)	0.044
	11	0.677	(0.676 - 0.679)	0.043	0.677	(0.676 - 0.679)	0.044
	12	0.677	(0.675 - 0.678)	0.043	0.677	(0.675 - 0.678)	0.043
	13	0.676	(0.674 - 0.677)	0.042	0.676	(0.675 - 0.678)	0.043
	14	0.675	(0.673 - 0.676)	0.042	0.675	(0.674 - 0.677)	0.042
	15	0.674	(0.673 - 0.676)	0.041	0.675	(0.673 - 0.676)	0.042
	16	0.674	(0.672 - 0.675)	0.041	0.674	(0.673 - 0.676)	0.041
	17	0.673	(0.671 - 0.674)	0.040	0.674	(0.672 - 0.675)	0.041
	18	0.672	(0.671 - 0.674)	0.040	0.673	(0.672 - 0.675)	0.040

Multimorbidity definition	Lookback period (year)	Unique case definition: c-statistic (95% IC)[b]		Unique case definition: Brier scaled score	Specific case definition[a]: c-statistic (95% IC)[b]		Specific case definition[a]: Brier scaled score
	19	0.671	(0.670 - 0.673)	0.039	0.673	(0.671 - 0.674)	0.040
	20	0.671	(0.669 - 0.672)	0.039	0.672	(0.671 - 0.674)	0.040
MM4+	1	0.650	(0.649 - 0.652)	0.045	0.650	(0.649 - 0.652)	0.045
	2	0.663	(0.661 - 0.664)	0.050	0.663	(0.661 - 0.664)	0.050
	3	0.670	(0.668 - 0.671)	0.051	0.670	(0.668 - 0.671)	0.051
	4	0.674	(0.672 - 0.675)	0.052	0.674	(0.672 - 0.675)	0.052
	5	0.676	(0.674 - 0.678)	0.051	0.676	(0.674 - 0.678)	0.051
	6	0.678	(0.676 - 0.679)	0.051	0.678	(0.676 - 0.679)	0.051
	7	0.679	(0.678 - 0.681)	0.051	0.679	(0.677 - 0.680)	0.051
	8	0.680	(0.678 - 0.681)	0.050	0.679	(0.678 - 0.681)	0.050
	9	0.680	(0.679 - 0.682)	0.050	0.680	(0.678 - 0.681)	0.050
	10	0.680	(0.679 - 0.682)	0.049	0.680	(0.679 - 0.682)	0.049
	11	0.681	(0.679 - 0.682)	0.049	0.680	(0.679 - 0.682)	0.049
	12	0.680	(0.679 - 0.682)	0.048	0.680	(0.679 - 0.682)	0.049
	13	0.681	(0.679 - 0.682)	0.048	0.680	(0.679 - 0.682)	0.048
	14	0.680	(0.679 - 0.682)	0.047	0.680	(0.679 - 0.682)	0.048
	15	0.680	(0.679 - 0.682)	0.047	0.680	(0.679 - 0.681)	0.048
	16	0.680	(0.679 - 0.681)	0.047	0.680	(0.678 - 0.681)	0.047
	17	0.680	(0.678 - 0.681)	0.046	0.680	(0.678 - 0.681)	0.047
	18	0.679	(0.678 - 0.681)	0.046	0.679	(0.678 - 0.681)	0.047
	19	0.679	(0.678 - 0.681)	0.046	0.679	(0.678 - 0.681)	0.046
	20	0.679	(0.677 - 0.680)	0.045	0.679	(0.678 - 0.681)	0.046

Abbreviations: MM2+: multimorbidity defined as ≥2 chronic conditions; MM3+: multimorbidity defined as ≥3 chronic conditions; MM4+: multimorbidity defined as ≥4 chronic conditions; na: not applicable

a The maximum length of lookback period is 5 years for: depression; psychoses; alcohol abuse; drug abuse.
b Shaded areas indicate the length of lookback period where the c-statistic ranged in the standard error interval [±0.001] of the maximal c-statistic.

c The baseline model includes only the covariates (age group, sex, material and social deprivation)

Table A2.4.3: Predictive performance (c-statistic) and model adjustment (Brier scaled score) to predict frequent visits to emergency department (≥3/year) for the Charlson & Elixhauser list (L31) by criterion used to define multimorbidity, length of lookback period, and type of case definition (unique case definition for all diseases, case definition specific for some diseases)

Multimorbidity definition	Lookback period (year)	Unique case definition			Specific case definition[a]		
		c-statistic (95% IC)[b]		Brier scaled score	c-statistic (95% IC)[b]		Brier scaled score
Baseline model[c]	na	0.634	(0.632 - 0.636)	0.011	0.634	(0.632 - 0.636)	0.011
MM2+	1	0.698	(0.696 - 0.700)	0.032	0.698	(0.696 - 0.700)	0.032
	2	0.709	(0.707 - 0.711)	0.031	0.709	(0.707 - 0.711)	0.031
	3	0.711	(0.709 - 0.713)	0.029	0.711	(0.709 - 0.713)	0.029
	4	0.709	(0.707 - 0.712)	0.027	0.709	(0.707 - 0.712)	0.027
	5	0.707	(0.704 - 0.709)	0.026	0.707	(0.704 - 0.709)	0.026
	6	0.704	(0.702 - 0.706)	0.025	0.704	(0.702 - 0.706)	0.025
	7	0.702	(0.700 - 0.704)	0.024	0.702	(0.700 - 0.704)	0.024
	8	0.699	(0.697 - 0.701)	0.023	0.699	(0.697 - 0.701)	0.023
	9	0.696	(0.694 - 0.698)	0.022	0.696	(0.694 - 0.698)	0.022
	10	0.694	(0.692 - 0.696)	0.021	0.694	(0.692 - 0.696)	0.022
	11	0.692	(0.690 - 0.694)	0.021	0.692	(0.690 - 0.695)	0.021
	12	0.690	(0.688 - 0.692)	0.020	0.691	(0.689 - 0.693)	0.021
	13	0.688	(0.686 - 0.690)	0.020	0.689	(0.687 - 0.691)	0.020
	14	0.687	(0.685 - 0.689)	0.020	0.688	(0.686 - 0.690)	0.020
	15	0.685	(0.683 - 0.687)	0.019	0.687	(0.685 - 0.689)	0.020
	16	0.684	(0.682 - 0.686)	0.019	0.685	(0.683 - 0.687)	0.019
	17	0.683	(0.681 - 0.685)	0.019	0.685	(0.683 - 0.687)	0.019
	18	0.682	(0.680 - 0.684)	0.019	0.684	(0.682 - 0.686)	0.019
	19	0.681	(0.679 - 0.683)	0.018	0.683	(0.681 - 0.685)	0.019
	20	0.680	(0.678 - 0.682)	0.018	0.682	(0.680 - 0.684)	0.019
MM3+	1	0.689	(0.687 - 0.691)	0.033	0.689	(0.687 - 0.691)	0.033
	2	0.706	(0.703 - 0.708)	0.035	0.706	(0.703 - 0.708)	0.035
	3	0.713	(0.711 - 0.715)	0.034	0.713	(0.711 - 0.715)	0.034
	4	0.715	(0.713 - 0.717)	0.033	0.715	(0.713 - 0.717)	0.033
	5	0.716	(0.714 - 0.718)	0.032	0.716	(0.714 - 0.718)	0.032
	6	0.716	(0.714 - 0.718)	0.031	0.716	(0.714 - 0.718)	0.031
	7	0.716	(0.713 - 0.718)	0.030	0.715	(0.713 - 0.718)	0.030
	8	0.715	(0.713 - 0.717)	0.029	0.715	(0.712 - 0.717)	0.029
	9	0.714	(0.712 - 0.716)	0.029	0.714	(0.712 - 0.716)	0.029
	10	0.713	(0.710 - 0.715)	0.028	0.713	(0.711 - 0.715)	0.028
	11	0.711	(0.709 - 0.713)	0.027	0.711	(0.709 - 0.714)	0.028
	12	0.710	(0.708 - 0.712)	0.027	0.711	(0.709 - 0.713)	0.027
	13	0.709	(0.707 - 0.711)	0.026	0.710	(0.708 - 0.712)	0.027
	14	0.708	(0.706 - 0.710)	0.026	0.709	(0.707 - 0.711)	0.026
	15	0.707	(0.705 - 0.709)	0.025	0.708	(0.706 - 0.710)	0.026
	16	0.706	(0.704 - 0.708)	0.025	0.707	(0.705 - 0.709)	0.026
	17	0.705	(0.703 - 0.707)	0.025	0.706	(0.704 - 0.708)	0.025

Multimorbidity definition	Lookback period (year)	Unique case definition: c-statistic (95% IC)[b]		Unique case definition: Brier scaled score	Specific case definition[a]: c-statistic (95% IC)[b]		Specific case definition[a]: Brier scaled score
	18	0.704	(0.702 - 0.706)	0.024	0.705	(0.703 - 0.707)	0.025
	19	0.703	(0.701 - 0.706)	0.024	0.705	(0.703 - 0.707)	0.025
	20	0.703	(0.700 - 0.705)	0.024	0.704	(0.702 - 0.706)	0.024
MM4+	1	0.680	(0.678 - 0.683)	0.032	0.680	(0.678 - 0.683)	0.032
	2	0.696	(0.694 - 0.699)	0.035	0.696	(0.694 - 0.699)	0.035
	3	0.705	(0.703 - 0.708)	0.036	0.705	(0.703 - 0.708)	0.036
	4	0.710	(0.708 - 0.713)	0.036	0.710	(0.708 - 0.713)	0.036
	5	0.714	(0.711 - 0.716)	0.035	0.714	(0.711 - 0.716)	0.035
	6	0.715	(0.713 - 0.718)	0.035	0.715	(0.713 - 0.717)	0.035
	7	0.717	(0.714 - 0.719)	0.034	0.716	(0.714 - 0.719)	0.034
	8	0.718	(0.715 - 0.720)	0.034	0.717	(0.715 - 0.719)	0.034
	9	0.718	(0.716 - 0.720)	0.033	0.717	(0.715 - 0.720)	0.033
	10	0.718	(0.716 - 0.720)	0.033	0.718	(0.715 - 0.720)	0.033
	11	0.718	(0.716 - 0.720)	0.032	0.717	(0.715 - 0.719)	0.033
	12	0.717	(0.715 - 0.720)	0.032	0.717	(0.715 - 0.719)	0.032
	13	0.717	(0.715 - 0.719)	0.032	0.717	(0.715 - 0.719)	0.032
	14	0.717	(0.715 - 0.719)	0.031	0.717	(0.715 - 0.719)	0.031
	15	0.717	(0.715 - 0.719)	0.031	0.716	(0.714 - 0.719)	0.031
	16	0.716	(0.714 - 0.718)	0.030	0.716	(0.714 - 0.718)	0.031
	17	0.716	(0.714 - 0.718)	0.030	0.716	(0.714 - 0.718)	0.031
	18	0.715	(0.713 - 0.718)	0.030	0.715	(0.713 - 0.717)	0.030
	19	0.715	(0.713 - 0.718)	0.030	0.715	(0.713 - 0.717)	0.030
	20	0.715	(0.713 - 0.717)	0.029	0.715	(0.713 - 0.717)	0.030

Abbreviations: MM2+: multimorbidity defined as ≥2 chronic conditions; MM3+: multimorbidity defined as ≥3 chronic conditions; MM4+: multimorbidity defined as ≥4 chronic conditions; na: not applicable

a The maximum length of lookback period is 5 years for: depression; psychoses; alcohol abuse; drug abuse.

b Shaded areas indicate the length of lookback period where the c-statistic ranged in the standard error interval [±0.001] of the maximal c-statistic.

c The baseline model includes only the covariates (age group, sex, material and social deprivation)

Table A2.4.4: Predictive performance (c-statistic) and model adjustment (Brier scaled score) to predict frequent visits to general practitioner (≥7/year) for the Charlson & Elixhauser list (L31) by criterion used to define multimorbidity, length of lookback period, and type of case definition (unique case definition for all diseases, case definition specific for some diseases)

Multimorbidity definition	Lookback period (year)	Unique case definition c-statistic (95% IC)[b]		Unique case definition Brier scaled score	Specific case definition[a] c-statistic (95% IC)[b]		Specific case definition[a] Brier scaled score
Baseline model[c]	na	0.629	(0.627 - 0.631)	0.020	0.629	(0.627 - 0.631)	0.020
MM2+	1	0.658	(0.657 - 0.660)	0.033	0.658	(0.657 - 0.660)	0.033
	2	0.669	(0.667 - 0.670)	0.036	0.669	(0.667 - 0.670)	0.036
	3	0.675	(0.673 - 0.677)	0.037	0.675	(0.673 - 0.677)	0.037
	4	0.677	(0.676 - 0.679)	0.037	0.677	(0.676 - 0.679)	0.037
	5	0.678	(0.677 - 0.680)	0.037	0.678	(0.677 - 0.680)	0.037
	6	0.678	(0.677 - 0.680)	0.036	0.678	(0.676 - 0.680)	0.036
	7	0.678	(0.676 - 0.680)	0.035	0.677	(0.676 - 0.679)	0.035
	8	0.677	(0.675 - 0.679)	0.035	0.676	(0.675 - 0.678)	0.035
	9	0.677	(0.675 - 0.678)	0.034	0.676	(0.674 - 0.678)	0.034
	10	0.676	(0.675 - 0.678)	0.034	0.675	(0.674 - 0.677)	0.034
	11	0.676	(0.674 - 0.677)	0.034	0.675	(0.673 - 0.677)	0.034
	12	0.675	(0.674 - 0.677)	0.033	0.674	(0.673 - 0.676)	0.033
	13	0.675	(0.673 - 0.676)	0.033	0.674	(0.672 - 0.676)	0.033
	14	0.674	(0.672 - 0.675)	0.033	0.673	(0.672 - 0.675)	0.033
	15	0.673	(0.672 - 0.675)	0.033	0.673	(0.671 - 0.674)	0.032
	16	0.673	(0.671 - 0.674)	0.032	0.672	(0.671 - 0.674)	0.032
	17	0.672	(0.670 - 0.674)	0.032	0.672	(0.670 - 0.674)	0.032
	18	0.672	(0.670 - 0.673)	0.032	0.672	(0.670 - 0.673)	0.032
	19	0.671	(0.670 - 0.673)	0.032	0.671	(0.670 - 0.673)	0.032
	20	0.671	(0.669 - 0.672)	0.032	0.671	(0.669 - 0.673)	0.032
MM3+	1	0.651	(0.649 - 0.653)	0.031	0.651	(0.649 - 0.653)	0.031
	2	0.661	(0.659 - 0.662)	0.034	0.661	(0.659 - 0.662)	0.034
	3	0.668	(0.666 - 0.669)	0.036	0.668	(0.666 - 0.669)	0.036
	4	0.671	(0.670 - 0.673)	0.037	0.671	(0.670 - 0.673)	0.037
	5	0.674	(0.672 - 0.676)	0.037	0.674	(0.672 - 0.676)	0.037
	6	0.676	(0.674 - 0.678)	0.038	0.676	(0.674 - 0.677)	0.037
	7	0.677	(0.675 - 0.679)	0.037	0.676	(0.675 - 0.678)	0.037
	8	0.678	(0.676 - 0.679)	0.037	0.677	(0.675 - 0.679)	0.037
	9	0.679	(0.677 - 0.680)	0.037	0.678	(0.676 - 0.679)	0.037
	10	0.679	(0.677 - 0.681)	0.037	0.678	(0.676 - 0.680)	0.037
	11	0.680	(0.678 - 0.681)	0.037	0.678	(0.677 - 0.680)	0.037
	12	0.680	(0.678 - 0.681)	0.037	0.679	(0.677 - 0.680)	0.037
	13	0.680	(0.679 - 0.682)	0.037	0.679	(0.677 - 0.680)	0.037
	14	0.680	(0.679 - 0.682)	0.037	0.679	(0.677 - 0.681)	0.036
	15	0.681	(0.679 - 0.682)	0.037	0.679	(0.677 - 0.680)	0.036
	16	0.681	(0.679 - 0.682)	0.037	0.679	(0.677 - 0.681)	0.036
	17	0.681	(0.679 - 0.682)	0.036	0.679	(0.678 - 0.681)	0.036

Multimorbidity definition	Lookback period (year)	Unique case definition c-statistic (95% IC)[b]		Unique case definition Brier scaled score	Specific case definition[a] c-statistic (95% IC)[b]		Specific case definition[a] Brier scaled score
	18	0.681	(0.680 - 0.683)	0.036	0.679	(0.678 - 0.681)	0.036
	19	0.681	(0.680 - 0.683)	0.036	0.679	(0.678 - 0.681)	0.036
	20	0.681	(0.680 - 0.683)	0.036	0.679	(0.678 - 0.681)	0.036
MM4+	1	0.647	(0.645 - 0.648)	0.029	0.647	(0.645 - 0.648)	0.029
	2	0.654	(0.652 - 0.656)	0.032	0.654	(0.652 - 0.656)	0.032
	3	0.660	(0.658 - 0.661)	0.034	0.660	(0.658 - 0.661)	0.034
	4	0.664	(0.662 - 0.665)	0.035	0.664	(0.662 - 0.665)	0.035
	5	0.667	(0.665 - 0.668)	0.036	0.667	(0.665 - 0.668)	0.036
	6	0.669	(0.667 - 0.670)	0.037	0.668	(0.667 - 0.670)	0.036
	7	0.670	(0.669 - 0.672)	0.037	0.670	(0.668 - 0.671)	0.037
	8	0.671	(0.670 - 0.673)	0.037	0.671	(0.669 - 0.672)	0.037
	9	0.673	(0.671 - 0.674)	0.037	0.672	(0.670 - 0.673)	0.037
	10	0.674	(0.672 - 0.676)	0.037	0.673	(0.671 - 0.674)	0.037
	11	0.675	(0.673 - 0.677)	0.038	0.674	(0.672 - 0.675)	0.037
	12	0.676	(0.674 - 0.678)	0.038	0.674	(0.673 - 0.676)	0.037
	13	0.677	(0.675 - 0.678)	0.038	0.675	(0.673 - 0.677)	0.037
	14	0.677	(0.676 - 0.679)	0.038	0.675	(0.674 - 0.677)	0.037
	15	0.678	(0.676 - 0.679)	0.038	0.676	(0.674 - 0.677)	0.037
	16	0.678	(0.677 - 0.680)	0.038	0.676	(0.674 - 0.678)	0.037
	17	0.679	(0.677 - 0.681)	0.038	0.676	(0.675 - 0.678)	0.037
	18	0.679	(0.678 - 0.681)	0.038	0.677	(0.675 - 0.678)	0.037
	19	0.680	(0.678 - 0.681)	0.038	0.677	(0.675 - 0.679)	0.037
	20	0.680	(0.678 - 0.682)	0.038	0.677	(0.676 - 0.679)	0.037

Abbreviations: MM2+: multimorbidity defined as ≥2 chronic conditions; MM3+: multimorbidity defined as ≥3 chronic conditions; MM4+: multimorbidity defined as ≥4 chronic conditions; na: not applicable

a The maximum length of lookback period is 5 years for: depression; psychoses; alcohol abuse; drug abuse.

b Shaded areas indicate the length of lookback period where the c-statistic ranged in the standard error interval [±0.001] of the maximal c-statistic.

c The baseline model includes only the covariates (age group, sex, material and social deprivation)

Table A2.4.5: Predictive performance (c-statistic) and model adjustment (Brier scaled score) to predict frequent visits to specialist physician (≥10/year) for the Charlson & Elixhauser list (L31) by criterion used to define multimorbidity, length of lookback period, and type of case definition (unique case definition for all diseases, case definition specific for some diseases)

		Unique case definition		Specific case definition[a]	
Multimorbidity definition	Lookback period (year)	c-statistic (95% IC)[b]	Brier scaled score	c-statistic (95% IC)[b]	Brier scaled score
Baseline model[c]	na	0.572 (0.570 - 0.573)	0.007	0.572 (0.570 - 0.573)	0.007
MM2+	1	0.650 (0.648 - 0.652)	0.055	0.650 (0.648 - 0.652)	0.055
	2	0.674 (0.673 - 0.676)	0.058	0.674 (0.673 - 0.676)	0.058
	3	0.681 (0.680 - 0.682)	0.056	0.681 (0.680 - 0.682)	0.056
	4	0.682 (0.681 - 0.684)	0.052	0.682 (0.681 - 0.684)	0.052
	5	0.680 (0.679 - 0.682)	0.049	0.680 (0.679 - 0.682)	0.049
	6	0.677 (0.675 - 0.678)	0.046	0.677 (0.675 - 0.678)	0.046
	7	0.673 (0.672 - 0.675)	0.043	0.674 (0.672 - 0.675)	0.043
	8	0.670 (0.668 - 0.671)	0.041	0.670 (0.669 - 0.672)	0.041
	9	0.667 (0.665 - 0.668)	0.039	0.668 (0.666 - 0.669)	0.040
	10	0.664 (0.662 - 0.665)	0.037	0.665 (0.664 - 0.666)	0.038
	11	0.661 (0.660 - 0.662)	0.036	0.662 (0.661 - 0.664)	0.037
	12	0.658 (0.657 - 0.660)	0.035	0.660 (0.659 - 0.662)	0.036
	13	0.656 (0.655 - 0.657)	0.034	0.658 (0.657 - 0.660)	0.035
	14	0.654 (0.653 - 0.655)	0.033	0.657 (0.655 - 0.658)	0.034
	15	0.652 (0.651 - 0.654)	0.032	0.655 (0.654 - 0.656)	0.033
	16	0.650 (0.649 - 0.651)	0.031	0.653 (0.652 - 0.655)	0.033
	17	0.649 (0.647 - 0.650)	0.031	0.652 (0.651 - 0.653)	0.032
	18	0.647 (0.646 - 0.649)	0.030	0.651 (0.650 - 0.652)	0.032
	19	0.646 (0.645 - 0.647)	0.030	0.650 (0.649 - 0.651)	0.031
	20	0.645 (0.643 - 0.646)	0.029	0.649 (0.647 - 0.650)	0.031
MM3+	1	0.623 (0.622 - 0.625)	0.039	0.623 (0.622 - 0.625)	0.039
	2	0.647 (0.645 - 0.648)	0.050	0.647 (0.645 - 0.648)	0.050
	3	0.660 (0.659 - 0.662)	0.054	0.660 (0.659 - 0.662)	0.054
	4	0.668 (0.666 - 0.669)	0.055	0.668 (0.666 - 0.669)	0.055
	5	0.672 (0.671 - 0.674)	0.054	0.672 (0.671 - 0.674)	0.054
	6	0.675 (0.673 - 0.676)	0.053	0.674 (0.673 - 0.676)	0.053
	7	0.676 (0.675 - 0.678)	0.052	0.676 (0.674 - 0.677)	0.052
	8	0.677 (0.675 - 0.678)	0.051	0.677 (0.675 - 0.678)	0.051
	9	0.677 (0.676 - 0.679)	0.050	0.677 (0.675 - 0.678)	0.050
	10	0.677 (0.676 - 0.679)	0.049	0.677 (0.676 - 0.678)	0.050
	11	0.677 (0.675 - 0.678)	0.048	0.677 (0.675 - 0.678)	0.049
	12	0.676 (0.675 - 0.678)	0.047	0.676 (0.675 - 0.678)	0.048
	13	0.676 (0.674 - 0.677)	0.046	0.676 (0.674 - 0.677)	0.047
	14	0.675 (0.673 - 0.676)	0.045	0.675 (0.674 - 0.677)	0.046
	15	0.674 (0.672 - 0.675)	0.044	0.674 (0.673 - 0.676)	0.046
	16	0.673 (0.672 - 0.674)	0.044	0.674 (0.673 - 0.675)	0.045
	17	0.673 (0.671 - 0.674)	0.043	0.674 (0.672 - 0.675)	0.045

Multimorbidity definition	Lookback period (year)	Unique case definition			Specific case definition[a]		
		c-statistic (95% IC)[b]		Brier scaled score	c-statistic (95% IC)[b]		Brier scaled score
	18	0.672	(0.670 - 0.673)	0.043	0.673	(0.672 - 0.675)	0.044
	19	0.671	(0.670 - 0.672)	0.042	0.673	(0.672 - 0.674)	0.044
	20	0.670	(0.669 - 0.672)	0.041	0.672	(0.671 - 0.674)	0.043
MM4+	1	0.610	(0.609 - 0.612)	0.032	0.610	(0.609 - 0.612)	0.032
	2	0.628	(0.626 - 0.630)	0.041	0.628	(0.626 - 0.630)	0.041
	3	0.639	(0.638 - 0.641)	0.046	0.639	(0.638 - 0.641)	0.046
	4	0.647	(0.646 - 0.649)	0.049	0.647	(0.646 - 0.649)	0.049
	5	0.653	(0.652 - 0.655)	0.050	0.653	(0.652 - 0.655)	0.050
	6	0.657	(0.656 - 0.659)	0.051	0.657	(0.655 - 0.659)	0.051
	7	0.660	(0.659 - 0.662)	0.051	0.660	(0.658 - 0.661)	0.051
	8	0.663	(0.661 - 0.664)	0.051	0.662	(0.661 - 0.664)	0.051
	9	0.665	(0.663 - 0.666)	0.051	0.664	(0.662 - 0.665)	0.051
	10	0.666	(0.665 - 0.668)	0.050	0.665	(0.664 - 0.667)	0.051
	11	0.667	(0.666 - 0.669)	0.050	0.666	(0.665 - 0.668)	0.051
	12	0.668	(0.667 - 0.670)	0.050	0.667	(0.666 - 0.669)	0.050
	13	0.669	(0.668 - 0.671)	0.050	0.668	(0.667 - 0.670)	0.050
	14	0.670	(0.668 - 0.671)	0.049	0.669	(0.667 - 0.670)	0.050
	15	0.670	(0.669 - 0.672)	0.049	0.669	(0.668 - 0.671)	0.050
	16	0.671	(0.669 - 0.672)	0.049	0.670	(0.668 - 0.671)	0.050
	17	0.671	(0.670 - 0.673)	0.049	0.670	(0.669 - 0.672)	0.049
	18	0.672	(0.670 - 0.673)	0.048	0.671	(0.669 - 0.672)	0.049
	19	0.672	(0.671 - 0.674)	0.048	0.671	(0.670 - 0.672)	0.049
	20	0.672	(0.671 - 0.674)	0.048	0.671	(0.670 - 0.673)	0.049

Abbreviations: MM2+: multimorbidity defined as ≥2 chronic conditions; MM3+: multimorbidity defined as ≥3 chronic conditions; MM4+: multimorbidity defined as ≥4 chronic conditions; na: not applicable

a The maximum length of lookback period is 5 years for: depression; psychoses; alcohol abuse; drug abuse.

b Shaded areas indicate the length of lookback period where the c-statistic ranged in the standard error interval [±0.001] of the maximal c-statistic.

c The baseline model includes only the covariates (age group, sex, material and social deprivation)

Table A2.4.6: Predictive performance (c-statistic) and model adjustment (Brier scaled score) to predict polypharmacy (≥10/year) for the Charlson & Elixhauser list (L31) by criterion used to define multimorbidity, length of lookback period, and type of case definition (unique case definition for all diseases, case definition specific for some diseases)

		Unique case definition			Specific case definition[a]		
Multimorbidity definition	Lookback period (year)	c-statistic (95% IC)[b]		Brier scaled score	c-statistic (95% IC)[b]		Brier scaled score
Baseline model[c]	na	0.621	(0.620 - 0.622)	0.042	0.621	(0.620 - 0.622)	0.042
MM2+	1	0.671	(0.670 - 0.672)	0.098	0.671	(0.670 - 0.672)	0.098
	2	0.699	(0.698 - 0.700)	0.125	0.699	(0.698 - 0.700)	0.125
	3	0.715	(0.714 - 0.716)	0.142	0.715	(0.714 - 0.716)	0.142
	4	0.724	(0.723 - 0.725)	0.150	0.724	(0.723 - 0.725)	0.150
	5	0.729	(0.728 - 0.730)	0.155	0.729	(0.728 - 0.730)	0.155
	6	0.731	(0.730 - 0.732)	0.158	0.731	(0.730 - 0.732)	0.158
	7	0.732	(0.731 - 0.733)	0.159	0.732	(0.731 - 0.733)	0.159
	8	0.732	(0.731 - 0.733)	0.159	0.732	(0.731 - 0.733)	0.159
	9	0.732	(0.731 - 0.733)	0.159	0.732	(0.731 - 0.733)	0.160
	10	0.732	(0.731 - 0.733)	0.159	0.732	(0.731 - 0.733)	0.160
	11	0.731	(0.730 - 0.732)	0.159	0.732	(0.731 - 0.732)	0.160
	12	0.730	(0.730 - 0.731)	0.159	0.731	(0.730 - 0.732)	0.160
	13	0.729	(0.729 - 0.730)	0.158	0.730	(0.729 - 0.731)	0.159
	14	0.728	(0.727 - 0.729)	0.157	0.730	(0.729 - 0.730)	0.159
	15	0.727	(0.726 - 0.728)	0.156	0.729	(0.728 - 0.730)	0.158
	16	0.726	(0.725 - 0.727)	0.155	0.728	(0.727 - 0.729)	0.157
	17	0.725	(0.724 - 0.726)	0.154	0.727	(0.726 - 0.728)	0.157
	18	0.724	(0.723 - 0.725)	0.153	0.726	(0.726 - 0.727)	0.156
	19	0.723	(0.722 - 0.724)	0.152	0.726	(0.725 - 0.727)	0.155
	20	0.722	(0.721 - 0.722)	0.150	0.725	(0.724 - 0.726)	0.155
MM3+	1	0.659	(0.658 - 0.660)	0.089	0.659	(0.658 - 0.660)	0.089
	2	0.683	(0.682 - 0.684)	0.116	0.683	(0.682 - 0.684)	0.116
	3	0.699	(0.698 - 0.700)	0.135	0.699	(0.698 - 0.700)	0.135
	4	0.711	(0.710 - 0.712)	0.149	0.711	(0.710 - 0.712)	0.149
	5	0.720	(0.719 - 0.721)	0.158	0.720	(0.719 - 0.721)	0.158
	6	0.726	(0.725 - 0.727)	0.165	0.726	(0.725 - 0.727)	0.164
	7	0.731	(0.730 - 0.732)	0.170	0.730	(0.729 - 0.731)	0.169
	8	0.734	(0.734 - 0.735)	0.174	0.734	(0.733 - 0.734)	0.173
	9	0.737	(0.737 - 0.738)	0.177	0.736	(0.735 - 0.737)	0.176
	10	0.740	(0.739 - 0.741)	0.179	0.738	(0.738 - 0.739)	0.178
	11	0.742	(0.741 - 0.742)	0.181	0.740	(0.739 - 0.741)	0.180
	12	0.743	(0.742 - 0.744)	0.182	0.741	(0.740 - 0.742)	0.181
	13	0.744	(0.743 - 0.745)	0.183	0.743	(0.742 - 0.743)	0.182
	14	0.745	(0.744 - 0.746)	0.184	0.743	(0.743 - 0.744)	0.183
	15	0.746	(0.745 - 0.747)	0.186	0.744	(0.743 - 0.745)	0.184
	16	0.747	(0.746 - 0.748)	0.186	0.745	(0.744 - 0.746)	0.185
	17	0.747	(0.746 - 0.748)	0.187	0.746	(0.745 - 0.746)	0.186
	18	0.748	(0.747 - 0.748)	0.187	0.746	(0.745 - 0.747)	0.186

Multimorbidity definition	Lookback period (year)	Unique case definition: c-statistic (95% IC)[b]		Unique case definition: Brier scaled score	Specific case definition[a]: c-statistic (95% IC)[b]		Specific case definition[a]: Brier scaled score
	19	0.748	(0.747 - 0.748)	0.187	0.746	(0.745 - 0.747)	0.186
	20	0.747	(0.747 - 0.748)	0.186	0.746	(0.745 - 0.747)	0.186
MM4+	1	0.651	(0.650 - 0.652)	0.082	0.651	(0.650 - 0.652)	0.082
	2	0.669	(0.668 - 0.670)	0.106	0.669	(0.668 - 0.670)	0.106
	3	0.683	(0.682 - 0.684)	0.123	0.683	(0.682 - 0.684)	0.123
	4	0.694	(0.693 - 0.695)	0.136	0.694	(0.693 - 0.695)	0.136
	5	0.702	(0.701 - 0.703)	0.146	0.702	(0.701 - 0.703)	0.146
	6	0.709	(0.708 - 0.710)	0.154	0.709	(0.708 - 0.709)	0.154
	7	0.714	(0.713 - 0.715)	0.160	0.714	(0.713 - 0.714)	0.160
	8	0.719	(0.718 - 0.720)	0.165	0.718	(0.717 - 0.718)	0.164
	9	0.723	(0.722 - 0.724)	0.169	0.721	(0.720 - 0.722)	0.168
	10	0.726	(0.725 - 0.727)	0.173	0.724	(0.723 - 0.725)	0.171
	11	0.729	(0.728 - 0.730)	0.177	0.727	(0.726 - 0.728)	0.174
	12	0.732	(0.731 - 0.732)	0.179	0.729	(0.728 - 0.730)	0.177
	13	0.734	(0.733 - 0.735)	0.182	0.731	(0.730 - 0.732)	0.179
	14	0.736	(0.735 - 0.737)	0.184	0.733	(0.732 - 0.734)	0.181
	15	0.738	(0.737 - 0.739)	0.186	0.734	(0.734 - 0.735)	0.183
	16	0.740	(0.739 - 0.741)	0.188	0.736	(0.735 - 0.737)	0.184
	17	0.741	(0.740 - 0.742)	0.189	0.737	(0.736 - 0.738)	0.186
	18	0.742	(0.741 - 0.743)	0.191	0.738	(0.737 - 0.739)	0.187
	19	0.744	(0.743 - 0.745)	0.192	0.739	(0.738 - 0.740)	0.188
	20	0.745	(0.744 - 0.746)	0.193	0.740	(0.739 - 0.741)	0.189

Abbreviations: MM2+: multimorbidity defined as ≥2 chronic conditions; MM3+: multimorbidity defined as ≥3 chronic conditions; MM4+: multimorbidity defined as ≥4 chronic conditions; na: not applicable

a The maximum length of lookback period is 5 years for: depression; psychoses; alcohol abuse; drug abuse.

b Shaded areas indicate the length of lookback period where the c-statistic ranged in the standard error interval [±0.001] of the maximal c-statistic.

c The baseline model includes only the covariates (age group, sex, material and social deprivation)

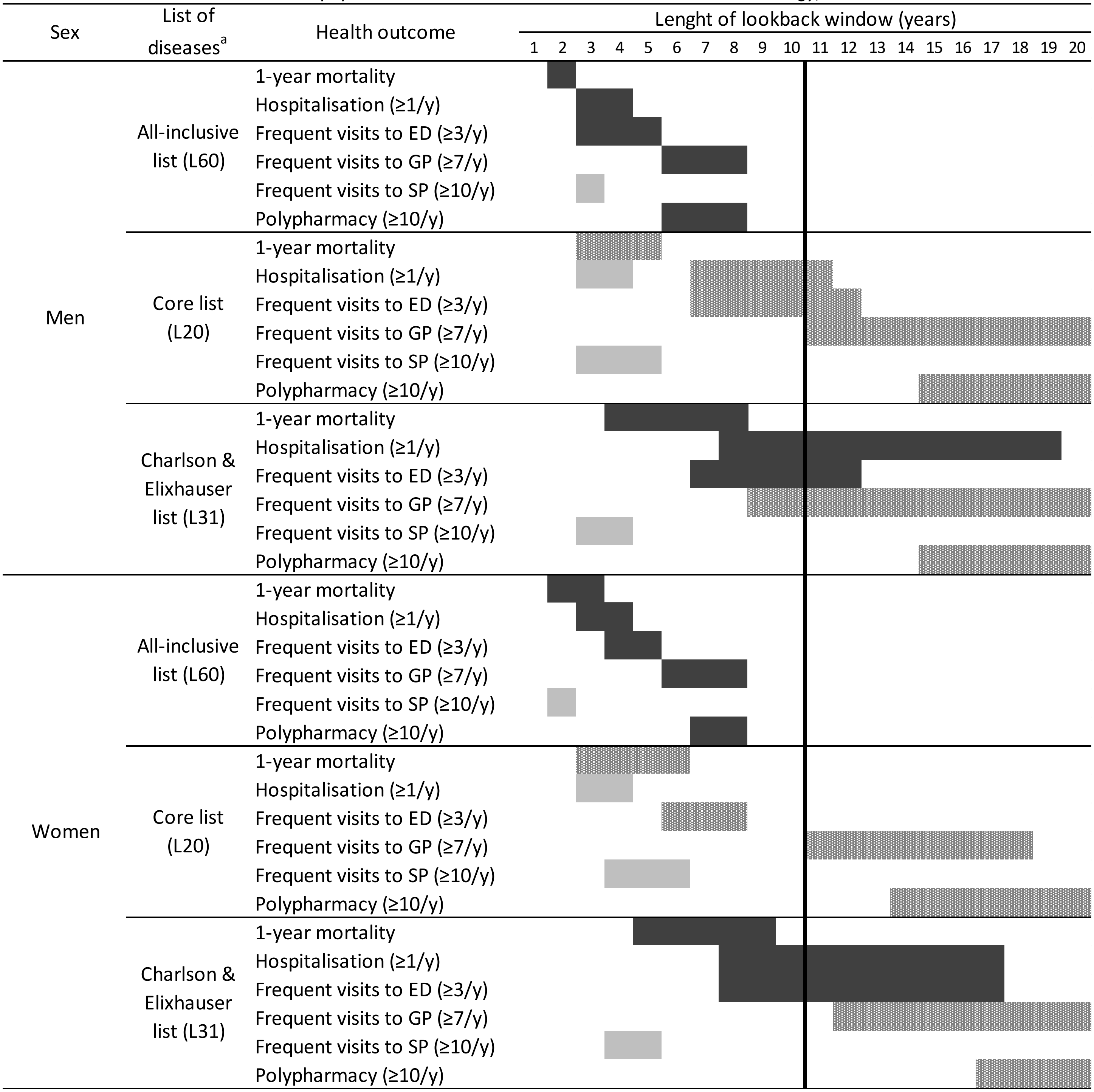

a The All-inclusive list groups all ICD codes of chronic diseases into 60 chronic conditions; the Core list includes 20 chronic diseases associated with a high Disable-adjusted life years (DALY) impact; the Charlson & Elixhauser list combines 31 medical conditions included in both indices

Figure A1.1: Illustration of the length of lookback periods where the predictive performance is maximal according to sex. Light-grey areas indicate maximal predictive performance for the ≥2 chronic conditions (MM2+) definition, grey-dot-pattern areas for the ≥3 chronic conditions (MM3+) definition, and dark-grey areas for the ≥4 chronic conditions (MM4+) definition. Shaded areas indicate the length of lookback period where the *c-statistic* ranged in the standard error interval [±0.001] of the maximal *c-statistic*. The vertical black lines delineate the minimal lookback period (10 years) required to reach a more "stabilized" multimorbidity prevalence.

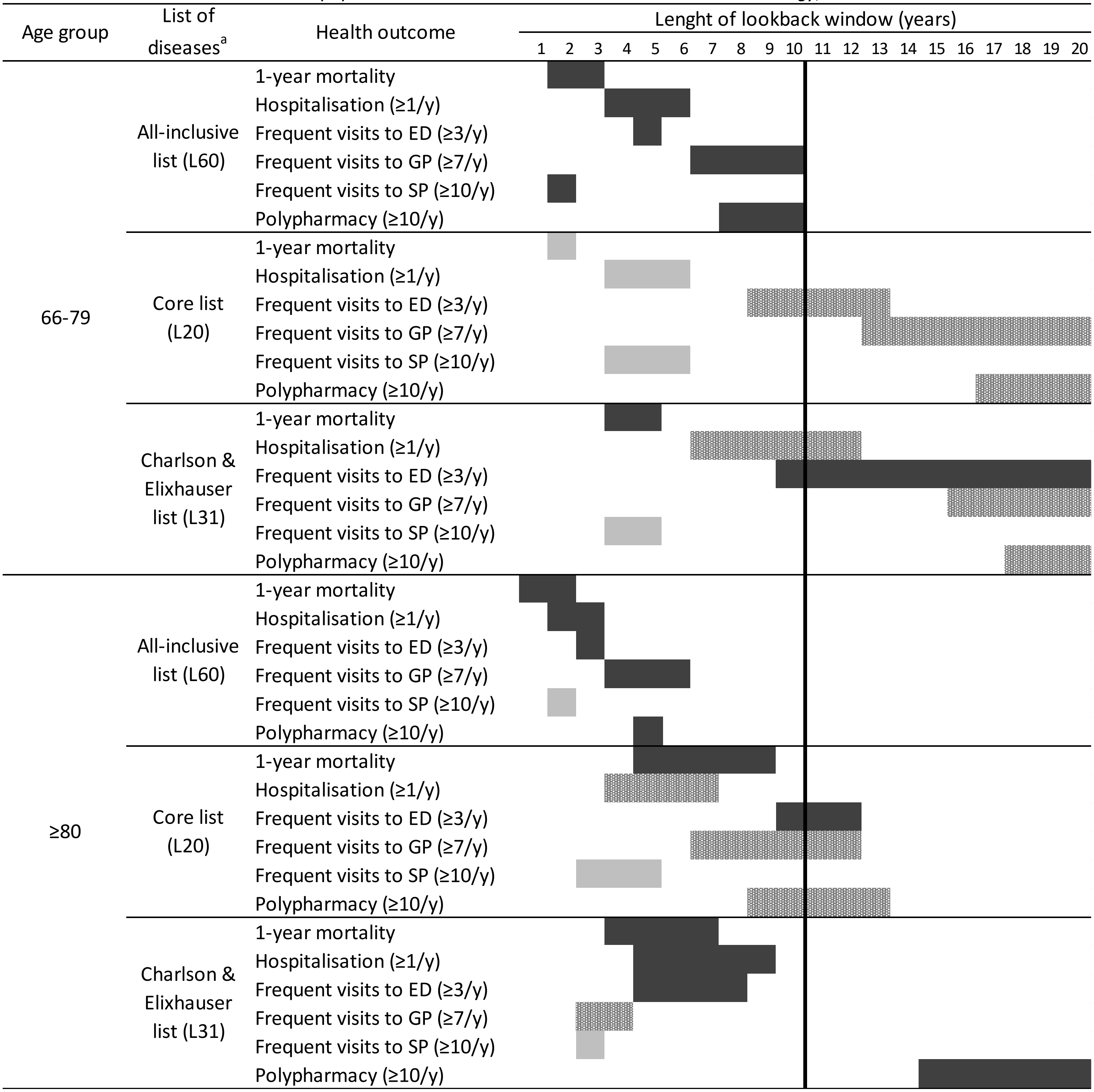

a The All-inclusive list groups all ICD codes of chronic diseases into 60 chronic conditions; the Core list includes 20 chronic diseases associated with a high Disable-adjusted life years (DALY) impact; the Charlson & Elixhauser list combines 31 medical conditions included in both indices

Figure A1.2: Illustration of the length of lookback periods where the predictive performance is maximal according to age group. Light-grey areas indicate maximal predictive performance for the ≥2 chronic conditions (MM2+) definition, grey-dot-pattern areas for the ≥3 chronic conditions (MM3+) definition, and dark-grey areas for the ≥4 chronic conditions (MM4+) definition. Shaded areas indicate the length of lookback period where the *c-statistic* ranged in the standard error interval [±0.001] of the maximal *c-statistic*. The vertical black lines delineate the minimal lookback period (10 years) required to reach a "stabilized" multimorbidity prevalence.

Table A3.1: Maximal predictive performance (c-statistic) value and length of the lookback period when it is reached (year) by sex (>65 years old as of April 1st, 2019, Québec [Canada], n > 1.2 million)

			c-statistic[b] by Health outcomes					
Sex	List of diseases[a]	MM definition	1-year mortality	Hospitalisation (≥1/y)	Frequent visits to ED (≥3/y)	Frequent visits to GP (≥7/y)	Frequent visits to specialist physicians (≥10/y)	Polypharmacy (≥10/y)
Men	All-inclusive list (L60)	MM2+	0.780 (1)	0.659 (1)	0.692 (1)	0.676 (3)	**0.698 (2)**	0.709 (3)
		MM3+	0.786 (1)	0.665 (2)	0.701 (2)	0.682 (4)	0.696 (3)	0.721 (5)
		MM4+	**0.789 (2)**	**0.667 (3)**	**0.707 (3)**	**0.686 (7)**	0.693 (5)	**0.731 (7)**
	Core list (L20)	MM2+	0.788 (2)	0.665 (3)	0.704 (4)	0.689 (6)	**0.680 (4)**	0.740 (7)
		MM3+	**0.788 (4)**	**0.665 (9)**	**0.709 (9)**	**0.692 (16)**	0.673 (12)	**0.742 (20)**
		MM4+	0.783 (8)	0.661 (19)	0.708 (18)	0.687 (20)	0.664 (20)	0.728 (20)
	Charlson & Elixhauser list (L31)	MM2+	0.796 (1)	0.666 (3)	0.705 (3)	0.675 (5)	**0.689 (3)**	0.731 (7)
		MM3+	0.801 (3)	0.669 (6)	0.711 (5)	**0.676 (20)**	0.684 (7)	**0.746 (18)**
		MM4+	**0.802 (5)**	**0.670 (11)**	**0.713 (10**	0.676 (20)	0.678 (17)	0.746 (20)
Women	All inclusive list (L60)	MM2+	0.803 (1)	0.675 (2)	0.700 (2)	0.679 (3)	**0.689 (2)**	0.707 (3)
		MM3+	0.808 (1)	0.683 (3)	0.713 (3)	0.686 (5)	0.688 (4)	0.720 (5)
		MM4+	**0.811 (2)**	**0.687 (4)**	**0.719 (4)**	**0.690 (7)**	0.686 (6)	**0.730 (7)**
	Core list (L20)	MM2+	0.809 (2)	**0.683 (3)**	0.714 (3)	0.693 (6)	**0.667 (5)**	0.727 (7)
		MM3+	**0.810 (4)**	0.682 (7)	**0.718 (6)**	**0.696 (14)**	0.663 (12)	**0.732 (15)**
		MM4+	0.808 (7)	0.679 (13)	0.717 (13)	0.693 (20)	0.656 (20)	0.726 (20)
	Charlson & Elixhauser list (L31)	MM2+	0.818 (2)	0.685 (3)	0.716 (3)	0.673 (6)	**0.674 (4)**	0.729 (9)
		MM3+	0.823 (4)	0.687 (6)	0.720 (5)	**0.676 (19)**	0.669 (10)	**0.744 (19)**
		MM4+	**0.825 (7)**	**0.688 (13)**	**0.722 (10)**	0.675 (20)	0.663 (20)	0.738 (20)

Abbreviation: ED: emergency department; GP: general practitioners; MM2+: multimorbidity defined as ≥2 chronic conditions; MM3+: multimorbidity defined as ≥3 chronic conditions; MM4+: multimorbidity defined as ≥4 chronic conditions; y: year

a The All-inclusive list of diseases groups all ICD codes of chronic diseases into 60 chronic conditions; the Core list includeds20 chronic diseases associated with a high DALY impact; the Charlson & Elixhauser list combines 31 medical conditions included in both indices.

b The c-statistic value in bold indicates the maximal value observed and the length of the lookback period (year) when the maximal c-statisic value is met for a specific health outcome, for each list of diseases

Table A3.2: Maximal predictive performance (c-statistic) value and length of the lookback period when it is reached (year) by age group (>65 years old as of April 1st, 2019, Québec [Canada], n > 1.2 million)

			c-statistic[b] by Health outcomes					
Age group	List of diseases[a]	MM definition	1-year mortality	Hospitalisation (≥1/y)	Frequent visits to ED (≥3/y)	Frequent visits to GP (≥7/y)	Frequent visits to specialist physicians (≥10/y)	Polypharmacy (≥10/y)
66-79	All-inclusive list (L60)	MM2+	0.733 (1)	0.651 (2)	0.680 (2)	0.662 (3)	0.692 (3)	0.701 (3)
		MM3+	0.739 (1)	0.658 (3)	0.692 (3)	0.671 (5)	**0.698 (4)**	0.716 (5)
		MM4+	**0.743 (2)**	**0.660 (5)**	**0.699 (5)**	**0.676 (8)**	0.696 (5)	**0.728 (9)**
	Core list (L20)	MM2+	**0.741 (2)**	**0.657 (4)**	0.693 (4)	0.677 (7)	**0.682 (5)**	0.726 (8)
		MM3+	0.735 (5)	0.655 (10)	**0.697 (11)**	**0.680 (16)**	0.677 (12)	**0.729 (20)**
		MM4+	0.726 (12)	0.672 (13)	0.650 (20)	0.670 (20)	0.668 (20)	0.710 (20)
	Charlson & Elixhauser list (L31)	MM2+	0.764 (1)	0.657 (4)	0.694 (4)	0.655 (7)	**0.690 (4)**	0.729 (10)
		MM3+	0.768 (4)	**0.660 (8)**	0.700 (7)	**0.660 (20)**	0.685 (10)	**0.744 (20)**
		MM4+	**0.764 (8)**	0.660 (19)	**0.701 (15**	0.655 (20)	0.679 (19)	0.733 (20)
≥80	All inclusive list (L60)	MM2+	0.698 (1)	0.624 (1)	0.646 (1)	0.634 (2)	**0.684 (2)**	0.658 (2)
		MM3+	0.708 (1)	0.634 (2)	0.658 (2)	0.640 (3)	0.678 (3)	0.675 (4)
		MM4+	**0.712 (2)**	**0.639 (3)**	**0.663 (3)**	**0.643 (4)**	0.675 (4)	**0.689 (5)**
	Core list (L20)	MM2+	0.711 (1)	0.634 (3)	0.660 (3)	0.650 (4)	**0.657 (3)**	0.693 (5)
		MM3+	0.718 (4)	**0.637 (5)**	0.664 (6)	0.653 (9)	0.654 (7)	**0.700 (9)**
		MM4+	**0.719 (6)**	0.635 (11)	**0.665 (11)**	**0.653 (20)**	0.650 (20)	0.699 (20)
	Charlson & Elixhauser list (L31)	MM2+	0.713 (1)	0.637 (2)	0.660 (3)	0.637 (3)	**0.663 (3)**	0.683 (5)
		MM3+	0.723 (4)	0.642 (4)	0.667 (3)	**0.636 (7)**	0.659 (7)	0.701 (10)
		MM4+	**0.730 (5)**	**0.643 (6)**	**0.669 (6)**	0.636 (11)	0.654 (13)	**0.711 (19)**

Abbreviation: ED: emergency department; GP: general practitioners; MM2+: multimorbidity defined as ≥2 chronic conditions; MM3+: multimorbidity defined as ≥3 chronic conditions; MM4+: multimorbidity defined as ≥4 chronic conditions; y: year

a The All-inclusive list of diseases groups all ICD codes of chronic diseases into 60 chronic conditions; the Core list includes 20 chronic diseases associated with a high DALY impact; the Charlson & Elixhauser list combines 31 medical conditions included in both indices.

b The c-statistic value in bold indicates the maximal value observed and the length of the lookback period (year) when the maximal c-statisic value is met for a specific health outcome, for each list of diseases
